# Substituted spirooxindole derivatives as potent anticancer agents through inhibition of phosphodiesterase 1†

**DOI:** 10.1039/c8ra02358a

**Published:** 2018-04-17

**Authors:** Assem Barakat, Mohammad Shahidul Islam, Hussien Mansur Ghawas, Abdullah Mohammed Al-Majid, Fardous F. El-Senduny, Farid A. Badria, Yaseen A. M. M. Elshaier, Hazem A. Ghabbour

**Affiliations:** Department of Chemistry, College of Science, King Saud University P. O. Box 2455 Riyadh 11451 Saudi Arabia ambarakat@ksu.edu.sa +966-11467-5992 +966-11467-5901; Department of Chemistry, Faculty of Science, Alexandria University P.O. Box 426, Ibrahimia Alexandria 21321 Egypt; Department of Chemistry, Faculty of Science, Mansoura University Mansoura Egypt; Department of Pharmacognosy, Faculty of Pharmacy, Mansoura University Mansoura 35516 Egypt; Pharmaceutical Organic Chemistry Department, Faculty of Pharmacy, Al-Azhar University Assuit 71524 Egypt; Department of Pharmaceutical Chemistry, College of Pharmacy, King Saud University P. O. Box 2457 Riyadh 11451 Saudi Arabia; Department of Medicinal Chemistry, Faculty of Pharmacy, University of Mansoura Mansoura 35516 Egypt

## Abstract

Spirooxindole is a promising chemo therapeutic agent. Possible targets include cancers of the liver, prostate, lung, stomach, colon, and breast. Here, we demonstrate a one-pot three-component reaction *via* a [3 + 2] cycloaddition/ring contraction sequence of a dipolarophile (activated alkene) with *in situ*-generated azomethine ylide (1,3-dipoles) without the use of any catalyst. The reaction provides efficient access to synthetically useful and biologically important spirooxindoles in high yield (69–94%) with high diastereoselectivity. The synthesized compounds were subjected to cytotoxicity evaluation using colorectal cancer (HCT-116), hepatocellular carcinoma (HepG2), and prostate cancer (PC-3) cells. Compounds 4i, 4j, and 4k showed potent cytotoxic activity and high selectivity against HCT-116 cells when compared to cisplatin. Meanwhile compound 4d retained high cytotoxic activity and selectivity against HepG2 and PC-3 cells in comparison to cisplatin. The mechanism of compound 4d was further studied using phosphodiesterase 1 enzyme and showed 74.2% inhibitory activity. A possible binding mode for compound 4d to PDE-1 was investigated by molecular modeling using OpenEye software. Pose predictions for the active compounds were demonstrated by ROCS alignments. Compound 4d has a special geometry and differs from other active compounds.

## Abstract 2

Spirooxindole is a promising chemo therapeutic agent. Possible targets include cancers of the liver, prostate, lung, stomach, colon, and breast.

## Graphical abstract

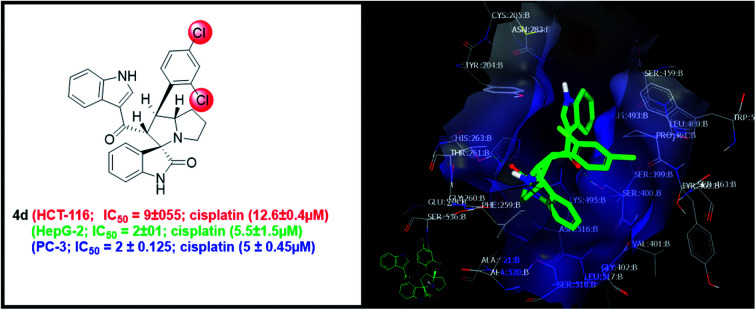

## 1. Introduction

The combined therapy of a multi-kinase inhibitor and a specific phosphodiesterases (PDEs) inhibitor appears to be a good therapy option for tumor treatment, as the tumor growth is delayed and so the chance of survival is increased.^1^ Phosphodiesterases (PDEs) are a ubiquitous family of enzymes that play a role in regulating the intracellular level of the second messengers cyclic adenosine monophosphate (cAMP) and cyclic guanosine monophosphate (cGMP). PDEs are 11 isoenzymes (PDE1–PDE11) and their classification is based on their substrate, amino acid sequence, action or their distribution in the body. These enzymes are important regulators of signal transduction pathways regulating proliferation, apoptosis, differentiation, vasodilation, vasoconstriction and inflammation in cells. In breast and colon cancer cells, the increase in intracellular concentrations of cAMP may induce apoptosis, arrest growth, and reduce cell migration.^2^

PDE-1 catalyzes the hydrolysis of the phosphodiester bond between the catalytic tyrosine residue of topoisomerase I (TOP-1) and DNA 3′-phosphate during gene transcription.^3^ This makes PDE-1 a rational anticancer target. PDE-1 inhibitors have the potential to augment TOP-1 inhibitors as anticancer agents.^4^

Although high intracellular levels of cAMP can effectively inhibit the proliferation of cancer cells, compounds elevating cAMP are not recommended for use as anti-cancer drugs because of their high cytotoxicity.^5–7^ Limited numbers of PDE-1 inhibitors have been reported and there is still an unmet need to discover novel PDE-1 inhibitors.^8,9^ Several studies have been conducted *via* multidisciplinary international research groups to develop more selective and effective potential anti-cancer agents.^10–17^

**Fig. 1:**
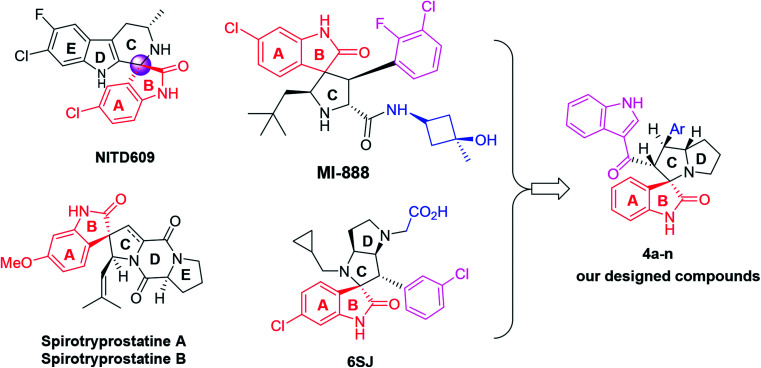
Chemical structures of reported anticancer spirooxindoles and the modified spirooxindole (4a–n).

Spirooxindoles have unique structural features and a ubiquitous class of biological activities, making them promising candidates for new drug discovery.^18^ Over the past decade, this class of compounds has enriched the repertoire of both oxindoles and other heterocyclic scaffolds and has attracted extensive research efforts from synthetic and medical chemists because of their unique chemopreventive properties.^19–21^ Two examples of representative spirooxindole-containing compounds are NITD609 and MI-888 (Fig. 1), which are currently in preclinical evaluation for the treatment of malaria and human cancer, respectively.^22,23^ On the other hand, naturally occurring spirocyclic oxindole alkaloids that could be isolated, such as spirotryprostatins A and B, also show excellent anticancer activities.^24^ Additionally, spirooxindole-containing compounds have been reported to have antimycobacterial^25^ or anti-inflammatory^26^ activities, or can act as an acetylcholinesterase (AChE) inhibitors.^27,28^

The importance of new anticancer agents originating from these spirooxoindole architectures has stimulated our group into designing and synthesizing a new series of spirooxoindoles 4a–n through some modifications in the structure of reported drugs, especially 6SJ^29,30^ (Fig. 1). These structural modifications include: (i) the substituted phenyl moiety tethered ring C was replaced with 3-acyl indole. This replacement is expected to increase the compound's potency, since this scaffold could form extra hydrogen bonding and hydrophobic–hydrophobic interactions. (ii) A substituted or non-substituted aryl group was also installed on ring C. This aryl arm was expected to switch the compound geometry and was suggested to be involved in extra ligand–receptor interactions. Based on the aforementioned information, and in continuation of our previous work,^17,31–33^ we synthesized biologically important, highly substituted, and functionalized spirooxindole derivatives, which are efficient and powerful agents for the treatment of cancer.

## 2. Results and discussion

### 2.1. Synthesis of 4a–n

**Scheme 1:**
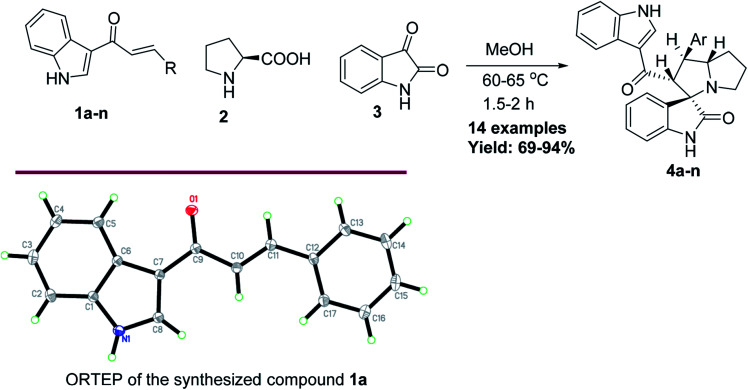
The synthesis of the target spirooxindole derivatives 4a–n.

Anticancer compounds incorporating oxindoles were synthesized using an efficient 1,3-dipolar cycloaddition reaction.^17,31,32^ The starting materials, α,β-unsaturated enone derivatives 1a–n (Scheme 1), were synthesized by a condensation reaction of 3-acetyl indole with substituted aryl aldehydes in the presence of KOH in EtOH under reflux. Then, the one-pot reaction of α,β-unsaturated enone derivatives 1a–n with l-proline 2 and isatin 3 was carried out at 60 °C in MeOH for 1.5–2.0 h to yield the final compound 4a–n, with 4 stereogenic centers in good to excellent yield (69–94%). The molecular structure of the cycloadduct was confirmed by nuclear magnetic resonance (NMR) spectroscopic analysis. The reaction yielded the adduct 4a–n as a single regioisomer. The structures of 4a–n and their derivatives were deduced by ^1^H-NMR, ^13^C-NMR, mass spectrometry (MS), infrared (IR) spectroscopy, elemental analysis, and X-ray crystallography. The absolute configurations for the desired compounds were assigned by X-ray, in addition to spectral analysis studies. The aryl moiety and acylindole originating from ring C were oriented as anti-conformation.

**Scheme 2:**
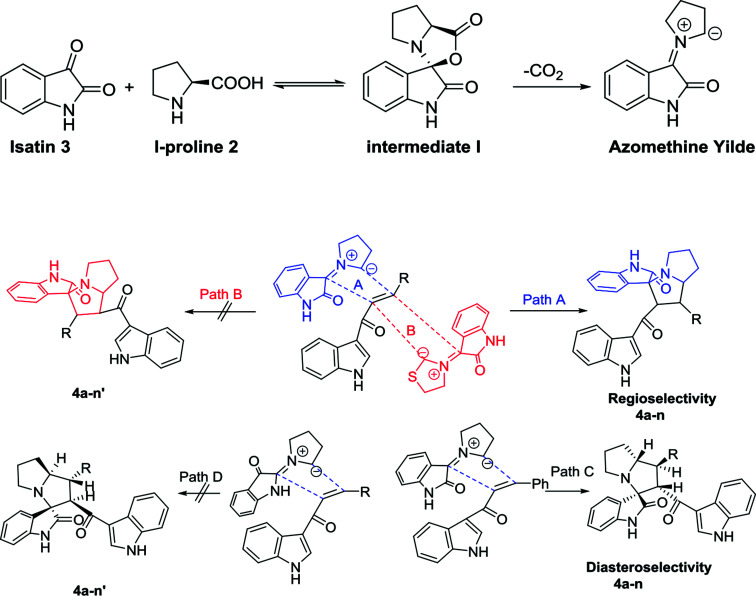
Plausible approach for the target compounds 4a–n.

The proposed reaction mechanism of the three-component reaction is shown in Scheme 2. It was assumed that azomethine ylide was formed exclusively, possibly due to the nucleophilic attack of proline into the active carbonyl of isatin with conversion of the carbonyl group to alcohol. The resulting OH will attack the carboxylic group in proline to form a lactone functionality (intermediate I). *In situ* decarboxylation generates the reactive azomethine ylide. The reaction of olefin with azomethine ylide has four possibilities which will proceed regioselectively with path A exclusively and diastereoselectively with path C to furnish the final adduct (Table 1).^34,35^

**Table 1:** Synthesis of spirooxindole-pyrrolidine 4a–n

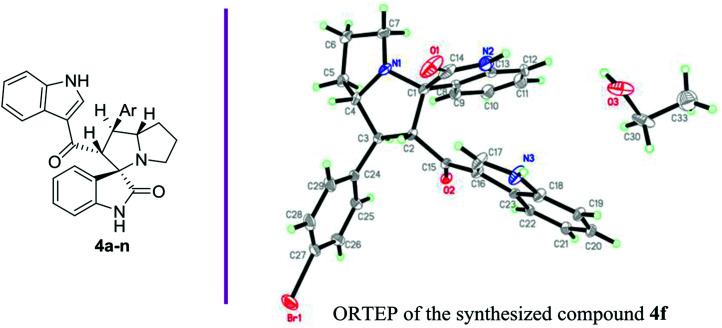			
Entry	4a–n	Ar	Yield (%)
1	4a	C_6_H_5_	84
2	4b	*p*-MeC_6_H_4_	92
3	4c	*p*-ClC_6_H_4_	80
4	4d	2,4-Cl_2_C_6_H_3_	74
5	4e	*p*-MeOC_6_H_4_	86
6	4f	*p*-BrC_6_H_4_	77
7	4g	*p*-FC_6_H_4_	84
8	4h	*m*-FC_6_H_4_	78
9	4i	*m*-MeC_6_H_4_	85
10	4j	*m*-BrC_6_H_4_	72
11	4k	*p*-CF_3_C_6_H_4_	76
12	4l	2-Thiophene	94
13	4m	2-Furan	89
14	4n	3,4,5-Tri-MeOC_6_H_2_	69

### 2.2. Biological activity

Fourteen compounds were tested against three common cancer cell lines, colorectal cancer (HCT-116), hepatocellular carcinoma (HepG2), and prostate cancer (PC-3). Interestingly, most of the tested compounds showed a better selectivity index (SI: >1) over a commonly used chemotherapeutic drug (cisplatin, SI = 0.3). On the other hand, compounds 4i, 4j, and 4k showed a remarkable cytotoxicity against HCT-116 with SI > 2 and IC_50_ at 7, 9 and 9 μM, respectively, in comparison to 12.6 μM for cisplatin, as presented in Table 2.

**Table 2:** The cytotoxic activity and selectivity of the synthesized compounds against a colorectal cancer cell line (HCT-116)^a^

Entry	4a–n	HCT-116 (IC_50_, μM)	HCT-116 (IC_50_, μg ml^−1^)	VERO-B (IC_50_, μM)	SI*
1	4a	ND	ND	ND	ND
2	4b	21 ± 2	9.7	26	1.2
3	4c	20 ± 1.5	9.6	22	1.1
4	4d	9 ± 0.6	4.6	9	1
5	4e	26 ± 2	12.4	26	1
6	4f	21 ± 1.3	11	30	1.4
7	4g	16 ± 1	7.4	40	2.5
8	4h	15 ± 1.4	7	18	1.2
9	4i	7 ± 0.2	3.2	15	2.1
10	4j	9 ± 0.5	4.7	20	2.2
11	4k	9 ± 0.5	4.6	22	2.4
12	4l	50 ± 3.5	22.7	60	1.2
13	4m	29 ± 2	12.7	50	1.7
14	4n	20 ± 1.25	11.1	40	2
Positive control	Cisplatin	12.6 ± 0.40	3.8	5	0.4

a SI*: selectivity index, ND: not determine.

**Fig. 2:**
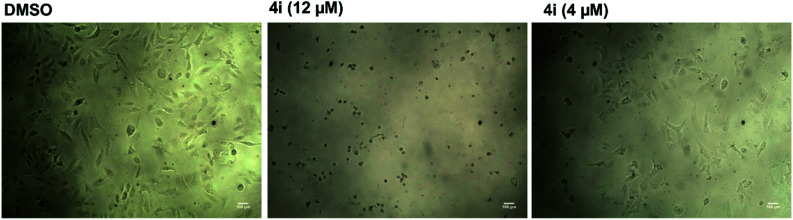
Microscopic examination of the effect of compound 4i on the growth of hepatocellular carcinoma (HepG2).

Meanwhile, compound 4d showed a remarkable cytotoxicity against HepG2 with SI greater than 4 and IC_50_ at 2 μM *versus* 5.5 μM and SI less than 1 for the standard drug cisplatin, as presented in Table 3. In addition, compounds 4c, 4i, and 4j presented similar cytotoxicity against HepG2 with IC_50_ of 8, 7, and 8 μM, respectively, with a better SI > 2 (Table 3 and Fig. 2).

**Table 3:** The cytotoxic activity and selectivity of the synthesized compounds against hepatocellular carcinoma (HepG2) cells^a^

Entry	4a–n	HepG2 (IC_50_, μM)	HepG2 (IC_50_, μg ml^−1^)	VERO-B (IC_50_, μM)	SI*
1	4a	ND	ND	ND	ND
2	4b	11.8 ± 2	5.4	26	2.2
3	4c	8 ± 0.5	3.8	22	2.8
4	4d	2 ± 0.1	1	9	4.5
5	4e	17.3 ± 3	8.3	26	1.5
6	4f	12 ± 1.5	6.3	30	2.5
7	4g	20 ± 2	9.3	40	2
8	4h	14 ± 0.22	6.5	18	1.3
9	4i	7 ± 0.40	3.2	15	2.1
10	4j	8 ± 1	4.2	20	2.5
11	4k	10 ± 1.25	5.2	22	2.2
12	4l	50 ± 3	22.7	60	1.2
13	4m	40 ± 5	17.5	50	1.3
14	4n	28 ± 2	15.7	40	1.4
Positive control	Cisplatin	5.5 ± 1.5	1.7	5	0.91

a SI*: selectivity index, ND: not determine.

Interestingly, compound 4d also showed superior cytotoxic activity against prostate cancer cells at IC_50_ = 2 μM (Table 4), and its selectivity toward the cancer cells was greater than 4, which makes it a promising anticancer candidate. Despite that, compounds 4i, 4j, and 4k showed anticancer activity at a higher IC_50_ (7, 7, and 9 μM, respectively) but their selectivity index was still greater than 2 in comparison to the standard cisplatin (IC_50_ at 5 μM and SI = 1) (Table 4).

**Table 4:** The cytotoxic activity and selectivity of the synthesized compounds against prostate cancer cell line^a^

Entry	4a–n	PC-3 (IC_50_, μM)	PC-3 (IC_50_, μg ml^−1^)	VERO-B (IC_50_, μM)	SI*
1	4a	ND	ND	ND	ND
2	4b	16.3 ± 2	7.5	26	1.6
3	4c	11.8 ± 1.3	5.7	22	1.9
4	4d	2 ± 0.125	1	9	4.5
5	4e	15.5 ± 2	7.4	26	1.7
6	4f	16.3 ± 2.5	8.6	30	1.8
7	4g	16.3 ± 30	7.6	40	2.5
8	4h	11.5 ± 11.5	5.4	18	1.6
9	4i	7 ± 0.6	3.2	15	2.1
10	4j	7 ± 0.2	3.7	20	2.9
11	4k	9 ± 0.2	4.6	22	2.4
12	4l	29 ± 3	13	60	2.1
13	4m	26 ± 1.7	11.3	50	1.9
14	4n	17 ± 2	9.4	40	2.4
Positive control	Cisplatin	5 ± 0.45	1.5	5	1

a SI*: selectivity index, ND: not determine.

### 2.3. Structural activity relationship (SAR)

The study of SAR showed that the *meta* substituted aromatic ring with either methyl or bromine led to an increase in the cytotoxicity of compounds 4i (IC_50_ = 7 μM) and 4j (IC_50_ = 9 μM), respectively, in comparison to the presence of a fluorine atom in compound 4h (IC_50_ = 15 μM). Moreover, the presence of two chlorine atoms in compound 4d at positions C-2 and C-4 in the aromatic ring greatly increased the anticancer activity (IC_50_ = 2 μM) in comparison to one chlorine at the *para* position in compound 4c (IC_50_ = 8 μM). Also, SAR showed that the addition of trifluoromethyl at position C-4 rather than methoxy as in compound 4e (IC_50_ = 26 μM), bromine in compound 4f (IC_50_ = 21 μM) or fluorine in compound 4g (IC_50_ = 16 μM) improved the cytotoxicity of compound 4k (IC_50_ = 9 μM).

### 2.4. Phosphodiesterase inhibitory study

Due to the increase in tumor resistance after a refractory period, there has been extensive research to discover new leads to overcome the resistance and reduce the chemotherapeutic dose in order to decrease the side effects on normal cells. One way is to use phosphodiesterase 1 inhibitors in order to elevate the level of cAMP. Compound 4d, which proved to be the most active and selective anticancer compound among all the tested compounds, showed remarkable inhibitory activity against phosphodiesterase enzyme (PD-1) at 2 μM with 74.2%. Compound 4d could be used in combination with other anticancer drugs such as cisplatin for the treatment of solid tumors. It has been reported that the elevation of cAMP concentration in the cells leads to the inhibition of survival pathways such as MAPK^36^ and antiapoptotic proteins like Bcl-2.^37^ Furthermore, a high level of cAMP could inhibit the interaction of the tumor suppressor p53 protein with its regulator MDM-2.^38^

### 2.5. Molecular docking and shape-matching studies

The crystallographic structure for PDE-1 illustrated that the active site region contains pairs of highly conserved histidine and lysine residues. The active site residues for catalysis are: His 263, Lys 265, His 493, and Lys 495.^39^ The docking of these compounds with PDE-1 exhibited consensus scores and binding modes which correlated with their biological activity as anticancer agents. The target compounds were docked in the active site of PDE-1 (PDB: 1NOP) in order to investigate their binding modes. A library of substituted spirooxindole compounds was designed and energy minimized using MMFF94 force field calculations for the catalytic domain of PDE-1 which was obtained from the protein data bank (PDB code: 1NOP)^40^ and was prepared for docking using OpenEye® software.^41,42^

**Fig. 3:**
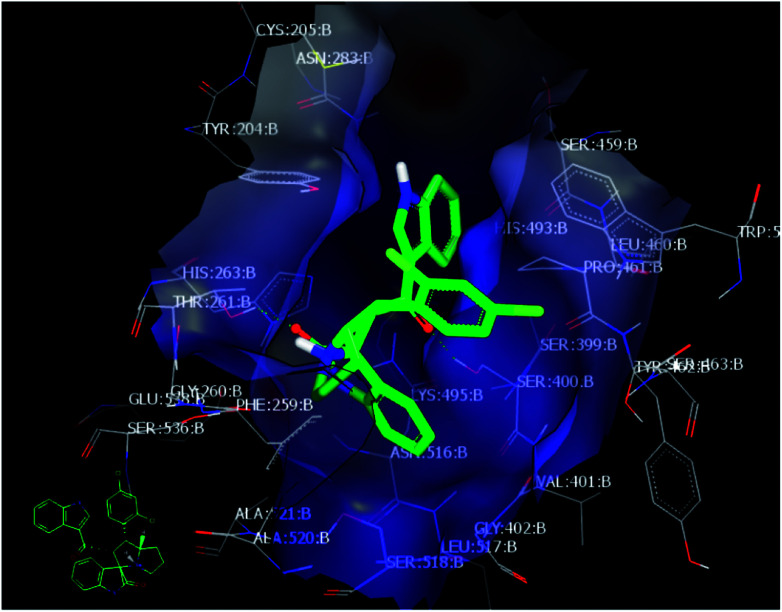
Visual representation of 4d docked with 1NOP showing two HB interactions and hydrophobic–hydrophobic interactions as shown by VIDA.

Among all the compounds, compound 4d showed the best consensus score of 19 by PDE-1 interaction. The pose and mode for compound 4d bound to TDP1 are illustrated in Fig. 3. The carbonyl group of the oxindole scaffold forms hydrogen bonding (HB) interaction with the amino acid Thr 261. This interaction is near the amino acid His 263. The other carbonyl group that links the indole moiety with the remaining part of 4d is also involved in HB interaction with the amino acid Ser 400. The indole part is incorporated near the amino acid Lys 495. The important pharmacophore 2,4-dichlorophenyl π-stacks inside the active site near Ser 463.

Finally, the indole moiety of the compound is directed toward the catalytic core of the enzyme through hydrophobic–hydrophobic interactions with His 493.

**Fig. 4:**
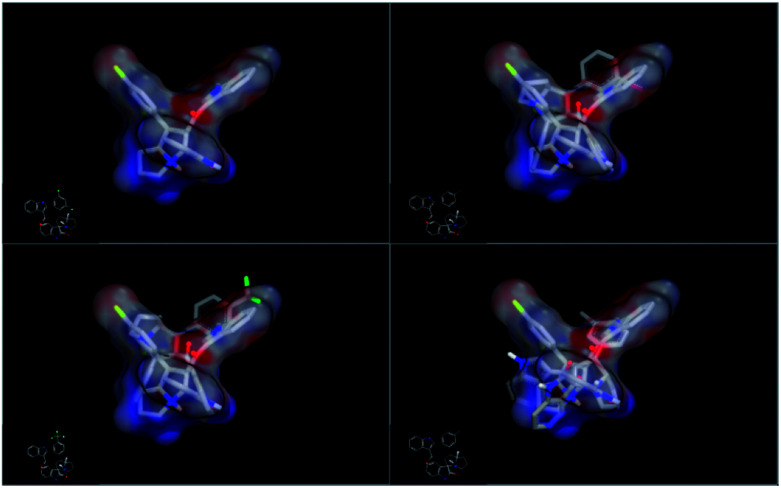
ROCS run for compound 4d with compounds 4k, 4j and 4i with dissimilarity and not completely matched.

In order to understand the diversity of scaffolds in the most active compounds, ROCS alignments for our compounds was performed. ROCS is a fast shape comparison application. It uses a smooth Gaussian function to represent the molecular volume.^43^ ROCS is useful in pose prediction in the absence of a protein structure.^44^ Compound 4d was selected as the query molecule. Other target compounds were selected as the database (dbase.fit) file. The ROCS run for compound 4d with compounds 4k, 4j and 4i represents a dissimilarity between 4d and other compounds (Fig. 4).

**Fig. 5:**
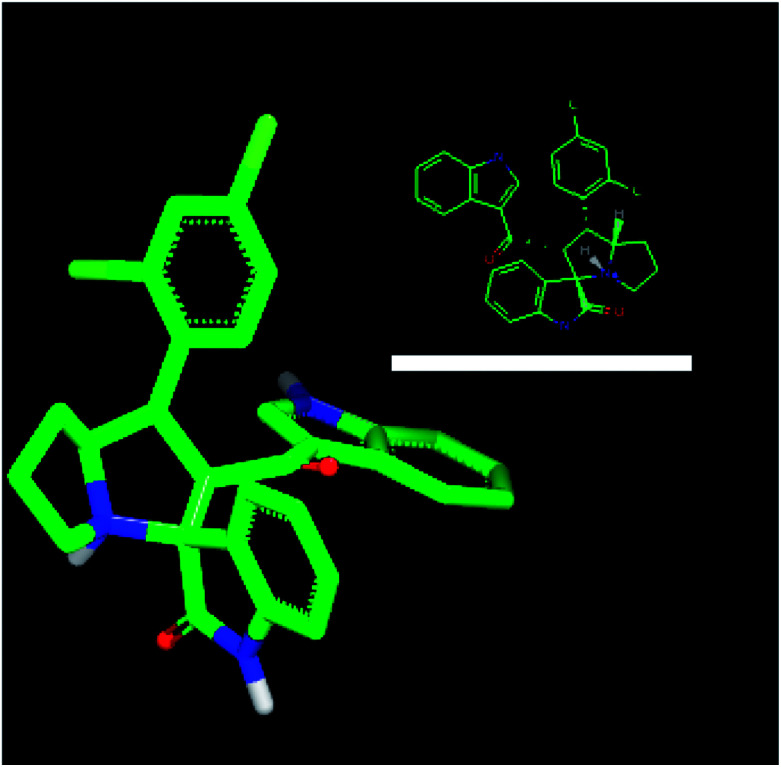
ROCS for 4d with specific geometry of the 2,4-dichlorophenyl and indole moiety perpendicular to each other.

**Fig. 6:**
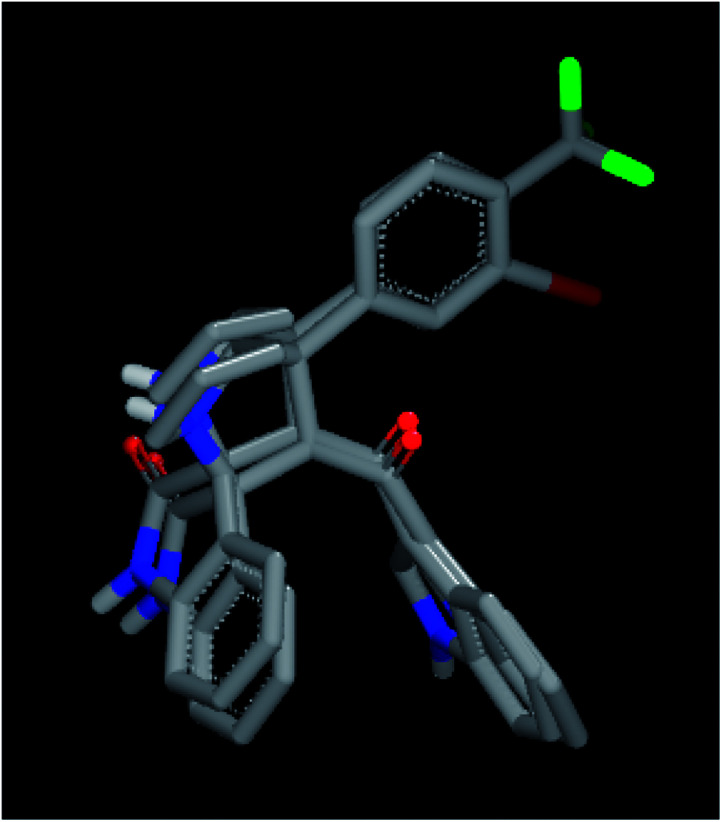
Superposition for compounds 4j and 4k by ROCS exhibited structural similarity.

**Fig. 7:**
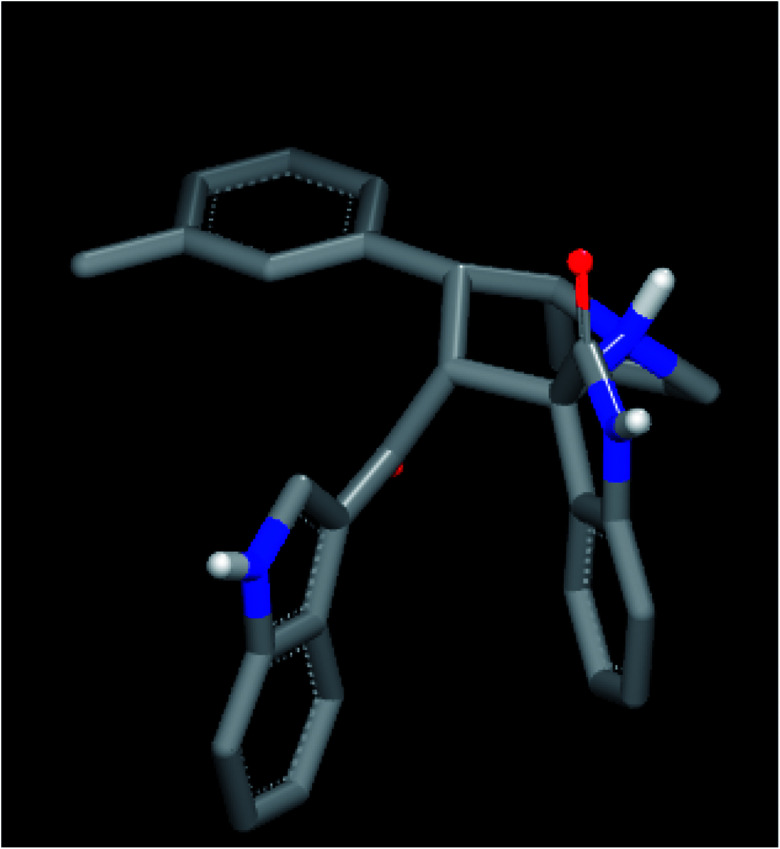
ROCS for compound 4i exhibited a buckled shape with a different pose inside the 1NOP.

For an explanation, compound 4d exhibited a unique orientation in which the 2,4-dichlorophenyl moiety was located axially to the pyrrolidine moiety. The indole part is perpendicular to the oxindole moiety (Fig. 5). Superposition of compounds 4j and 4k (Fig. 6) indicates the chemical similarity between them as the *m*-Br phenyl and *p*-(CF3) phenyl govern the molecular structure. The indole and oxindole moieties face each other with a buckled shape. Compound 4i also adopts a buckled shape in which both indole and oxindole moieties face each other (Fig. 7) but it showed dissimilarities with compounds 4j and 4k.

## 3. Experimental

### 3.1. General procedure (GP1)

Enones 1a–p (0.5 mmol), isatin (74 mg, 0.5 mmol) and l-proline (1.5 mmol) were dissolved in 20 ml of dry MeOH in a 50 ml round-bottom flask. Then, the reaction mixture was heated for 1.5–2 h at 60–65 °C. After the reaction was completed, as monitored by thin-layer chromatography (TLC), the crude material was subjected to column chromatography using ethyl acetate/*n*-hexane (2 : 3), yielding compounds 4a–n.

#### 3.1.1. (2′*R*,3*S*,7*a*′*S*)-2′-(1*H*-Indole-3-carbonyl)-1′-phenyl-1′,2′,5′,6′,7′,7*a*′'-hexahydrospiro[indoline-3,3′-pyrrolizin]-2-one (4a)

Yield, 84%; mp 171 °C; ^1^H-NMR (400 MHz, DMSO-*d*_6_) *δ*: 1.64–1.78 (m, 2H, CH_2_), 1.82–1.90 (m, 2H, CH_2_), 2.32–2.38 (m, 1H, CH_2_), 2.48–2.58 (m, 1H, CH_2_), 3.82–3.92 (m, 1H, CHN), 3.95 (t, 1H, *J* = 10.28 Hz, CHPh), 4.61 (d, 1H, *J* = 11.76 Hz, CHCO), 6.53 (d, 1H, *J* = 7.32 Hz, Ar–H), 6.90 (t, 1H, *J* = 7.32 Hz, Ar–H), 6.92–7.04 (m, 2H, Ar–H), 7.08 (t, 1H, *J* = 7.36 Hz, Ar–H), 7.32 (d, 1H, *J* = 8.04 Hz, Ar–H), 7.36–7.47 (m, 4H, Ar–H), 7.78 (d, 1H, *J* = 8.04 Hz, Ar–H), 7.90 (d, 1H, *J* = 2.92 Hz, Ar–H), 10.27 (s, 1H, NH), 11.82 (s, 1H, NH); ^13^C-NMR (100 MHz, DMSO-*d*_6_) *δ*: 26.9, 29.7, 47.3, 51.5, 63.5, 71.3, 73.4, 109.5, 111.9, 116.7, 119.6, 120.9, 121.2, 121.6, 122.9, 125.1, 125.2, 127.8, 128.8, 129.9, 131.3, 133.5, 136.3, 139.8, 141.6, 179.8, 189; IR (KBr, cm^−1^) *ν*_max_ = 3386, 3248, 2958, 2867, 1716, 1618, 1520, 1470, 1422, 1243, 1137, 1153, 748, 698; [anal. calcd. for C_29_H_25_N_3_O_2_: C, 77.83; H, 5.63; N, 9.39; found: C, 77.75; H, 5.91; N, 9.49]; LC/MS (ESI, *m*/*z*): 447.20 [M + H] for 447.19 C_29_H_25_N_3_O_2_.

#### 3.1.2. (2′*R*,3*S*,7*a*′*S*)-2′-(1*H*-Indole-3-carbonyl)-1′-(*p*-tolyl)-1′,2′,5′,6′,7′,7*a*′-hexahydrospiro[indoline-3,3′-pyrrolizin]-2-one (4b)

Yield (92%); orange powder; mp 174–176 °C; ^1^H-NMR (400 MHz, DMSO-*d*_6_) *δ*: 1.58–1.72 (m, 2H, CH_2_), 1.73–1.86 (m, 2H, CH_2_), 2.13 (s, 3H, CH_3_), 2.25–2.34 (m, 1H, CH_2_), 2.42–2.52 (m, 1H, CH_2_), 3.78–3.83 (m, 1H, CH), 3.84 (t, 1H, *J* = 12.08 Hz, CH), 4.59 (d, 1H, *J* = 11.00 Hz, CH), 6.51 (d, 1H, *J* = 7.36 Hz, Ar–H), 6.88 (t, 1H, *J* = 7.32 Hz, Ar–H), 6.92–7.05 (m, 6H, Ar–H), 7.27 (d, 2H, *J* = 8.80 Hz, Ar–H), 7.34 (d, 1H, *J* = 8.08 Hz, Ar–H), 7.76 (d, 1H, *J* = 8.04 Hz, Ar–H), 7.87 (d, 1H, *J* = 2.92 Hz, Ar–H), 10.25 (s, 1H, NH), 11.78 (s, 1H, NH); ^13^C-NMR (100 MHz, DMSO-*d*_6_) *δ*: 20.6, 27.1, 30.1, 47.3, 52.0, 63.6, 71.6, 73.6, 109.6, 112.0, 117.0, 121.0, 121.3, 121.7, 122.9, 125.2, 125.5, 127.4, 127.9, 128.8, 129.1, 133.4, 135.7, 136.4, 137.3, 147.7, 180.1, 189.4; IR (KBr, cm^−1^) *ν*_max_ = 3381, 3246, 2958, 2865, 1716, 1618, 1581, 1517, 1484, 1423, 1243, 1126, 749; [anal. calcd. for C_30_H_27_N_3_O_2_: C, 78.07; H, 5.90; N, 9.10; found: C, 78.17; H, 5.82; N, 9.23]; LC/MS (ESI, *m*/*z*): 461.20 [M + H] for 461.21 C_30_H_27_N_3_O_2_.

#### 3.1.3. (2′*R*,3*S*,7*a*′*S*)-1′-(4-Chlorophenyl)-2′-(1*H*-indole-3-carbonyl)-1′,2′,5′,6′,7′,7*a*′-hexahydrospiro[indoline-3,3′-pyrrolizin]-2-one (4c)

Yield (80%); beige powder; mp: 165–167 °C; ^1^H-NMR (400 MHz, DMSO-*d*_6_) *δ*: 1.60–1.74 (m, 2H, CH_2_), 1.78–1.88 (m, 2H, CH_2_), 2.28–2.36 (m, 1H, CH_2_), 2.44–2.54 (m, 1H, CH_2_), 3.78–3.88 (m, 1H, CH), 3.94 (t, 1H, *J* = 10.24 Hz, CH), 4.60 (d, 1H, *J* = 11.72 Hz, CH), 6.52 (d, 1H, *J* = 8.08 Hz, Ar–H), 6.89 (t, 1H, *J* = 7.32 Hz, Ar–H), 6.94–7.02 (m, 2H, Ar–H), 7.06 (t, 1H, *J* = 7.32 Hz, Ar–H), 7.30 (t, 3H, *J* = 7.32 Hz, Ar–H), 7.36 (d, 1H, *J* = 7.36 Hz, Ar–H), 7.45 (d, 2H, *J* = 8.76 Hz, Ar–H), 7.77 (d, 1H, *J* = 7.32 Hz, Ar–H), 7.89 (d, 1H, *J* = 3.68 Hz, Ar–H), 10.28 (s, 1H, NH), 11.82 (s, 1H, NH); ^13^C-NMR (100 MHz, DMSO-*d*_6_) *δ*: 27.0, 29.9, 47.36, 51.6, 63.6, 71.5, 73.5, 109.5, 112.0, 116.8, 121.0, 121.3, 121.7, 123.0, 125.2, 125.3, 127.9, 128.5, 128.9, 129.6, 131.3, 131.6, 136.4, 139.4, 141.7, 180.0, 189.2; IR (KBr, cm^−1^) *ν*_max_ = 3247, 2959, 2928, 2868, 1713, 1619, 1521, 1492, 1470, 1424, 1334, 1243, 1138, 750, 532; [anal. calcd. for C_29_H_24_ClN_3_O_2_: C, 72.27; H, 5.02; N, 8.72; found: C, 72.15; H, 5.13; N, 8.86]; LC/MS (ESI, *m*/*z*): 481.21 [M + H] for 481.16 C_29_H_24_ClN_3_O_2_.

#### 3.1.4. (2′*R*,3*S*,7*a*′*S*)-1′-(2,4-Dichlorophenyl)-2′-(1*H*-indole-3-carbonyl)-1′,2′,5′,6′,7′,7*a*′-hexahydrospiro[indoline-3,3′-pyrrolizin]-2-one (4d)

Yield, 74%; beige powder; mp: 149–151 °C; ^1^H-NMR (400 MHz, DMSO-*d*_6_) *δ*: 1.68–1.82 (m, 2H, CH_2_), 1.84–1.92 (m, 2H, CH_2_), 2.34–2.42 (m, 1H, CH_2_), 2.52–2.60 (m, 1H, CH_2_), 3.52–3.62 (m, 1H, CHN), 3.80–3.88 (m, 1H, CHPh), 4.79 (d, 1H, *J* = 11.72 Hz, CHCO), 6.56 (d, 1H, *J* = 7.36 Hz, Ar–H), 6.92–7.11 (m, 4H, Ar–H), 7.34 (d, 2H, *J* = 8.08 Hz, Ar–H), 7.37 (d, 1H, *J* = 2.20 Hz, Ar–H), 7.57 (d, 1H, *J* = 1.84 Hz, Ar–H), 7.78 (t, 2H, *J* = 10.24 Hz, Ar–H), 7.96 (d, 1H, *J* = 2.92 Hz, Ar–H), 10.33 (s, 1H, NH), 11.86 (s, 1H, NH); ^13^C-NMR (100 MHz, DMSO-*d*_6_) *δ*: 26.9, 29.6, 47.2, 51.5, 63.1, 71.9, 73.4, 109.7, 111.8, 116.6, 121.1, 121.2, 121.7, 122.9, 125.0, 125.1, 127.4, 127.8, 128.8, 128.9, 129.0, 129.8, 131.7, 134.7, 136.4, 136.8, 141.8, 179.6, 188.9; IR (KBr, cm^−1^) *ν*_max_ = 3255, 2964, 2869, 1711.1619, 1619, 1520, 1470, 1425, 1335, 1243, 1136, 1110, 1046, 749; [anal. calcd. for C_29_H_23_Cl_2_N_3_O_2_: C, 67.45; H, 4.49; N, 8.14; found: C, 67.55; H, 4.63; N, 8.02]; LC/MS (ESI, *m*/*z*): 515.10 [M + H] for 515.12 C_29_H_23_Cl_2_N_3_O_2_.

#### 3.1.5. (2′*R*,3*S*,7*a*′*S*)-2′-(1*H*-Indole-3-carbonyl)-1′-(4-methoxyphenyl)-1′,2′,5′,6′,7′,7*a*′-hexahydrospiro[indoline-3,3′-pyrrolizin]-2-one (4e)

Yield (86%); yellow powder; mp: 122–124 °C; ^1^H-NMR (400 MHz, DMSO-*d*_6_) *δ*: 1.64–1.76 (m, 2H, CH_2_), 1.80–1.92 (m, 2H, CH_2_), 2.30–2.38 (m, 1H, CH_2_), 2.48–2.56 (m, 1H, CH_2_), 3.52–3.58 (m, 1H, CH), 3.65 (s, 3H, OCH_3_), 3.82–3.86 (m, 1H, CH), 3.89 (t, 1H, *J* = 10.28 Hz, CH), 4.58 (d, 1H, *J* = 11.00 Hz, CH), 6.53 (d, 1H, *J* = 7.32 Hz, Ar–H), 6.83 (d, 2H, *J* = 8.80 Hz, Ar–H), 6.91 (t, 1H, *J* = 7.36 Hz, Ar–H), 6.96–7.04 (m, 2H, Ar–H), 7.07 (t, 1H, *J* = 7.32 Hz, Ar–H), 7.31–7.38 (m, 4H, Ar–H), 7.79 (d, 1H, *J* = 8.04 Hz, Ar–H), 7.88 (d, 1H, *J* = 2.92 Hz, Ar–H), 10.25 (s, 1H, NH), 11.80 (s, 1H, NH); ^13^C-NMR (100 MHz, DMSO-*d*_6_) *δ*: 27.0, 29.9, 47.3, 51.47, 54.9, 63.6, 71.5, 73.4, 109.4, 111.9, 113.9, 116.9, 120.9, 121.3, 121.5, 122.9, 125.1, 125.4, 127.8, 128.5, 128.7, 132.1, 133.4, 136.3, 141.6, 157.9, 179.9, 189.3; IR (KBr, cm^−1^) *ν*_max_ = 3387, 3247, 2960, 2868, 1713, 1618, 1513, 1469, 1437, 1244, 1178, 1138, 1034, 749; [anal. calcd. for C_30_H_27_N_3_O_3_: C, 75.45; H, 5.70; N, 8.80; found: C, 75.31; H, 5.86; N, 9.03]; LC/MS (ESI, *m*/*z*): [477.20 [M + H] for 477.21 C_30_H_27_N_3_O_3_.

#### 3.1.6. (2′*R*,3*S*,7*a*′*S*)-1′-(4-Bromophenyl)-2′-(1*H*-indole-3-carbonyl)-1′,2′,5′,6′,7′,7*a*′-hexahydrospiro[indoline-3,3′-pyrrolizin]-2-one (4f)

Yield (77%); yellow powder; mp: 101–102 °C; ^1^H-NMR (400 MHz, DMSO-*d*_6_) *δ*: 1.64–1.78 (m, 2H, CH_2_), 1.82–1.92 (m, 2H, CH_2_), 2.30–2.38 (m, 1H, CH_2_), 2.49–2.58 (m, 1H, CH_2_), 3.82–3.90 (m, 1H, CH), 3.95 (t, 1H, *J* = 10.28 Hz, CH), 4.64 (d, 1H, *J* = 11.72 Hz, CH), 6.53 (d, 1H, *J* = 7.36 Hz, Ar–H), 6.91 (t, 1H, *J* = 8.04 Hz, Ar–H), 6.95–7.02 (m, 1H, Ar–H), 7.07 (t, 1H, *J* = 7.32 Hz, Ar–H), 7.14 (t, 1H, *J* = 7.36 Hz, Ar–H), 7.26 (t, 2H, *J* = 7.36 Hz, Ar–H), 7.32 (d, 1H, *J* = 8.08 Hz, Ar–H), 7.37 (d, 1H, *J* = 7.36 Hz, Ar–H), 7.42 (d, 2H, *J* = 6.60 Hz, Ar–H), 7.78 (d, 1H, *J* = 8.04 Hz, Ar–H), 7.89 (d, 1H, *J* = 2.92 Hz, Ar–H), 10.26 (s, 1H, NH), 11.81 (s, 1H, NH); ^13^C-NMR (100 MHz, DMSO-*d*_6_) *δ*: 26.9, 29.9, 46.7, 52.1, 63.4, 71.6, 73.4, 109.4, 111.9, 116.8, 120.9, 121.2, 121.5, 122.8, 125.1, 125.4, 126.5, 127.6, 127.8, 128.4, 128.8, 133.4, 136.3, 140.4, 141.3, 179.9, 189.3; IR (KBr, cm^−1^) *ν*_max_ = 3390, 3247, 2962, 2867, 1713, 1618, 1520, 1488, 1470, 1420, 1330, 1243, 1138, 1009, 749; [anal. calcd. for C_29_H_24_BrN_3_O_2_: C, 66.17; H, 4.60; N, 7.98; found: C, 66.28; H, 4.51; N, 8.05]; LC/MS (ESI, *m*/*z*): 525.10 [M + H] for 525.11 C_29_H_24_BrN_3_O_2_.

#### 3.1.7. (2′*R*,3*S*,7*a*′*S*)-1′-(4-Fluorophenyl)-2′-(1*H*-indole-3-carbonyl)-1′,2′,5′,6′,7′,7*a*′-hexahydrospiro[indoline-3,3′-pyrrolizin]-2-one (4g)

Yield (84%); orange powder; mp: 144–146 °C; ^1^H-NMR (400 MHz, DMSO-*d*_6_) *δ*: 1.62–1.76 (m, 2H, CH_2_), 1.80–1.92 (m, 2H, CH_2_), 2.30–2.38 (m, 1H, CH_2_), 2.48–2.56 (m, 1H, CH_2_), 3.80–3.98 (m, 2H, CH), 4.58 (d, 1H, *J* = 10.24 Hz, CH), 6.53 (d, 1H, *J* = 8.08 Hz, Ar–H), 6.81 (d, 1H, *J* = 8.08 Hz, Ar–H), 6.90 (t, 1H, *J* = 7.36 Hz, Ar–H), 6.96–7.11 (m, 4H, Ar–H), 7.28–7.33 (m, 3H, Ar–H), 7.35 (d, 1H, *J* = 6.60 Hz, Ar–H), 7.79 (d, 1H, *J* = 8.04 Hz, Ar–H), 7.88 (d, 1H, *J* = 2.92 Hz, Ar–H), 10.25 (s, 1H, NH), 11.79 (s, 1H, NH); ^13^C-NMR (100 MHz, DMSO-*d*_6_) *δ*: 26.9, 30.64, 47.2, 51.3, 63.6, 71.4, 73.4, 109.4, 111.9, 114.3, 115.0, 115.3, 116.9, 120.9, 121.3, 121.5, 122.8, 125.1, 125.4, 127.8, 128.5, 128.7, 128.8, 129.5, 131.9, 133.3, 136.3, 141.6, 157.2, 179.9, 189.4; IR (KBr, cm^−1^) *ν*_max_ = 3381, 3251, 2960, 2868, 1718, 1618, 1511, 1470, 1422, 1331, 1243, 1178, 1155, 1137, 1044, 861, 749; [anal. calcd. for C_29_H_24_FN_3_O_2_: C, 74.82; H, 5.20; N, 9.03; found: C, 75.02; H, 5.17; N, 9.11]; LC/MS (ESI, *m*/*z*): 465.20 [M + H] for 465.19 C_29_H_24_FN_3_O_2_.

#### 3.1.8. (2′*R*,3*S*,7*a*′*S*)-1′-(3-Fluorophenyl)-2′-(1*H*-indole-3-carbonyl)-1′,2′,5′,6′,7′,7*a*′-hexahydrospiro[indoline-3,3′-pyrrolizin]-2-one (4h)

Yield (78%); yellow powder; mp: 149–151 °C; ^1^H-NMR (400 MHz, DMSO-*d*_6_) *δ*: 1.58–1.72 (m, 2H, CH_2_), 1.75–1.86 (m, 2H, CH_2_), 2.24–2.34 (m, 1H, CH_2_), 2.44–2.53 (m, 1H, CH_2_), 3.79–3.86 (m, 1H, CH), 3.94 (t, 1H, *J* = 11.00 Hz, CH), 4.58 (d, 1H, *J* = 11.72 Hz, CH), 6.48 (d, 1H, *J* = 7.32 Hz, Ar–H), 6.85 (t, 1H, *J* = 8.08 Hz, Ar–H), 6.91–6.98 (m, 3H, Ar–H), 7.02 (t, 1H, *J* = 8.08 Hz, Ar–H), 7.20–7.26 (m, 4H, Ar–H), 7.33 (d, 1H, *J* = 7.32 Hz, Ar–H), 7.73 (d, 1H, *J* = 8.08 Hz, Ar–H), 7.89 (d, 1H, *J* = 3.68 Hz, Ar–H), 10.22 (s, 1H, NH), 11.78 (s, 1H, NH); ^13^C-NMR (100 MHz, DMSO-*d*_6_) *δ*: 26.9, 29.7, 47.3, 51.8, 63.4, 71.3, 73.4, 109.5, 111.9, 113.3, 113.5, 114.4, 114.6, 116.7, 120.9, 121.2, 121.6, 122.9, 123.7, 125.1, 125.3, 127.8, 128.8, 130.3, 130.4, 133.7, 136.4, 141.7, 143.58, 161.0, 163.5, 179.8, 189.2; IR (KBr, cm^−1^) *ν*_max_ = 3388, 3249, 2962, 2867, 1717, 1617, 1587, 1519, 1469, 1424, 1332, 1241, 1142, 748; [anal. calcd. for C_29_H_24_FN_3_O_2_: C, 74.82; H, 5.20; N, 9.03; found: C, 74.93; H, 5.09; N, 9.22]; LC/MS (ESI, *m*/*z*): 465.20 [M + H] for 465.19 C_29_H_24_FN_3_O_2_.

#### 3.1.9. (2′*R*,3*S*,7*a*′*S*)-2′-(1*H*-Indole-3-carbonyl)-1′-(*m*-tolyl)-1′,2′,5′,6′,7′,7*a*′-hexahydrospiro[indoline-3,3′-pyrrolizin]-2-one (4i)

Yield mg (85%); yellow powder; mp: 141–143 °C; ^1^H-NMR (400 MHz, DMSO-*d*_6_) *δ*: 1.62–1.72 (m, 2H, CH_2_), 1.76–1.86 (m, 2H, CH_2_), 2.20 (s, 3H, CH_3_), 2.26–2.36 (m, 1H, CH_2_), 2.46–2.52 (m, 1H, CH_2_), 3.78–3.83 (m, 1H, CH)), 3.87 (t, 1H, *J* = 9.52 Hz, CH), 4.60 (d, 1H, *J* = 11.00 Hz, CH), 6.49 (d, 1H, *J* = 7.32 Hz, Ar–H), 6.87 (t, 1H, *J* = 8.04 Hz, Ar–H), 6.91–7.00 (m, 2H, Ar–H), 7.03 (t, 1H, *J* = 8.04 Hz, Ar–H), 7.10 (t, 2H, *J* = 8.04 Hz, Ar–H), 7.14–7.24 (m, 2H, Ar–H), 7.28 (d, 1H, *J* = 8.08 Hz, Ar–H), 7.33 (d, 1H, *J* = 7.32 Hz, Ar–H), 7.74 (d, 1H, *J* = 8.08 Hz, Ar–H), 7.86 (d, 1H, *J* = 2.92 Hz, Ar–H), 10.21 (s, 1H, NH), 11.76 (s, 1H, NH); ^13^C-NMR (100 MHz, DMSO-*d*_6_) *δ*: 21.0, 26.9, 29.9, 47.2, 52.1, 63.4, 71.6, 73.4, 109.4, 111.9, 116.8, 120.9, 121.2, 121.5, 122.8, 124.6, 125.1, 125.4, 127.2, 127.8, 128.3, 128.4, 128.7, 133.4, 136.3, 137.4, 140.3, 141.6, 180.1, 189.4; IR (KBr, cm^−1^) *ν*_max_ = 3382, 3248, 2957, 2865, 1716, 1618, 1521, 1470, 1422, 1331, 1243, 1152, 1111, 749; [anal. calcd. for C_30_H_27_N_3_O_2_: C, 78.07; H, 5.90; N, 9.10; found: C, 77.89; H, 6.03; N, 9.15]; LC/MS (ESI, *m*/*z*): 461.20 [M + H] for 461.21 C_30_H_27_N_3_O_2_.

#### 3.1.10. (2′*R*,3*S*,7*a*′*S*)-1′-(3-Bromophenyl)-2′-(1*H*-indole-3-carbonyl)-1′,2′,5′,6′,7′,7*a*′-hexahydrospiro[indoline-3,3′-pyrrolizin]-2-one (4j)

Yield (72%); yellow powder; mp: 110–112 °C; ^1^H-NMR (400 MHz, DMSO-*d*_6_) *δ*: 1.65–1.78 (m, 2H, CH_2_), 1.82–1.92 (m, 2H, CH_2_), 2.30–2.38 (m, 1H, CH_2_), 2.50–2.57 (m, 1H, CH_2_), 3.84–3.91 (m, 1H, CH), 3.97 (t, 1H, *J* = 10.24 Hz, CH), 4.62 (d, 1H, *J* = 11.72 Hz, CH), 6.52 (d, 1H, *J* = 7.36 Hz, Ar–H), 6.89 (t, 1H, *J* = 8.08 Hz, Ar–H), 7.00 (q, 2H, *J* = 7.32 Hz, Ar–H), 7.07 (t, 1H, *J* = 8.08 Hz, Ar–H), 7.24 (t, 2H, *J* = 8.08 Hz, Ar–H), 7.13–7.39 (m, 3H, Ar–H), 7.47 (d, 1H, *J* = 8.08 Hz, Ar–H), 7.65 (s, 1H, Ar–H), 7.77 (d, 1H, *J* = 7.32 Hz, Ar–H), 7.92 (d, 1H, *J* = 2.96 Hz, Ar–H), 10.26 (s, 1H, NH), 11.82 (s, 1H, NH); ^13^C-NMR (100 MHz, DMSO-*d*_6_) *δ*: 26.8, 29.6, 47.3, 51.6, 63.5, 71.4, 73.4, 109.5, 111.9, 116.7, 120.9, 121.2, 121.6, 121.8, 122.9, 125.1, 125.2, 126.6, 127.8, 128.9, 129.5, 130.7, 130.8, 133.6, 136.3, 141.6, 143.3, 179.7, 189.1; IR (KBr, cm^−1^) *ν*_max_ = 3403, 3253, 2958, 2866, 1715, 1619, 1520, 1470, 1424, 1332, 1241, 1134, 748; [anal. calcd. for C_29_H_24_BrN_3_O_2_: C, 66.17; H, 4.60; N, 7.98; found: C, 66.06; H, 4.49; N, 7.92]; LC/MS (ESI, *m*/*z*): 525.10 [M + H] for 525.11 C_29_H_24_BrN_3_O_2_.

#### 3.1.11. (2′*R*,3*S*,7*a*′*S*)-2′-(1*H*-Indole-3-carbonyl)-1′-(4-[trifluoromethylphenyl)-1′,2′,5′,6′,7′,7*a*′-hexahydrospiro[indoline-3,3′-pyrrolizin]-2-one (4k)

Yield (76%); yellow powder; mp: 153–155 °C; ^1^H-NMR (400 MHz, DMSO-*d*_6_) *δ*: 1.62–1.80 (m, 2H, CH_2_), 1.82–1.93 (m, 2H, CH_2_), 2.30–2.40 (m, 1H, CH_2_), 2.50–2.58 (m, 1H, CH_2_), 3.86–3.94 (m, 1H, CH), 4.07 (t, 1H, *J* = 11.00 Hz, CH), 4.67 (t, 1H, *J* = 11.76 Hz, CH), 6.53 (d, 1H, *J* = 7.36 Hz, Ar–H), 6.90 (t, 1H, *J* = 7.32 Hz, Ar–H), 6.95–7.07 (m, 3H, Ar–H), 7.33 (d, 1H, *J* = 5.84 Hz, Ar–H), 7.39 (d, 1H, *J* = 7.36 Hz, Ar–H), 7.55–7.72 (m, 4H, Ar–H), 7.76 (d, 1H, *J* = 8.08 Hz, Ar–H), 7.887791 (d, 1H, *J* = 3.68 Hz, Ar–H), 10.28 (s, 1H, NH), 11.83 (s, 1H, NH); ^13^C-NMR (100 MHz, DMSO-*d*_6_) *δ*: 26.9, 29.7, 47.2, 51.8, 63.4, 71.4, 73.4, 79.1, 109.4, 111.9, 116.6, 120.9, 121.2, 121.6, 122.9, 125.0, 125.2, 125.3, 127.5, 127.6, 128.6, 128.8, 133.6, 136.3, 141.6, 145.3, 179.7, 189.0; IR (KBr, cm^−1^) *ν*_max_ = 3254, 2960, 2869, 1716, 1619, 1521, 1470, 1423, 1325, 1165, 1116, 1068, 1017, 7450; [anal. calcd. for C_30_H_24_F_3_N_3_O_2_: C, 69.89; H, 4.69; N, 8.15; found: C, 70.07; H, 4.82; N, 8.01]; LC/MS (ESI, *m*/*z*): 515.20 [M + H] for 151.18 C_30_H_24_F_3_N_3_O_2_.

#### 3.1.12. (2′*R*,3*S*,7*a*′*S*)-2′-(1*H*-Indole-3-carbonyl)-1′-(thiophen-2-yl)-1′,2′,5′,6′,7′,7*a*′-hexahydrospiro[indoline-3,3′-pyrrolizin]-2-one (4l)

Yield (94%); yellow powder; mp: 157–159 °C; ^1^H-NMR (400 MHz, DMSO-*d*_6_) *δ*: 1.68–1.82 (m, 2H, CH_2_), 1.82–1.91 (m, 1H, CH_2_), 1.92–2.02 (m, 1H, CH_2_), 2.28–2.38 (m, 1H, CH_2_), 2.48–2.56 (m, 1H, CH_2_), 3.88–3.98 (m, 1H, CH), 4.21 (t, 1H, *J* = 9.52 Hz, CH), 4.50 (d, 1H, *J* = 11.76 Hz, CH), 6.52 (d, 1H, *J* = 7.32 Hz, Ar–H), 6.89–6.92 (m, 2H, Ar–H), 6.96–7.03 (m, 3H, Ar–H), 7.11 (t, 1H, *J* = 6.60 Hz, Ar–H), 7.28 (d, 1H, *J* = 5.12 Hz, Ar–H)), 7.32–7.35 (m, 2H, Ar–H), 7.82 (d, 1H, *J* = 8.08 Hz, Ar–H), 7.85 (d, 1H, *J* = 2.92 Hz, Ar–H), 10.27 (s, 1H, NH), 11.85 (s, 1H, NH); ^13^C-NMR (100 MHz, DMSO-*d*_6_) *δ*: 26.9, 30.0, 47.3, 64.6, 64.9, 71.5, 73.5, 109.5, 111.9, 116.8, 120.9, 121.3, 121.6, 122.9, 123.9, 124.4, 125.0, 125.1, 126.9, 127.8, 128.9, 133.4, 136.4, 141.6, 143.4, 179.7, 188.9; IR (KBr, cm^−1^) *ν*_max_ = 3382, 3250, 2963, 2963, 2867, 1717, 1620, 1521, 1469, 1426, 1332, 1421, 1132, 749, 698; [anal. calcd. for C_27_H_23_N_3_O_2_S: C, 71.50; H, 5.11; N, 9.26; found: C, 71.37; H, 4.97; N, 9.04]; LC/MS (ESI, *m*/*z*): 453.10 [M + H] for 453.10 C_27_H_23_N_3_O_2_S.

#### 3.1.13. (2′*R*,3*S*,7*a*′*S*)-1′-(Furan-2-yl)-2′-(1*H*-indole-3-carbonyl)-1′,2′,5′,6′,7′,7*a*′-hexahydrospiro[indoline-3,3′-pyrrolizin]-2-one (4m)

Yield (89%); beige powder; mp: 172–174 °C; ^1^H-NMR (400 MHz, DMSO-*d*_6_) *δ*: 1.72–1.96 (m, 3H, CH_2_), 2.00–2.10 (m, 1H, CH_2_), 2.34–2.42 (m, 1H, CH_2_), 2.48–2.54 (m, 1H, CH_2_), 3.92–4.00 (m, 1H, CH), 4.09 (t, 1H, *J* = 9.56 Hz, CH), 4.65 (d, 1H, *J* = 11.72 Hz, CH), 6.24 (d, 1H, *J* = 2.88 Hz, Ar–H), 6.35 (t, 1H, *J* = 2.20 Hz, Ar–H), 6.57 (d, 1H, *J* = 7.32 Hz, Ar–H), 6.95 (t, 1H, *J* = 7.32 Hz, Ar–H), 7.06–7.10 (m, 2H, Ar–H), 7.16 (t, 1H, *J* = 7.32 Hz, Ar–H), 7.35 (d, 1H, *J* = 7.32 Hz, Ar–H), 7.42 (d, 1H, *J* = 8.04 Hz, Ar–H), 7.56 (d, 1H, *J* = 1.48 Hz, Ar–H), 7.88 (d, 1H, *J* = 8.04 Hz, Ar–H), 7.94 (d, 1H, *J* = 2.92 Hz, Ar–H), 10.32 (s, 1H, NH), 11.91 (s, 1H, NH); ^13^C-NMR (100 MHz, DMSO-*d*_6_) *δ*: 26.9, 30.3, 45.4, 47.2, 61.2, 68.8, 73.2, 105.5, 109.5, 110.3, 111.9, 116.5, 120.9, 121.3, 121.6, 125.1, 125.2, 127.8, 128.8, 133.4, 136.8, 141.6, 141.9154.0, 179.6, 188.8; IR (KBr, cm^−1^) *ν*_max_ = 3401, 3242, 2961, 2872, 1721, 1630, 1617, 1522, 1471, 1422, 1244, 1150, 1130, 1113, 1011, 751; [anal. calcd. for C_27_H_23_N_3_O_3_: C, 74.12; H, 5.30; N, 9.60; found: C, 73.98; H, 5.47; N, 9.51]; LC/MS (ESI, *m*/*z*): 437.20 [M + H] for 437.17 C_27_H_23_N_3_O_3_.

#### 3.1.14. (2′*R*,3*S*,7*a*′*S*)-2′-(1*H*-Indole-3-carbonyl)-1′-(3,4,5-trimethoxyphenyl)-1′,2′,5′,6′,7′,7*a*′-hexahydrospiro[indoline-3,3′-pyrrolizin]-2-one (4n)

Yield (69%); yellow powder; mp: 179–181 °C; ^1^H-NMR (400 MHz, DMSO-*d*_6_) *δ*: 1.64–1.82 (m, 2H, CH_2_), 1.82–1.94 (m, 2H, CH_2_), 2.32–2.40 (m, 1H, CH_2_), 2.50–2.58 (m, 1H, CH_2_), 3.56 (s, 3H, OCH_3_), 3.70–3.72 (m, 1H, CH), 3.74 (s, 6H, 2xOCH_3_), 3.89–3.91 (m, 1H, CH), 4.64–4.67 (m, 1H, CH), 6.53 (d, 1H, *J* = 8.08 Hz, Ar–H), 6.73 (s, 2H, Ar–H), 6.91 (t, 1H, *J* = 7.32 Hz, Ar–H), 6.96–7.05 (m, 2H, Ar–H), 7.08 (t, 1H, *J* = 7.32 Hz, Ar–H), 7.33 (d, 1H, *J* = 8.04 Hz, Ar–H), 7.41 (d, 1H, *J* = 7.36 Hz, Ar–H), 7.79 (d, 1H, *J* = 8.04 Hz, Ar–H), 7.99 (d, 1H, *J* = 2.92 Hz, Ar–H), 10.23 (s, 1H, NH), 11.80 (s, 1H, NH); ^13^C-NMR (100 MHz, DMSO-*d*_6_) *δ*: 26.9, 29.9, 47.3, 52.4, 55.8, 59.8, 63.2, 71.4, 73.6, 104.9, 109.4, 111.9, 116.9, 120.8, 121.2, 121.5, 122.8, 125.1, 125.4, 127.8, 128.7, 133.6, 136.0, 136.2, 136.4, 141.6, 152.8, 175.5, 189.4; IR (KBr, cm^−1^) *ν*_max_ = 3256, 2937, 2870, 1720, 1619, 1590, 1510, 1468, 1427, 1332, 1243, 1187, 1154, 1126, 9997, 788, 743; [anal. calcd. for C_32_H_31_N_3_O_5_: C, 71.49; H, 5.81; N, 7.82; found: C, 71.35; H, 5.92; N, 7.96]; LC/MS (ESI, *m*/*z*): 537.20 [M + H] for 537.23 C_32_H_31_N_3_O_5_.

### 3.2. Anticancer activity

#### 3.2.1. Cell lines and drugs

The cytotoxic activity of the compounds was tested against different mammalian cancer cells, prostate carcinoma cells (PC-3), hepatocellular carcinoma (HepG2) and colon cancer cells (HCT-116). African green monkey kidney cells (Vero-B) were used as normal cells to study the selectivity towards the cancer cells. The cell lines were obtained from the American Type Culture Collection (ATCC). The cells were cultivated at 37 °C and 10% CO_2_ in DMEM (Lonza, Germany) medium supplemented with 10% fetal bovine serum (Lonza, Germany), 100 IU ml^−1^ penicillin and 100 μg ml^−1^ streptomycin (Lonza, Germany). Cisplatin (*cis*-diamineplatinum(ii) dichloride) was used as a positive control and was obtained from Sigma-Aldrich®, then dissolved in 0.9% saline and stored as an 8 mM stock solution at −20 °C. The spirooxindole derivatives were solubilized in DMSO and stored at −20 °C. The viability of the cells was quantified using 3-(4,5-dimethylthiazol-2-yl)-2,5-diphenyl tetrazolium bromide (MTT), which measures the activity of mitochondrial succinate dehydrogenase in viable cells.^45,46^

#### 3.2.2. Cytotoxicity assay

The cells were seeded in 96-well plates at a concentration of 5 × 10^4^ cells per ml (100 μl per well). A serial dilution of tested compounds or cisplatin was added after the cells were incubated overnight at 37 °C and under 5% CO_2_. DMSO was used as a negative control (0.1%). The cells were incubated for 48 h. After that, 15 μl of MTT (5 mg ml^−1^ in PBS) was added to each well and incubated for another 4 h. The formazan crystals were solubilized by 100 μl of acidified SDS solution (10% SDS/0.01 N HCl in PBS). The absorbance was measured after 14 h of incubation at 37 °C and under 5% CO_2_ at 570 nm by using a BioTek microplate reader. Each experiment was repeated 3 times and a standard deviation was calculated (SD±). IC_50_ was calculated as the concentration that caused 50% inhibition of cell growth. The growth of the cells was monitored and the images were acquired by Gx microscopes (GXMGXD202 Inverted Microscope) at 10× magnification.

#### 3.2.3. Selectivity index (SI) calculations

The selectivity index was calculated with the following equation1
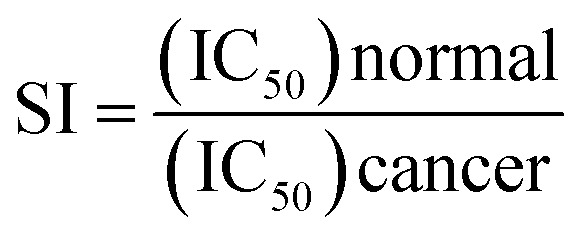
Eqn (1): selectivity index (SI) equation: where IC_50_ normal = the concentration of the tested compound that killed 50% of normal cells; IC_50_ cancer = the concentration of the same tested compound that killed 50% of cancer cells.

#### 3.2.4. Phosphodiesterase I inhibition assay

A phosphodiesterase I inhibition assay was performed using snake venom according to a previously reported method with minute variations. Briefly, 33 mM Tris–HCl buffer of pH 8.8 (97 μl), 30 mM magnesium acetate with an enzyme concentration of 0.000742 U well^−1^ and 0.33 mM bis-(*p*-nitrophenyl) phosphate (Sigma N-3002, 60 μl) as substrate were taken. EDTA with an IC_50_ ± SD of 274 ± 0.007 μM was used as the positive control. After a pre-incubation period of 30 min, the enzyme with the test samples was observed spectrophotometrically for enzyme activity on a microtiter plate reader at 37 °C by following the rate of change in OD min^−1^ at 410 nm of the *p*-nitrophenol released from *p*-nitrophenyl phosphate. All assays were processed in triplicate.^47^

#### 3.2.5. Docking studies

The docking studies were performed using OpenEye Modelling software. A virtual library of spirooxindoles derivatives was used and their energies were minimized using the MMFF94 force field, followed by the generation of multi-conformers using the OMEGA application.^42^ The whole library of minimized energy values was docked along with the prepared PDE-1 (PDB ID: 1NOP)^40–48^ using the FRED application to generate a physical property (Δ*G*) reflecting the predicted energy profile of the ligand-receptor complex. For ROCS study, the most active compound was selected as the query molecule. A library of compounds was adopted as the database (fit) file. The VIDA application^49^ was employed as a visualization tool to show the poses of the ligands and the potential binding interactions of the ligands to the receptor of interest.

## 4. Conclusions

In summary, inspired by synthesized spirooxindoles and natural architectures, we have succeeded in generating potent anticancer derivatives. The *in vitro* study revealed highly selective anticancer agents with a much better cytotoxic activity against colorectal cancer (HCT-116), hepatocellular carcinoma (HepG2), and prostate cancer (PC-3) when compared to the commonly used chemotherapeutic cisplatin. In the phosphodiesterase 1 enzyme inhibition studies, compound 4d proved to exhibit a high cytotoxic activity against colorectal, prostate, and liver cancers at IC_50_ = 9, 2, and 2 μM with selectivity indices >1, >4, and >4, respectively. Moreover, compound 4d showed the best interaction with PDE-1 with a consensus score of 19. It formed two HB interactions and also hydrophobic interactions. The ROCS of this newly synthesized drug candidate adopted a unique geometry unlike other derivatives. The aryl arm controls the geometry of the compounds and in the case of 4d the 2,4-dichlorophenyl moiety allowed the indole and oxoindole moieties to occupy space perpendicularly. This unique character, high selectivity and promising activity against three aggressive cancer cell lines of compound 4d make it a promising anticancer candidate. Therefore, this compound should be considered a potential anti-cancer agent in combination with widely used chemotherapeutic drugs to improve the response of tumors. Currently, a more rigorous *in vivo* study is being undertaken to disclose more preclinical information, such as oral stability, bioavailability, and pharmacokinetics with the anticipation of better activity and high safety margins.

## Conflicts of interest

There are no conflicts to declare.

## Supplementary Material

RA-008-C8RA02358A-s001

RA-008-C8RA02358A-s002
