# Supplementary material for: Substituted spirooxindole derivatives as potent anticancer agents through inhibition of phosphodiesterase 1
Source: RSC Adv. 2018 Apr 17;8(26):14335–46. doi: 10.1039/c8ra02358a (PMC9079959; doi:10.1039/c8ra02358a)
Supplement: RA-008-C8RA02358A-s001 [file RA-008-C8RA02358A-s001.pdf]

Electronic Supplementary Material (ESI) for RSC Advances.
This journal is © The Royal Society of Chemistry 2018

SUPPORTING INFORMATION

Table of contents

1. General method for the synthesis of chalcone **1a-n**
2. Copies of NMR spectrum of **4a-n**

1. General method for the synthesis of chalcone **1a-n**

The 3-acetyl-indole (1.59 g, 10 mmol) was dissolved in ethanol 100 mL, in a 250 mL round bottom flask equipped with condenser and inert atmosphere. Aldehyde (2 eq) and KOH (2 eq) in 10 mL water were added to the reaction mixture. The resulting reaction mixture were heated to reflux for 24 hours. Then the solvent was removed under reduced pressure and the crude product were subjected to column chromatography using DCM as an eluent. Finally the products **1a-n** were further purified by ether washing.

(*E*)-1-(1-*H*-Indol-3-yl)-3-phenylprop-2-en-1-one (**1a**)

Yellow powder; m.p. 219-221 ºC; ^{1}H-NMR (400 MHz, DMSO-d_6) δ: 7.20-7.24 (m, 2H, Ar-H), 7.40-7.50 (m, 4H, Ar-H), 7.62 (d, 1H, J = 15.4 Hz, COCH=CH), 7.82-7.85 (t, 3H, J = 8.04 Hz, Ar-H & COCH=CH), 8.33 (d, 1H, J = 7.32 Hz, Ar-H), 8.74 (s, 1H, Ar-H), 12.10 (s, 1H, NH); ^{13}C-NMR (100 MHz, DMSO-d_6) δ: 112.2, 117.7, 121.7, 121.8, 123.1, 124.6, 125.8, 128.4, 128.8, 129.7, 134.7, 135.2, 136.8, 139.5, 183.55; IR (KBr, cm^{-1}) ν_{max}= 3414(NH), 3135(C-H), 2924, 2864, 2590, 1639(C=O),1513(C=C), 1442, 1150, 974, 745; [Anal. Calcd. for $C_{17}H_{13}NO$: C, 82.57; H, 5.30; N, 5.66; Found: C, 82.62; H, 5.28; N, 5.56]; LC/MS (ESI, *m/z*): 247.10 [M+] for 247.10 $C_{17}H_{13}NO$.

(*E*)-1-(1-*H*-Indol-3-yl)-3-(*p*-tolyl)prop-2-en-1-one (**1b)**

Yellow powder; m.p. 207-209 ºC; ^{1}H-NMR (400 MHz, DMSO-d_6) δ: 2.34 (s, 3H, CH_3), 7.19 – 7.26 (m, 4H, Ar-H), 7.47 (d, 1H, J = 13.92 Hz,COCH=CH), 7.58 (d, 1H, J = 15.4 Hz, Ar-H), 7.65 – 7.79 (t, 3H, J = 8.08 Hz, Ar-H & COCH=CH), 8.31 (d, 1H, J = 6.60 Hz, Ar-H), 8.70 (s, 1H, Ar-H), 12.09 (s, 1H, NH);^{13}C-NMR (100 MHz, DMSO-d_6) δ: 112.2, 117.7, 121.7, 121.9, 123.2, 123.6, 125.9, 128.4, 129.5, 132.5, 134.5, 134.7, 136.8, 139.6, 183.7; IR (KBr, cm^{-1}) ν_{max}= 3157(NH), 2862(C-H), 2361, 1641(C=O),1568(C=C), 1517, 1441, 1147, 973; [Anal. Calcd. for $C_{18}H_{15}NO$: C, 261.12; H, 5.79; N, 5.36; Found: C, 261.10; H, 5.91; N, 5.35]; LC/MS (ESI, *m/z*): 216.10 [M+] for 216.12 $C_{18}H_{15}NO$.

(*E*)-3-(4-Chlorophenyl)-1-(1-*H*-indol-3-yl)prop-2-en-1-one (**1c)**

Yellow powder; m.p: 237-239ºC; ^{1}H-NMR (400 MHz, DMSO-d_6) δ: 7.20 – 7.25 (m, 3H, Ar-H), 7.45 – 7.53 (m, 4H, Ar-H), 7.61 (d, 1H, J = 15.4 Hz, COCH=CH), 7.84-7.90 (m, 2H, Ar-H & COCH=CH), 8.33 (d, 1H, J = 8.08 Hz, Ar-H), 8.74 (s, 1H, Ar-H), 12.13 (s, 1H, NH); ^{13}C-NMR (100 MHz, DMSO-d_6) δ: 112.2, 117.7, 121.7, 121.9, 123.2, 124.3, 125.4, 125.8, 128.8, 130.1, 134.2, 135.0, 136.9, 138.1, 183.4; IR (KBr, cm^{-1}) ν_{max}=

3144(NH), 2925(C-H), 2862, 2365, 1638(C=O),1570(C=C), 1516, 1440, 1150, 976, 749; [Anal. Calcd. for $C_{17}H_{12}ClNO$: C, 72.47; H, 4.29; N, 4.97; Found: C, 72.38; H, 4.30; N, 4.87]; LC/MS (ESI, *m/z*): 281.10 [M+] for 281.06 $C_{17}H_{12}ClNO$.

(*E*)-3-(2,4-Dichlorophenyl)-1-(1-*H*-indol-3-yl)prop-2-en-1-one (**1d)**

Yellow powder; m.p: 244-247°C; ^{1}H-NMR (400 MHz, DMSO-d_6) δ: 7.19-7.30 (m, 2H, Ar-H), 7.51 (t, 2H, J = 8.84 Hz, Ar-H), 7.7 (s, 1H, Ar-H), 7.72 – 7.89 (m, 2H, Ar-H & COCH=CH), 8.21 (d, 1H, J = 8.08 Hz, COCH=CH), 8.31 (d, 1H, J = 7.36 Hz, Ar-H), 8.75 (s, 1H, Ar-H), 12.10 (s, 1H, NH); ^{13}C-NMR (100 MHz, DMSO-d_6) δ: 112.42, 117.6, 121.6, 122.0, 123.3, 126.0, 127.8, 128.2, 129.4, 132.0, 133.0, 134.7, 134.8, 135.6, 135.7, 137.2, 182.8; IR (KBr, cm^{-1}) ν_{max}= 3435(NH), 2932(C-H), 2869, 2594, 1636(C=O), 1574(C=C), 1440, 1153, 1047, 968; [Anal. Calcd. for $C_{17}H_{11}Cl_2NO$: C, 64.58; H, 3.51; N, 4.43; Found: C, 64.59; H, 3.53; N, 4.42]; LC/MS (ESI, *m/z*): 315.10 [M+] for 315.02 $C_{17}H_{11}Cl_2NO$.

(*E*)-1-(1-*H*-Indol-3-yl)-3-(4-methoxyphenyl)prop-2-en-1-one (**1e**)

Yellow powder; m.p: 168-172 °C; ^{1}H-NMR (400 MHz, DMSO-d_6) δ: 3.37 (s, 3H, OCH_3), 7.01 (d, 2H, J = 8.8 Hz, Ar-H), 7.02 – 7.24 (m, 2H, Ar-H), 7.49 (d, 1H, J = 7.32 Hz, COCH=CH), 7.59 (d, 1H, J = 16.2 Hz, Ar-H), 7.70 (d, 1H, J = 15.4 Hz, COCH=CH), 7.80(d, 2H, J = 8.8 Hz, Ar-H), 8.33 (d, 1H, J = 6.6 Hz, Ar-H), 8.69 (s, 1H, Ar-H), 12.06 (s, 1H, NH); ^{13}C-NMR (100 MHz, DMSO-d_6) δ: 55.3, 112.1, 114.3, 117.8, 121.8, 122.3, 123.0, 126, 127.8, 130.1, 134.4, 136.8, 139.4, 160.7, 183.7; IR (KBr, cm^{-1}) ν_{max}= 3100(NH), 2866(C-H), 2372, 1715(C=O), 1514(C=C), 1438, 1249, 1159, 976, 746; [Anal. Calcd. for $C_{18}H_{15}NO_2$: C, 77.96; H, 5.45; N, 5.05; Found: C, 77.90; H, 5.41; N, 5.11]; LC/MS (ESI, *m/z*): 277.10 [M+] for 277.11 $C_{18}H_{15}NO_2$.

(*E*)-3-(4-Bromophenyl)-1-(1-*H*-indol-3-yl)prop-2-en-1-one **(1f)**

Yellow powder; m.p: 235-238 °C; ^{1}H-NMR (400 MHz, DMSO-d_6) δ: 7.22 – 7.25 (m, 2H, Ar-H), 7.50 (d, 1H, J = 8.04 Hz, Ar-H), 7.59 (d, 1H, J = 15.4 Hz, COCH=CH), 7.65 (d, 2H, J = 8.8 Hz, Ar-H), 7.81-7.89 (m, 3H, Ar-H & COCH=CH), 8.33 (d, 1H, J = 7.36 Hz, Ar-H), 8.75 (s, 1H, Ar-H), 12.13 (s, 1H, NH);^{13}C-NMR (100 MHz, DMSO-d_6) δ: 112.2, 117.7, 121.6, 121.8, 123.0, 125.5, 125.8, 130.2, 131.8, 134.5, 134.6, 135.1, 136.9, 138.2, 183.3; IR (KBr, cm^{-1}) ν_{max}= 3215(NH), 2929(C-H), 1643(C=O), 1576(C=C), 1517, 1443,

1144, 970, 734; [Anal.Calcd. for $C_{17}H_{12}BrNO$: C,62.60;H,3.71; N, 4.21; Found: C,62.65;H,3.81; N, 4.19]; LC/MS (ESI, *m/z*): 325.10[M+] for 325.01 $C_{17}H_{12}BrNO$.

(*E*)-3-(4-Fluorophenyl)-1-(1-*H*-indol-3-yl)prop-2-en-1-one (**1g)**

Yield (84%); Yellow powder; m.p: 204-206°C; ^{1}H-NMR (400 MHz, DMSO-d_6)δ: 7.22 – 7.29 (m, 3H, Ar-H), 7.51(d, 1H, J = 8.8 Hz, Ar-H), 7.64 (t, 1H, J = 15 Hz, COCH=CH), 7.78 – 7.83 (m, 2H, Ar-H & COCH=CH), 7.91 – 7.94 (m 2H, Ar-H), 8.35 (d, 1H, J = 7.32 Hz, Ar-H), 8.74 (s, 1H, Ar-H), 12.13 (s, 1H, NH); ^{13}C-NMR (100 MHz, DMSO-d_6) δ: 112.2, 114.7, 115.7, 116.0, 117.7, 121.8, 121.9, 123.2, 124.6, 125.9, 130.6, 130.7, 131.9, 134.8, 136.9, 138.3, 161.5, 164.4, 183.6; IR (KBr, cm^{-1}) ν_{max}= 3409(NH), 2867(C-H), 2979, 1641(C=O), 1514(C=C), 1439, 1237, 1009, 774, 507; [Anal. Calcd. for $C_{17}H_{12}FNO$: C, 76.97; H, 4.56; N, 5.28; Found: C, 76.98; H, 4.44; N, 5.32]; LC/MS (ESI, *m/z*): 265.09[M+] for 265.10 $C_{17}H_{12}FNO$.

(*E*)-3-(3-Fluorophenyl)-1-(1-*H*-indol-3-yl)prop-2-en-1-one (**1h)**

Yellow powder; m.p: 224-226°C; ^{1}H-NMR (400 MHz, DMSO-d_6) δ: 7.20 – 7.38 (m, 3H, Ar-H), 7.42 – 7.56 (m, 1H, COCH=CH), 7.60 – 7.72 (m, 3H, Ar-H & COCHCH), 7.81 (d, 1H, J = 3.2 Hz, Ar-H), 7.91 (d, 1H, J = 2.4 Hz, Ar-H), 8.35 (s, 1H, Ar-H), 8.75 (s, 1H, Ar-H), 12.15 (s, 1H, NH); ^{13}C-NMR (100 MHz, DMSO-d_6) δ: 112.2, 114.0, 114.2, 115.7, 115.9, 116.3, 116.5, 117.8, 121.8, 121.9, 123.2, 125.2, 125.8, 126.0, 130.7, 135.1, 136.9, 138.1, 161.4, 163.8, 183.3; IR (KBr, cm^{-1}) ν_{max}= 3433(NH), 3047(C-H), 2866, 1933, 1642(C=O), 1562(C=C), 1440, 1153, 1008, 879; [Anal. Calcd. for $C_{17}H_{12}FNO$: C, 76.97; H, 4.56; N, 5.28; Found: C, 76.80; H, 4.09; N, 5.25]; LC/MS (ESI, *m/z*): 265.10[M+] for 265.01 $C_{17}H_{12}FNO$.

(*E*)-1-(1-*H*-Indol-3-yl)-3-(*m*-tolyl)prop-2-en-1-one (**1i)**

Yellow powder; m.p: 197-199 °C; ^{1}H-NMR (400 MHz, DMSO-d_6) δ: 2.35 (s, 3H, CH_3), 7.18 – 7.25 (m, 2H, Ar-H), 7.31 (t, 1H, J = 8.04 Hz, Ar-H), 7.49 (d, 1H, J = 6.60 Hz, Ar-H), 7.57 (s, 1H, Ar-H), 7.61 (d, 2H, J = 5.8 Hz, Ar-H &COCH=CH), 7.67 (s, 1H,Ar-H), 7.81 (d, 1H, J = 15.4 Hz, COCH=CH), 8.33 (d, 1H, J = 8.8 Hz, Ar-H), 8.73 (d, 1H, J = 2.9 Hz, Ar-H), 12.10 (s, 1H, NH);^{13}C-NMR (100 MHz, DMSO-d_6) δ: 20.9, 112.1, 117.7, 121.7, 121.8, 123.0, 124.4, 125.7, 125.8, 128.6, 128.7, 130.5, 134.7, 135.1, 136.8, 138.0, 139.6, 183.6; IR (KBr, cm^{-1}) ν_{max}= 3411(NH), 2922(C-H), 1641(C=O), 1561(C=C),

1439, 1154, 1006, 746; [Anal. Calcd. for $C_{18}H_{15}NO$: C, 82.73; H, 5.79; N, 5.36; Found: C, 82.75; H, 5.91; N, 5.49]; LC/MS (ESI, *m/z*): 261.30[M+] for 261.32 $C_{18}H_{15}NO$.

(*E*)-3-(3-Bromophenyl)-1-(1-*H*-indol-3-yl)prop-2-en-1-one (**1j)**

Yellow powder; m.p: 228-230°C; ^{1}H-NMR (400 MHz, DMSO-d_6) δ: 7.19 – 7.26 (m, 2H, Ar-H), 7.39 (t, 1H, *J* = 8.08 Hz, Ar-H), 7.50 (d, 1H, *J* = 8.08 Hz, Ar-H), 7.57 – 7.60 (m, 2H, Ar-H & COCH=CH), 7.80 (d, 1H, *J* = 8.08 Hz, Ar-H), 7.89 (d, 1H, *J* = 15.4 Hz, COCH=CH), 8.16 (s, 1H, Ar-H), 8.31 (d, 1H, *J* = 6.60 Hz, Ar-H), 8.78 (s, 1H, Ar-H), 12.14 (s, 1H, NH); ^{13}C-NMR (100 MHz, DMSO-d_6) δ: 112.2, 117.7, 121.7, 121.9, 122.3, 123.1, 125.8, 126.1, 127.8, 130.2, 130.8, 132.2, 135.1, 136.8, 137.7, 137.8, 183.2; IR (KBr, cm^{-1}) ν_{max}= 3386(NH), 3248(C-H), 2958, 2867, 1716, 1618(C=O),1520(C=C), 1470,1422, 1243, 1137, 1153, 748, 698; [Anal. Calcd. for $C_{17}H_{12}BrNO$: C, 62.60; H, 3.71; N, 4.29; Found: C, 62.53; H, 3.70; N, 4.30]; LC/MS (ESI, *m/z*): 325.1[M+] for 325.01 $C_{17}H_{12}BrNO$.

(*E*)-1-(1-*H*-Indol-3-yl)-3-(4-(trifluoromethyl)phenyl)prop-2-en-1-one (**1k)**

Yellow powder; m.p: 240-242 °C; ^{1}H-NMR (400 MHz, DMSO-d_6)δ: 7.23 (t, 2H, *J* = 5.88 Hz, Ar-H), 7.50 (d, 1H, *J* = 6.6 Hz, Ar-H), 6.68 (d, 1H, *J* = 15.4 Hz, COCH=CH), 7.7 (d, 2H, *J* = 8.04 Hz, Ar-H), 7.97 (d, 1H, *J* = 15.4 Hz, COCH=CH), 8.06 (d, 2H, *J* = 7.36 Hz, Ar-H), 8.33 (d, 1H, *J* = 6.96 Hz, Ar-H), 8.78 (s, 1H, Ar-H), 12.16 (s, 1H, NH); ^{13}C-NMR (100 MHz, DMSO-d_6) δ: 112.2, 117.7, 121.7, 121.9, 123.2, 125.5, 125.8, 127.3, 128.9, 129.6, 135.2, 136.9,137.6, 139.5, 183.1; IR (KBr, cm^{-1}) ν_{max}= 3128(NH), 3044(C-H), 2870, 1644(C=O), 1519(C=C), 1439, 1336, 1154, 794, 753; [Anal. Calcd. for $C_{18}H_{12}F_3NO$: C, 68.57; H, 3.84; N, 4.44; Found: C, 68.75; H, 3.91; N, 4.49]; LC/MS (ESI, *m/z*): 315.10 [M+] for 315.0 $C_{18}H_{12}F_3NO$.

(*E*)-1-(1-*H*-Indol-3-yl)-3-(thiophen-2-yl)prop-2-en-1-one (**1l)**

Yellow powder; m.p: 209-211°C; ^{1}H-NMR (400 MHz, DMSO-d_6) δ: 7.14 – 7.25 (t, 1H, *J* = 4.40 Hz, Ar-H), 7.18 – 7.25 (m, 2H, Ar-H), 7.47 – 7.51 (m, 2H, Ar-H & COCH=CH), 7.58 (d, 1H, *J* = 3.64 Hz, Ar-H), 7.69 (d, 1H, *J* = 5.12 Hz, Ar-H), 7.79 (d, 1H, *J* = 15.4 Hz, COCH=CH), 8.32 (d, 1H, *J* = 6.6 Hz, Ar-H), 8.65 (s, 1H, Ar-H), 12.09 (s, 1H, NH); ^{13}C-NMR (100 MHz, DMSO-d_6) δ:112.1, 117.4, 121.7, 121.8, 123.0, 123.2, 125.8, 128.4, 128.8, 131.1, 132.4, 134.5, 136.8, 140.2, 183.1; IR (KBr, cm^{-1}) ν_{max}= 3432(NH), 3094(C-H), 2921, 1632(C=O), 1581(C=C), 1491, 1198, 428; [Anal. Calcd. for $C_{15}H_{11}NOS$: C,

71.12; H, 4.38; N, 5.53; Found: C, 71.21; H, 4.43; N, 5.49]; LC/MS (ESI, *m/z*): 253.10 [M+] for 253.06 $C_{15}H_{11}NOS$.

(*E*)-3-(Furan-2-yl)-1-(1-*H*-indol-3-yl)prop-2-en-1-one (**1m)**

Yellow powder; m.p: 157-159 °C; ^{1}H-NMR (400 MHz, DMSO-d_6) δ: 6.64 (t, 1H, *J* = 5.12 Hz, Ar-H), 6.97 (d, 1H, *J* = 3.64 Hz, Ar-H), 7.18 – 7.23 (m, 2H, Ar-H), 7.44 – 7.53 (m, 3H, Ar-H & COCH=CH), 7.84 (s, 1H, Ar-H), 8.32 (d, 1H, *J* = 16.12 Hz, COCH=CH), 8.61 (d, 1H, *J* = 2.96 Hz, Ar-H), 12.08 (s, 1H, NH); ^{13}C-NMR (100 MHz, DMSO-d_6) δ: 112.1, 112.7, 114.8, 117.5, 121.7, 121.75, 121.8, 123.1, 125.8, 126.6, 134.3, 136.8, 145.1, 151.5, 183.1; IR (KBr, cm^{-1}) ν_{max}= 3434(NH), 3041(C-H), 2917, 1621(C=O), 1548(C=C), 1428, 1155, 771, 452; [Anal. Calcd. for $C_{15}H_{11}NO_2$: C, 75.94; H, 4.67; N, 5.96; Found: C, 75.90; H, 4.91; N, 5.49]; LC/MS (ESI, *m/z*): 237.10[M+] for 237.08 $C_{15}H_{11}NO_2$.

(*E*)-1-(1-*H*-Indol-3-yl)-3-(3,4,5-trimethoxyphenyl)prop-2-en-1-one (**1n)**

Yellow powder; m.p: 217-219 °C; ^{1}H-NMR (400 MHz, DMSO-d_6) δ: 3.68 (s, 3H, OCH$_3$), 3.87 (s, 6H, 2xOCH$_3$), 7.17 (s, 3H, Ar-H), 7.22 (t, 1H, *J* = 7.32 Hz, Ar-H), 7.50 (d, 1H, *J* = 7.32 Hz, Ar-H), 7.58 (d, 1H, *J* = 15.4 Hz, COCH=CH), 7.74 (d, 1H, *J* = 15.4 Hz, COCH=CH), 8.34 (d, 1H, *J* = 7.32 Hz, Ar-H), 8.73 (s, 1H, Ar-H), 12.10 (s, 1H, NH); ^{13}C-NMR (100 MHz, DMSO-d_6) δ: 56.1, 60.1, 106.0, 112.2, 121.7, 121.7, 123.0, 123.8, 125.8, 130.7, 134.6, 139.0, 139.9, 153.0, 183.2; IR (KBr, cm^{-1}) ν_{max}= 3214(NH), 3115(C-H), 2830, 1641(C=O), 1582(C=C), 1506, 1421, 1266, 1185, 1128, 969, 575; [Anal. Calcd. for $C_{20}H_{19}NO_4$: C, 71.20; H, 5.68; N, 4.15; Found: C, 71.77; H, 5.51; N, 4.44]; LC/MS (ESI, *m/z*): 337.10[M+] for 337.13 $C_{20}H_{19}NO_4$.

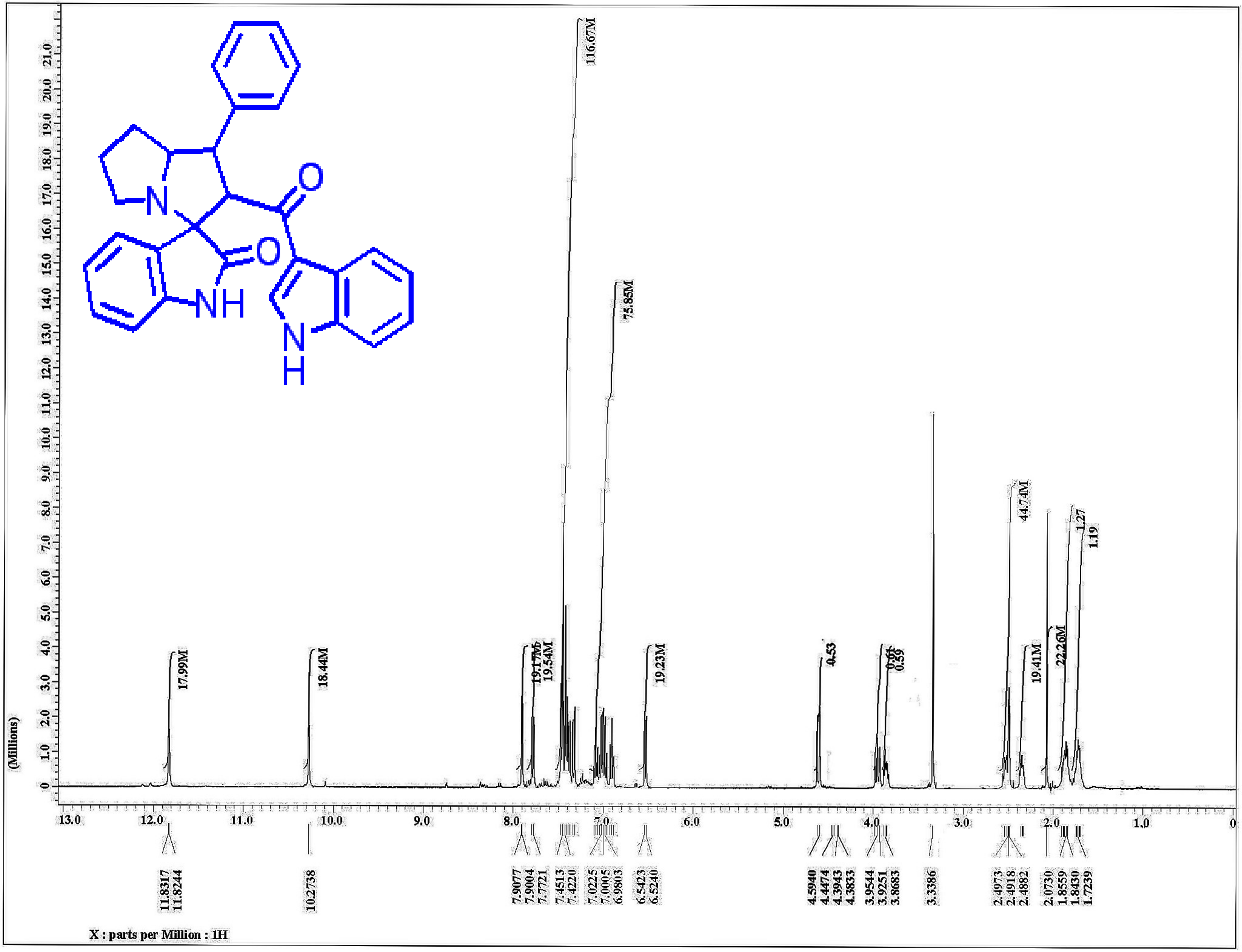

(Millions)
X : parts per Million : 1H
17.99M
18.44M
19.17M
19.54M
116.67M
75.85M
19.23M
0.53
0.61
0.59
44.74M
19.41M
22.26M
1.27
1.19
11.8317
11.8244
10.2738
7.9077
7.9004
7.7721
7.4513
7.4220
7.0225
7.0005
6.9803
6.5423
6.5240
4.5940
4.4474
4.3943
4.3833
3.9544
3.9251
3.8683
3.3386
2.4973
2.4918
2.4882
2.0730
1.8559
1.8430
1.7239

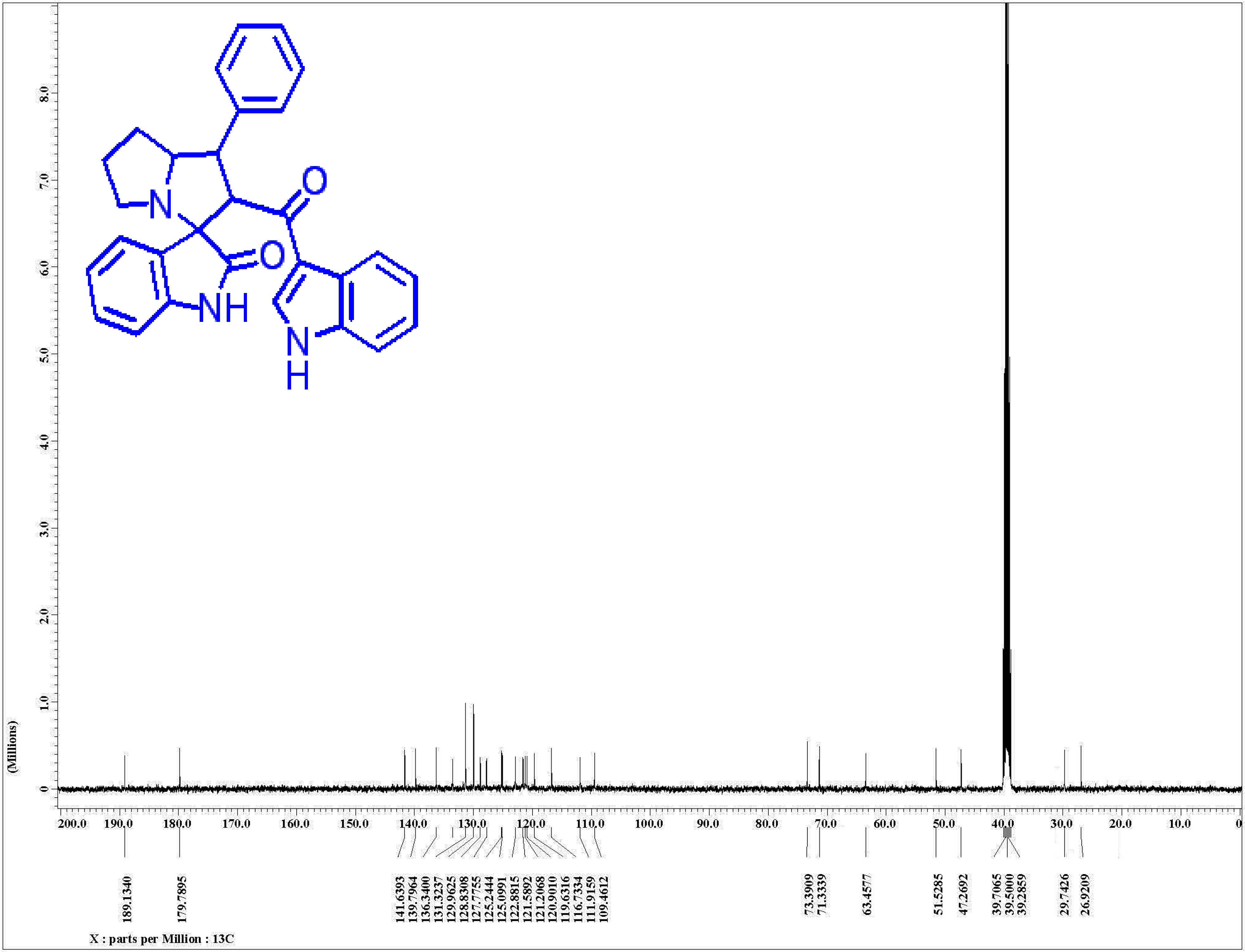

(Millions)
0
1.0
2.0
3.0
4.0
5.0
6.0
7.0
8.0
O
O
N
NH
N
H
200.0 190.0 180.0 170.0 160.0 150.0 140.0 130.0 120.0 110.0 100.0 90.0 80.0 70.0 60.0 50.0 40.0 30.0 20.0 10.0 0
189.1340
179.7895
141.6393
139.7964
136.3400
131.3237
129.9625
128.8308
127.7755
125.2444
125.0991
122.8815
121.5892
121.2068
120.9010
119.6316
116.7334
111.9159
109.4612
73.3909
71.3339
63.4577
51.5285
47.2692
39.7065
39.5000
39.2859
29.7426
26.9209
X : parts per Million : 13C

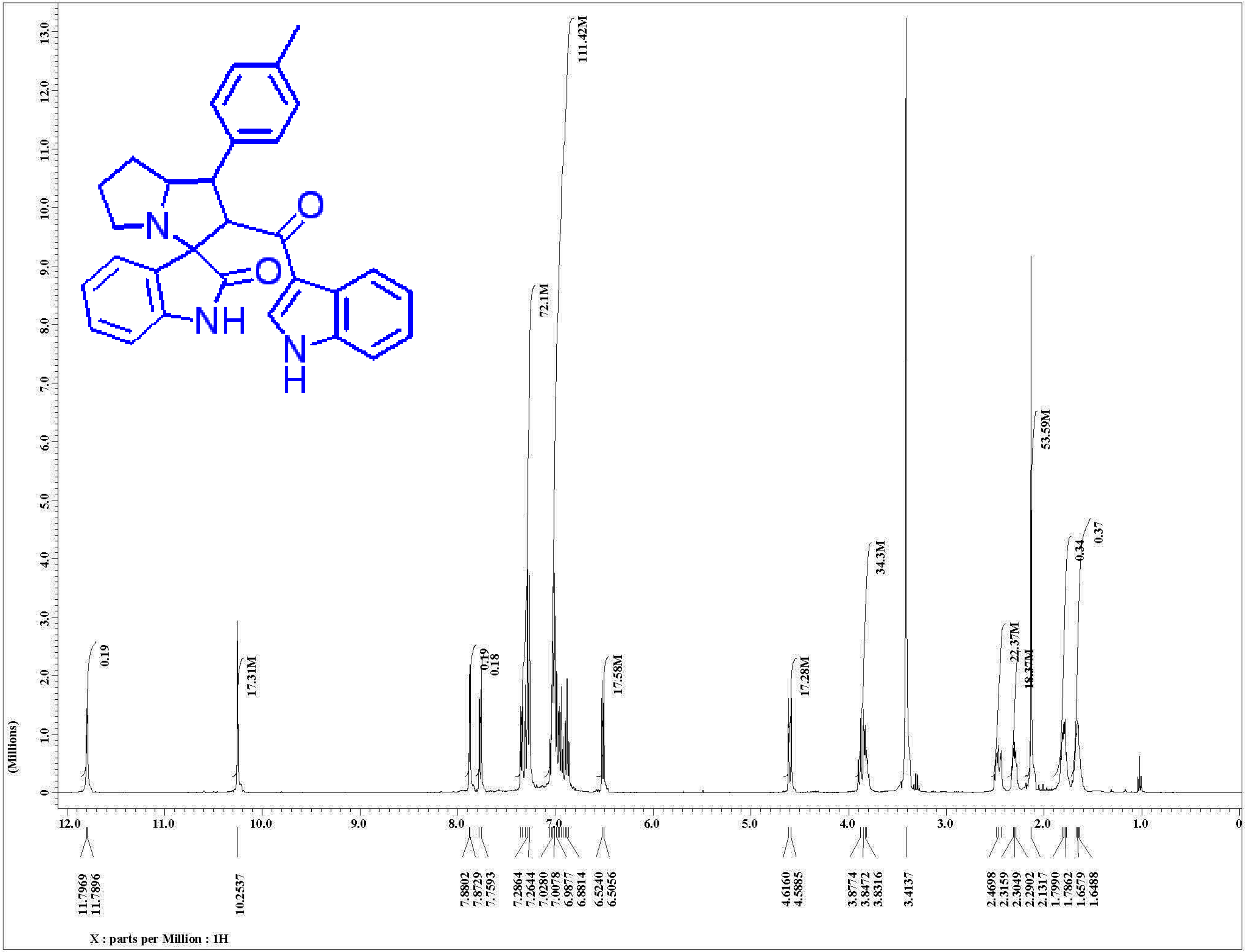

X : parts per Million : 1H
(Millions)
11.7969
11.7896
10.2537
7.8802
7.8729
7.7593
7.2864
7.2644
7.0280
7.0078
6.9877
6.8814
6.5240
6.5056
4.6160
4.5885
3.8774
3.8472
3.8316
3.4137
2.4698
2.3159
2.3049
2.2902
2.1317
1.7990
1.7862
1.6579
1.6488
0.19
17.31M
0.19
0.18
72.1M
111.42M
17.58M
17.28M
34.3M
22.37M
18.37M
53.59M
0.34
0.37

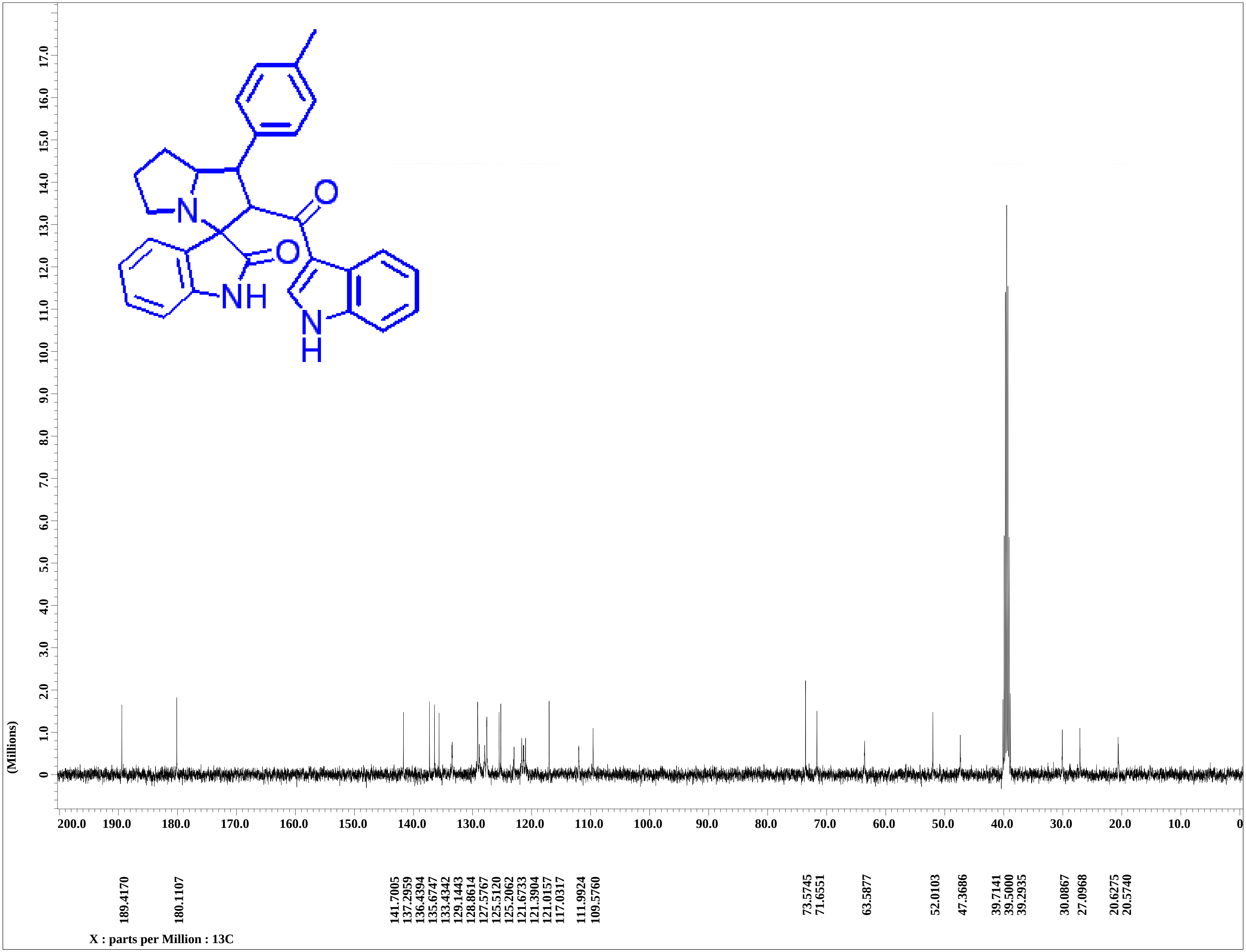
(Millions)
X : parts per Million : 13C
189.4170
180.1107
141.7005
137.2959
136.4394
135.6747
133.4342
129.1443
128.8614
127.5767
125.5120
125.2062
121.6733
121.3904
121.0157
117.0317
111.9924
109.5760
73.5745
71.6551
63.5877
52.0103
47.3686
39.7141
39.5000
39.2935
30.0867
27.0968
20.6275
20.5740
NH
N
H
O
O
N

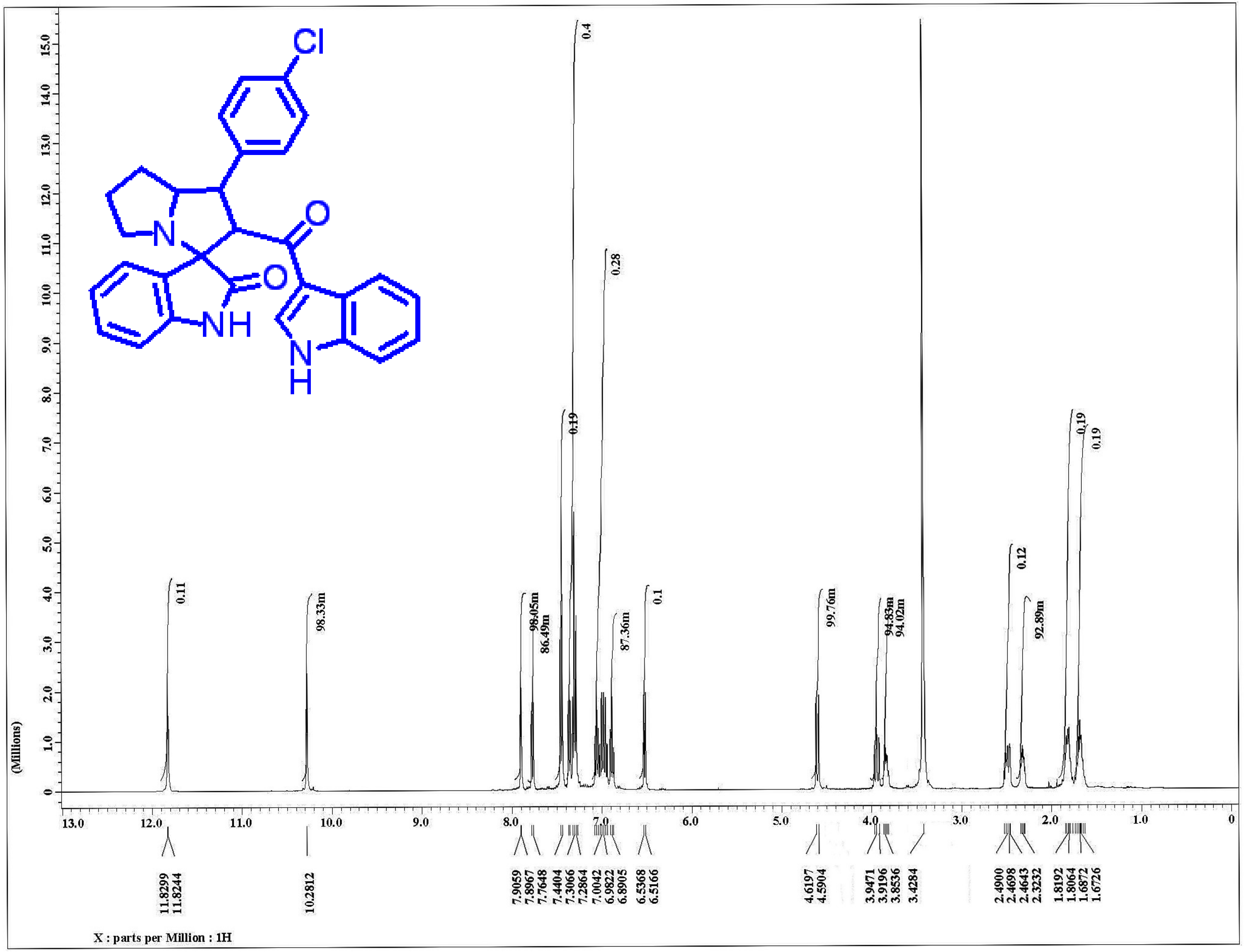
Cl
O
O
N
NH
N
H
(Millions)
0.11
98.33m
98.05m
86.49m
0.19
0.4
0.28
87.36m
0.1
99.76m
94.83m
94.02m
0.12
92.89m
0.19
0.19
11.8299
11.8244
10.2812
7.9059
7.8967
7.7648
7.4404
7.3066
7.2864
7.0042
6.9822
6.8905
6.5368
6.5166
4.6197
4.5904
3.9471
3.9196
3.8536
3.4284
2.4900
2.4698
2.4643
2.3232
1.8192
1.8064
1.6872
1.6726
X : parts per Million : 1H

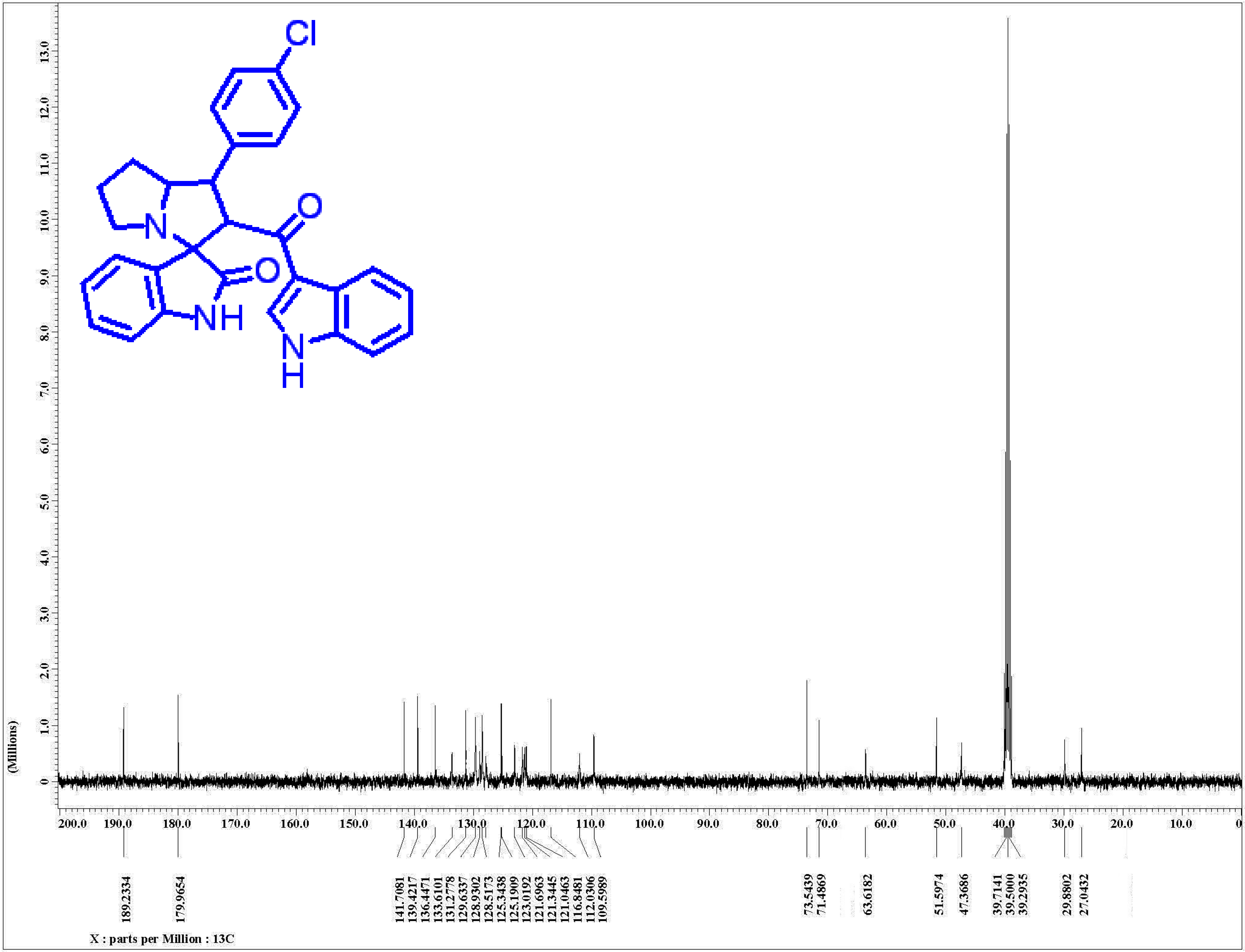

Cl
N
O
O
NH
N
H
(Millions)
0
1.0
2.0
3.0
4.0
5.0
6.0
7.0
8.0
9.0
10.0
11.0
12.0
13.0
200.0 190.0 180.0 170.0 160.0 150.0 140.0 130.0 120.0 110.0 100.0 90.0 80.0 70.0 60.0 50.0 40.0 30.0 20.0 10.0 0
189.2334
179.9654
141.7081
139.4217
136.4471
133.6101
131.2778
129.6337
128.9302
128.5173
125.3438
125.1909
123.0192
121.6963
121.3445
121.0463
116.8481
112.0306
109.5989
73.5439
71.4869
63.6182
51.5974
47.3686
39.7141
39.5000
39.2935
29.8802
27.0432
X : parts per Million : 13C

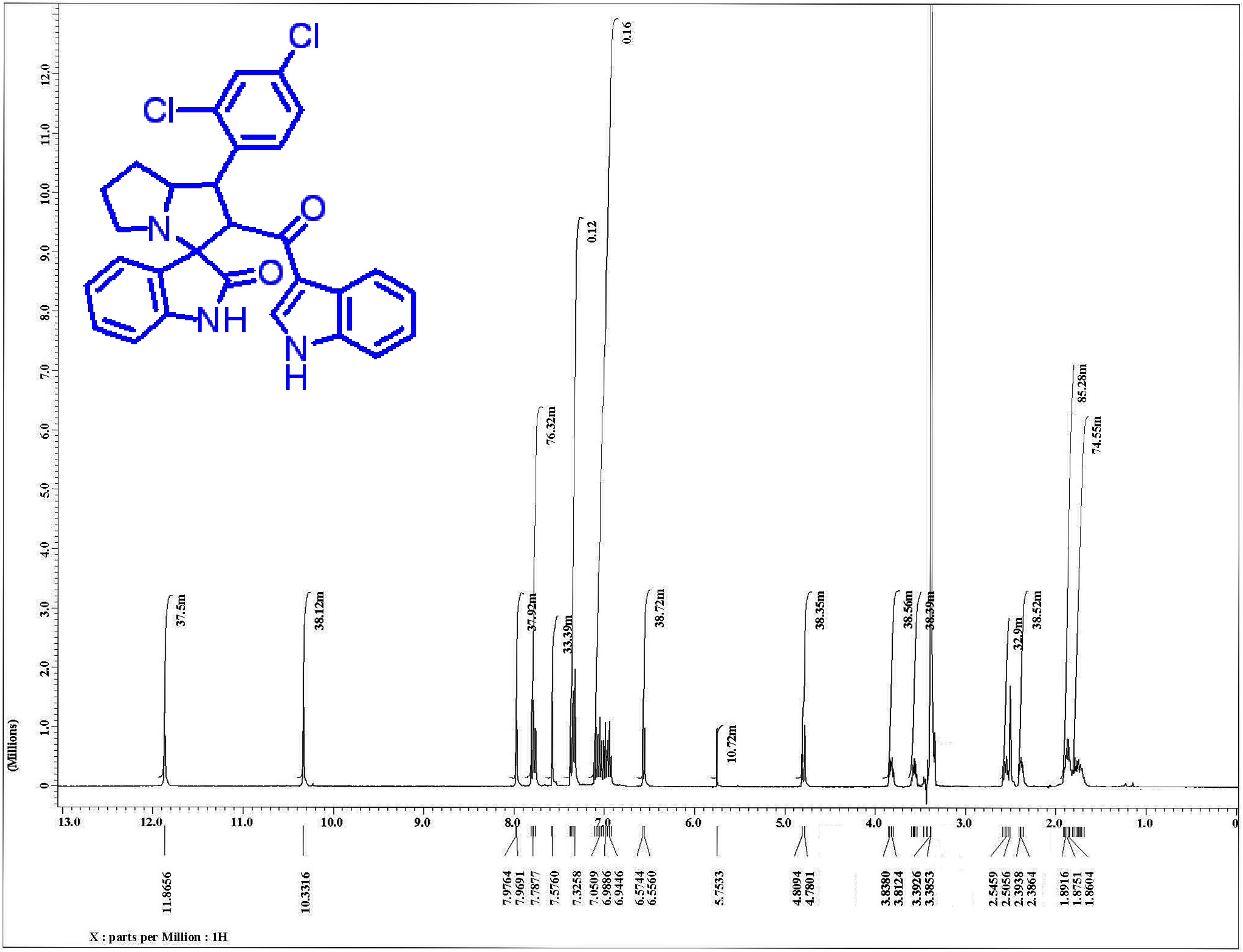

Cl
Cl
N
NH
O
O
N
H
37.5m
38.12m
37.92m
76.32m
33.39m
0.12
0.16
38.72m
10.72m
38.35m
38.56m
38.39m
32.9m
38.52m
85.28m
74.55m
11.8656
10.3316
7.9764
7.9691
7.7877
7.5760
7.3258
7.0509
6.9886
6.9446
6.5744
6.5560
5.7533
4.8094
4.7801
3.8380
3.8124
3.3926
3.3853
2.5459
2.5056
2.3938
2.3864
1.8916
1.8751
1.8604
(Millions)
X : parts per Million : 1H

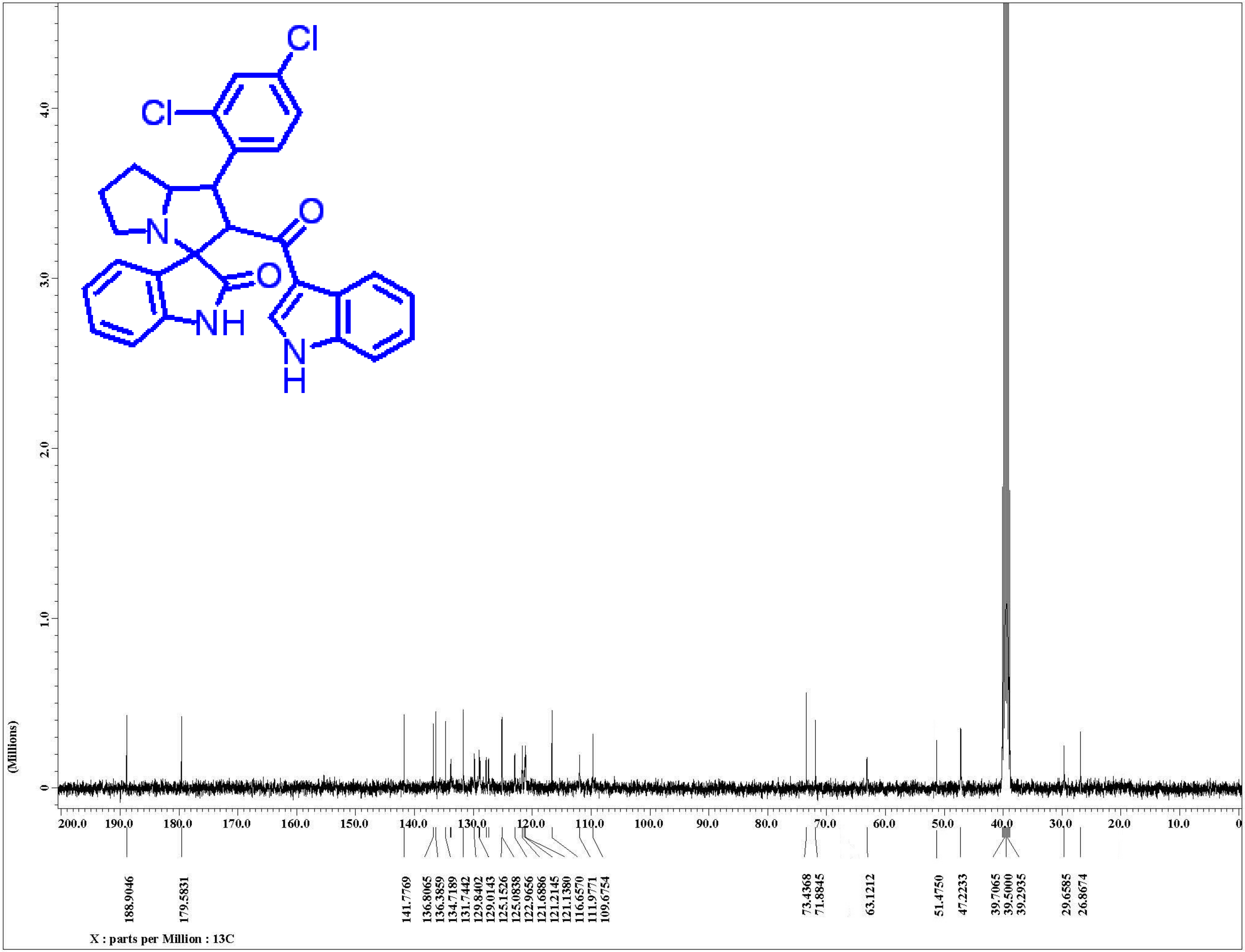
Cl
Cl
N
O
O
NH
N
H
(Millions)
4.0
3.0
2.0
1.0
0
200.0 190.0 180.0 170.0 160.0 150.0 140.0 130.0 120.0 110.0 100.0 90.0 80.0 70.0 60.0 50.0 40.0 30.0 20.0 10.0 0
188.9046
179.5831
141.7769
136.8065
136.3859
134.7189
131.7442
129.8402
129.0143
125.1526
125.0838
122.9656
121.6886
121.2145
121.1380
116.6570
111.9771
109.6754
73.4368
71.8845
63.1212
51.4750
47.2233
39.7065
39.5000
39.2935
29.6585
26.8674
X : parts per Million : 13C

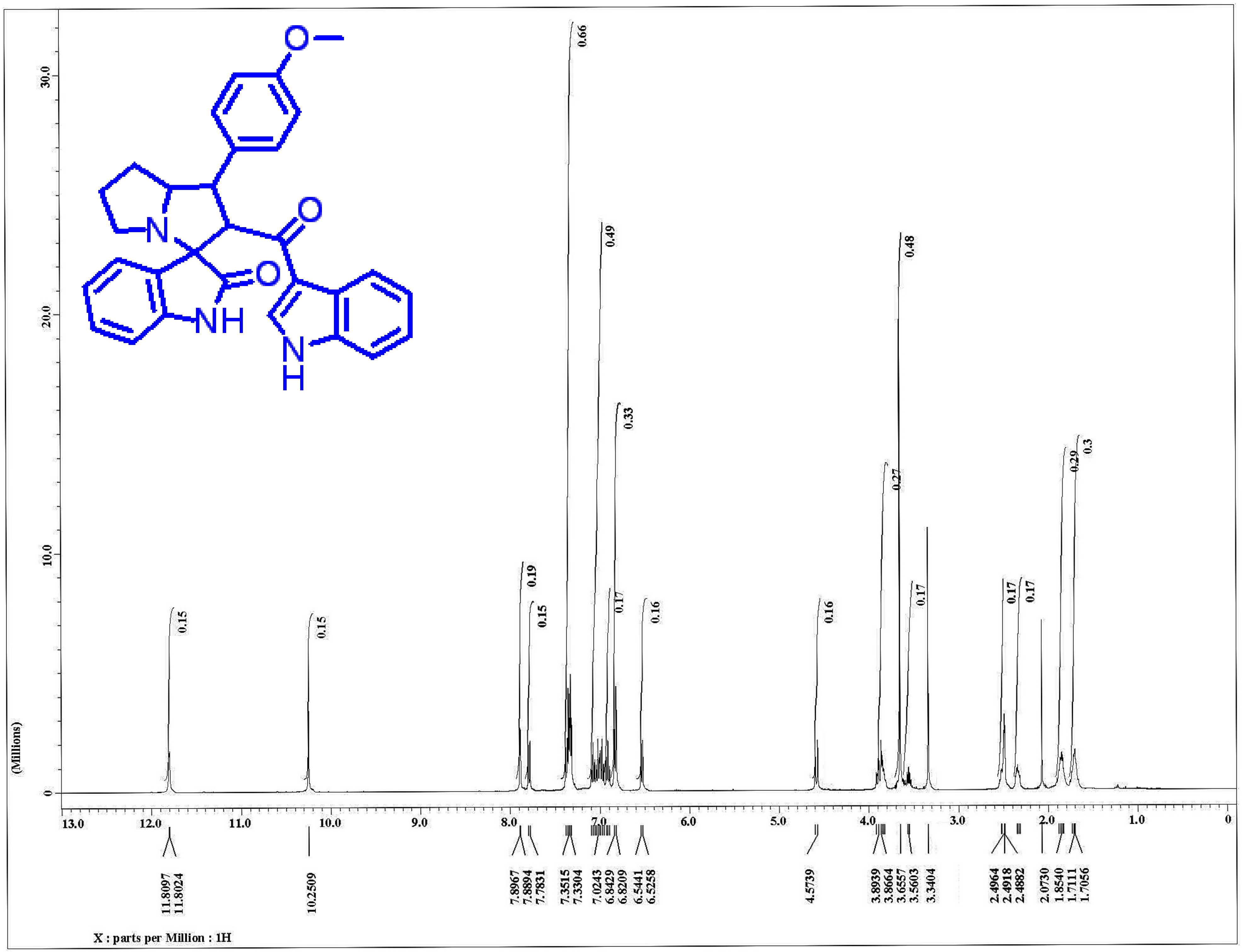

(Millions)
30.0
20.0
10.0
0
0.66
0.49
0.48
0.33
0.27
0.29
0.3
0.19
0.15
0.15
0.15
0.17
0.16
0.16
0.17
0.17
0.17
13.0
12.0
11.0
10.0
9.0
8.0
7.0
6.0
5.0
4.0
3.0
2.0
1.0
0
11.8097
11.8024
10.2509
7.8967
7.8894
7.7831
7.3515
7.3304
7.0243
6.8429
6.8209
6.5441
6.5258
4.5739
3.8939
3.8664
3.6557
3.5603
3.3404
2.4964
2.4918
2.4882
2.0730
1.8540
1.7111
1.7056
X : parts per Million : 1H

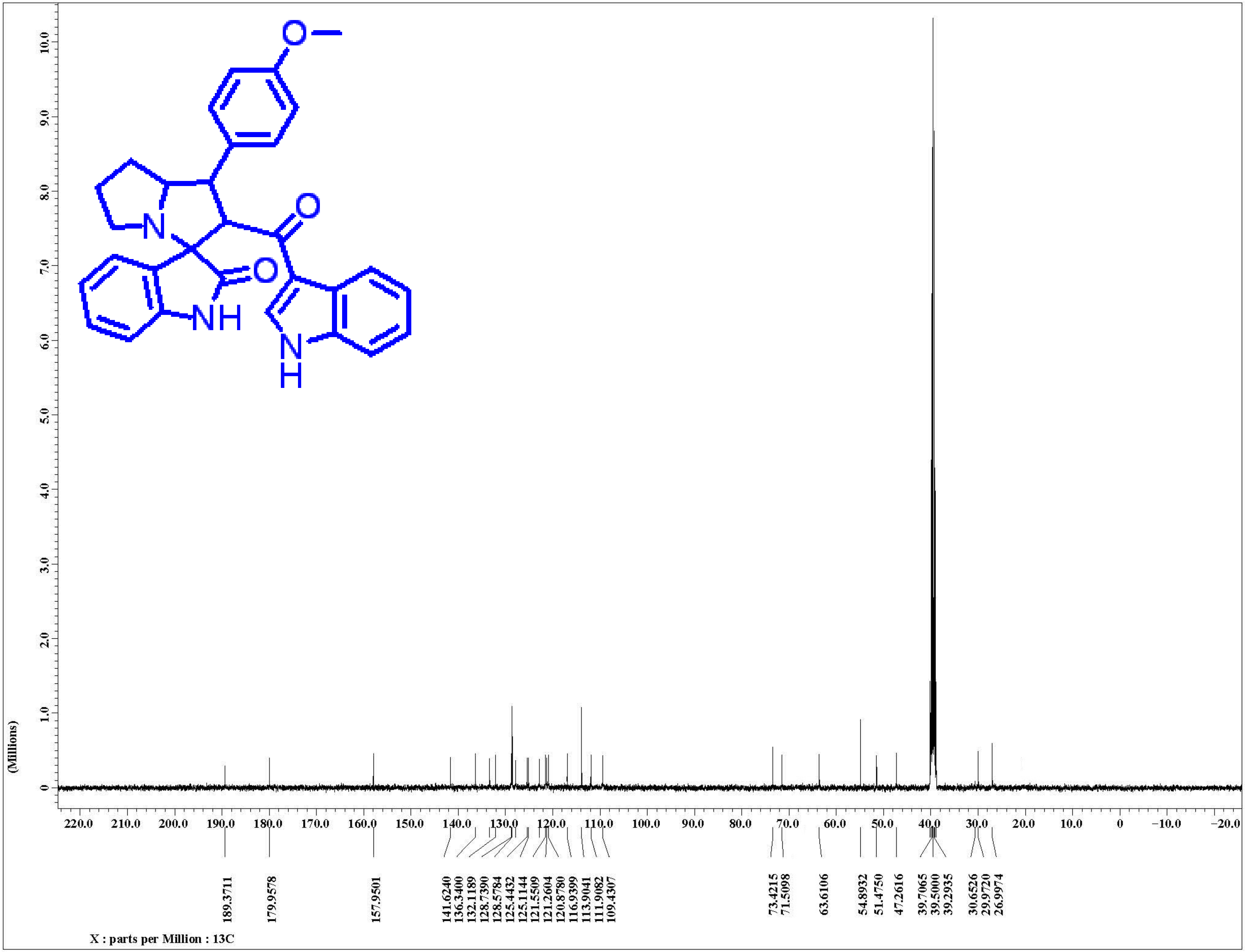

O
O
O
N
NH
N
H
(Millions)
10.0
9.0
8.0
7.0
6.0
5.0
4.0
3.0
2.0
1.0
0
220.0
210.0
200.0
190.0
180.0
170.0
160.0
150.0
140.0
130.0
120.0
110.0
100.0
90.0
80.0
70.0
60.0
50.0
40.0
30.0
20.0
10.0
0
−10.0
−20.0
189.3711
179.9578
157.9501
141.6240
136.3400
132.1189
128.7390
128.5784
125.4432
125.1144
121.5509
121.2604
120.8780
116.9399
113.9041
111.9082
109.4307
73.4215
71.5098
63.6106
54.8932
51.4750
47.2616
39.7065
39.5000
39.2935
30.6526
29.9720
26.9974
X : parts per Million : 13C

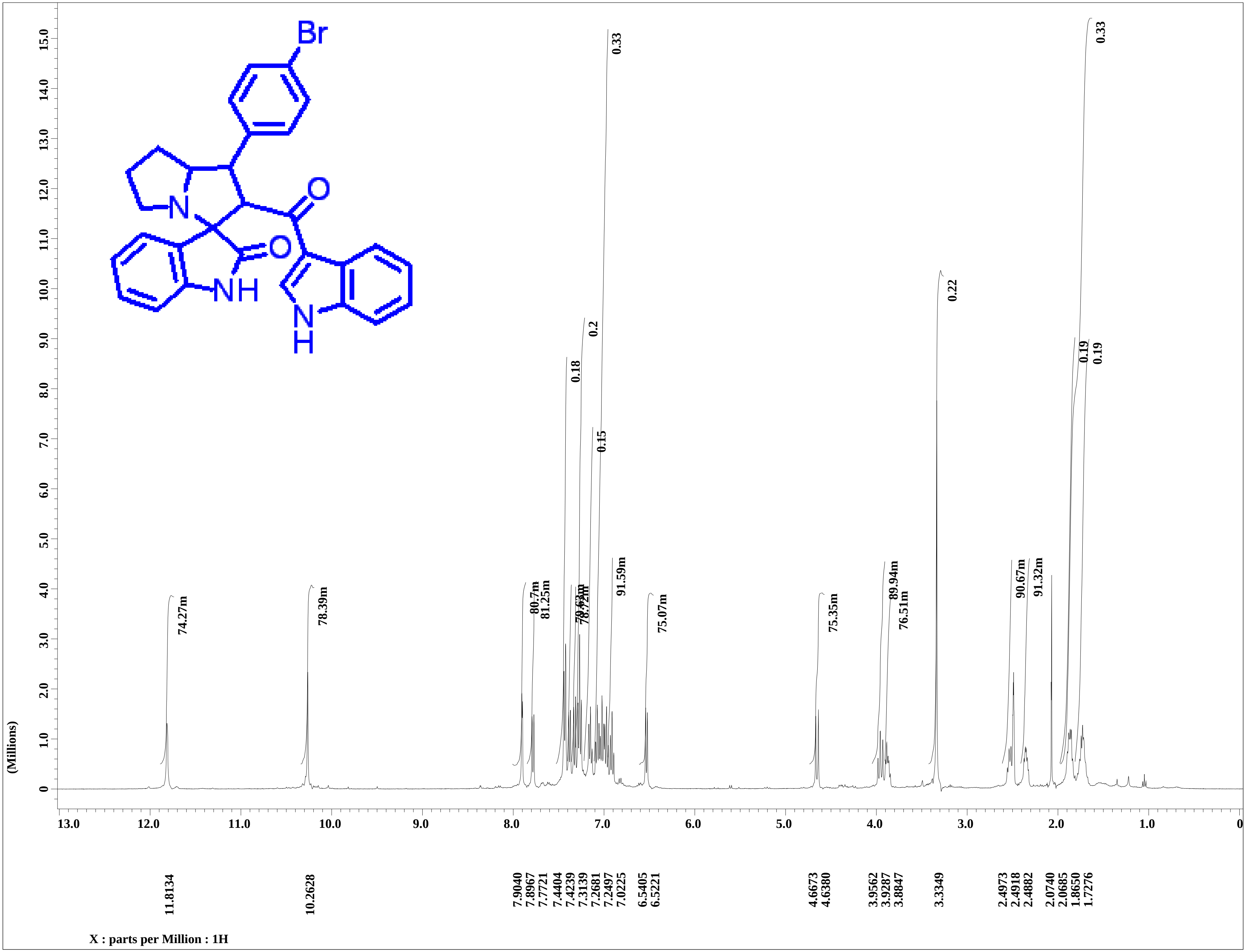

Br
O
N
O
NH
N
H
(Millions)
0.33
0.2
0.18
0.15
0.22
0.19
0.19
74.27m
78.39m
80.7m
81.25m
79.63m
78.72m
91.59m
75.07m
75.35m
89.94m
76.51m
90.67m
91.32m
11.8134
10.2628
7.9040
7.8967
7.7721
7.4404
7.4239
7.3139
7.2681
7.2497
7.0225
6.5405
6.5221
4.6673
4.6380
3.9562
3.9287
3.8847
3.3349
2.4973
2.4918
2.4882
2.0740
2.0685
1.8650
1.7276
X : parts per Million : 1H

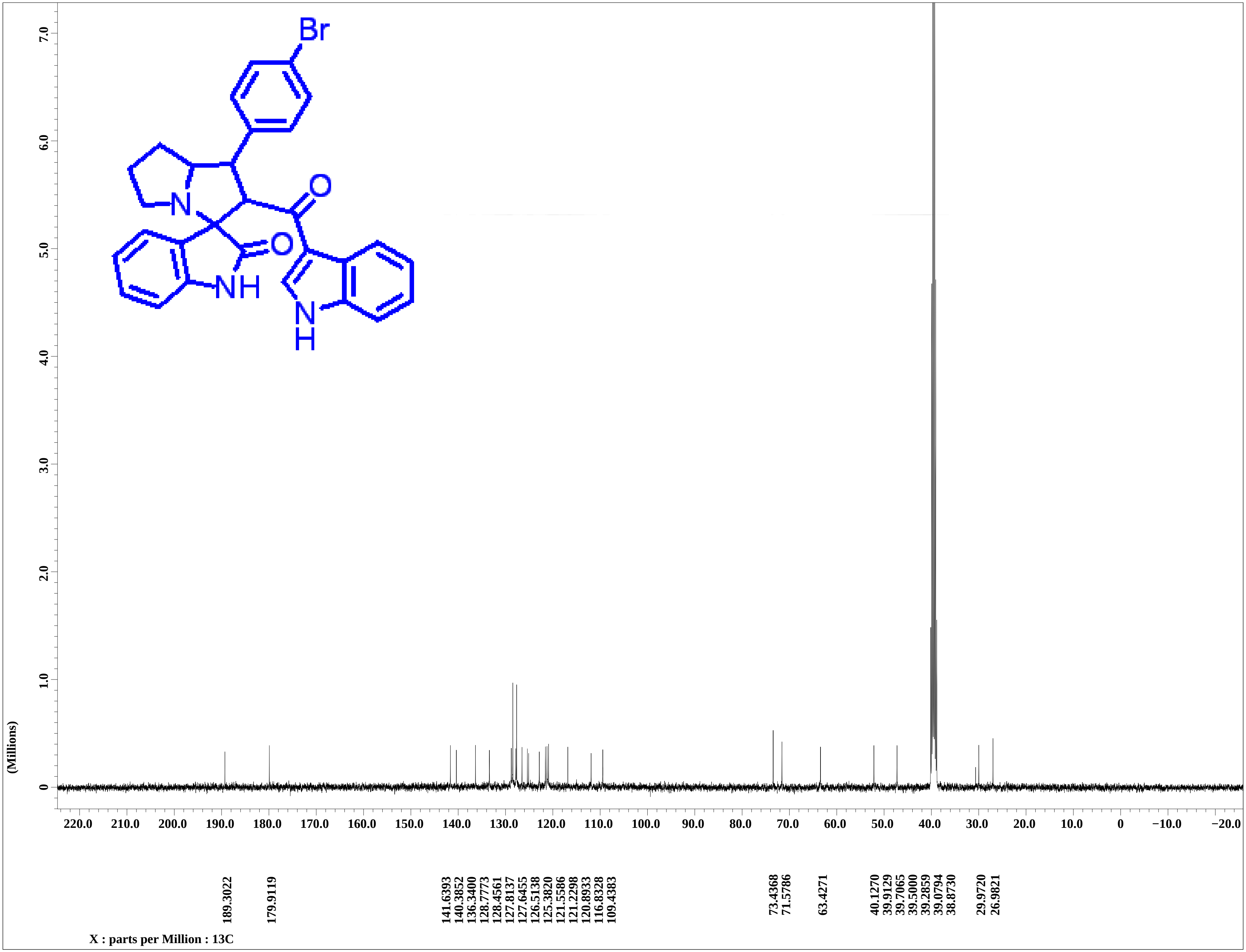

7.0
6.0
5.0
4.0
3.0
2.0
1.0
0
(Millions)
220.0 210.0 200.0 190.0 180.0 170.0 160.0 150.0 140.0 130.0 120.0 110.0 100.0 90.0 80.0 70.0 60.0 50.0 40.0 30.0 20.0 10.0 0 −10.0 −20.0
189.3022
179.9119
141.6393
140.3852
136.3400
128.7773
128.4561
127.8137
127.6455
126.5138
125.3820
121.5586
121.2298
120.8933
116.8328
109.4383
73.4368
71.5786
63.4271
40.1270
39.9129
39.7065
39.5000
39.2859
39.0794
38.8730
29.9720
26.9821
X : parts per Million : 13C
Br
O
N
O
NH
N
H

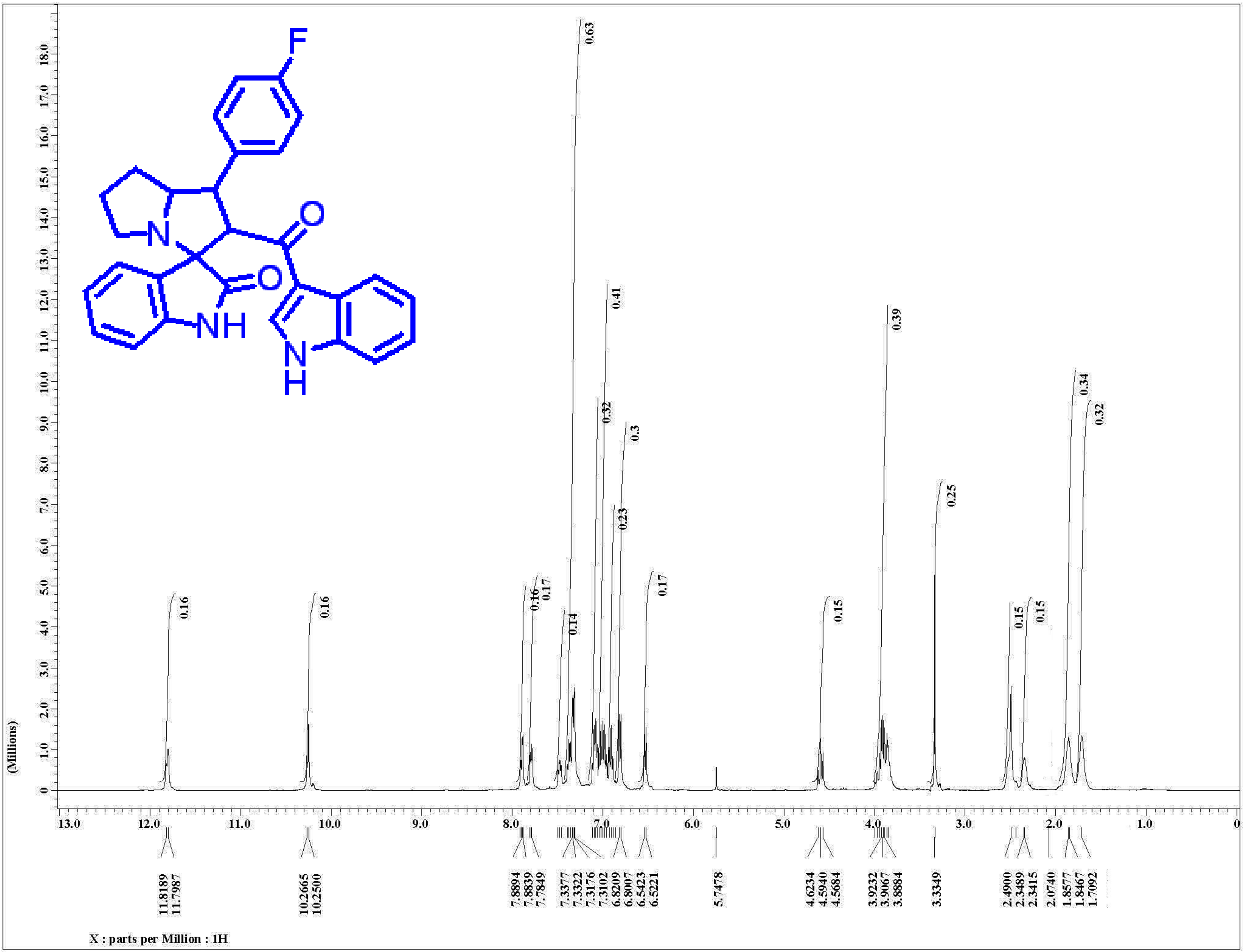

F
O
O
N
NH
N
H
0.63
0.41
0.39
0.34
0.32
0.32
0.3
0.25
0.23
0.17
0.17
0.16
0.16
0.16
0.15
0.15
0.15
0.14
11.8189
11.7987
10.2665
10.2500
7.8894
7.8839
7.7849
7.3377
7.3322
7.3176
7.3102
6.8209
6.8007
6.5423
6.5221
5.7478
4.6234
4.5940
4.5684
3.9232
3.9067
3.8884
3.3349
2.4900
2.3489
2.3415
2.0740
1.8577
1.8467
1.7092
(Millions)
X : parts per Million : 1H

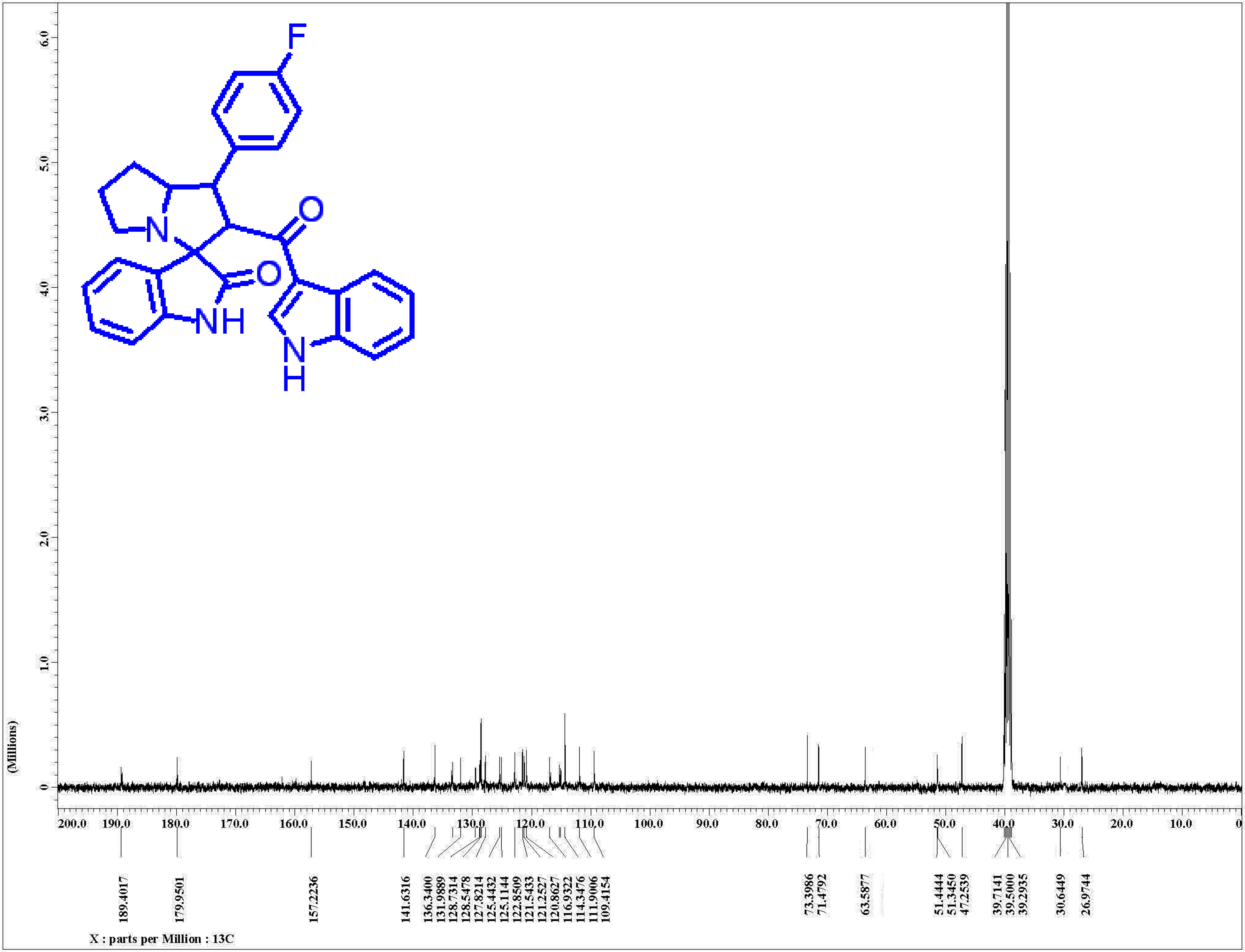

F
O
O
N
NH
N
H
(Millions)
6.0
5.0
4.0
3.0
2.0
1.0
0
200.0
190.0
180.0
170.0
160.0
150.0
140.0
130.0
120.0
110.0
100.0
90.0
80.0
70.0
60.0
50.0
40.0
30.0
20.0
10.0
0
189.4017
179.9501
157.2236
141.6316
136.3400
131.9889
128.7314
128.5478
127.8214
125.4432
125.1144
122.8509
121.5433
121.2527
120.8627
116.9322
114.3476
111.9006
109.4154
73.3986
71.4792
63.5877
51.4444
51.3450
47.2539
39.7141
39.5000
39.2935
30.6449
26.9744
X : parts per Million : 13C

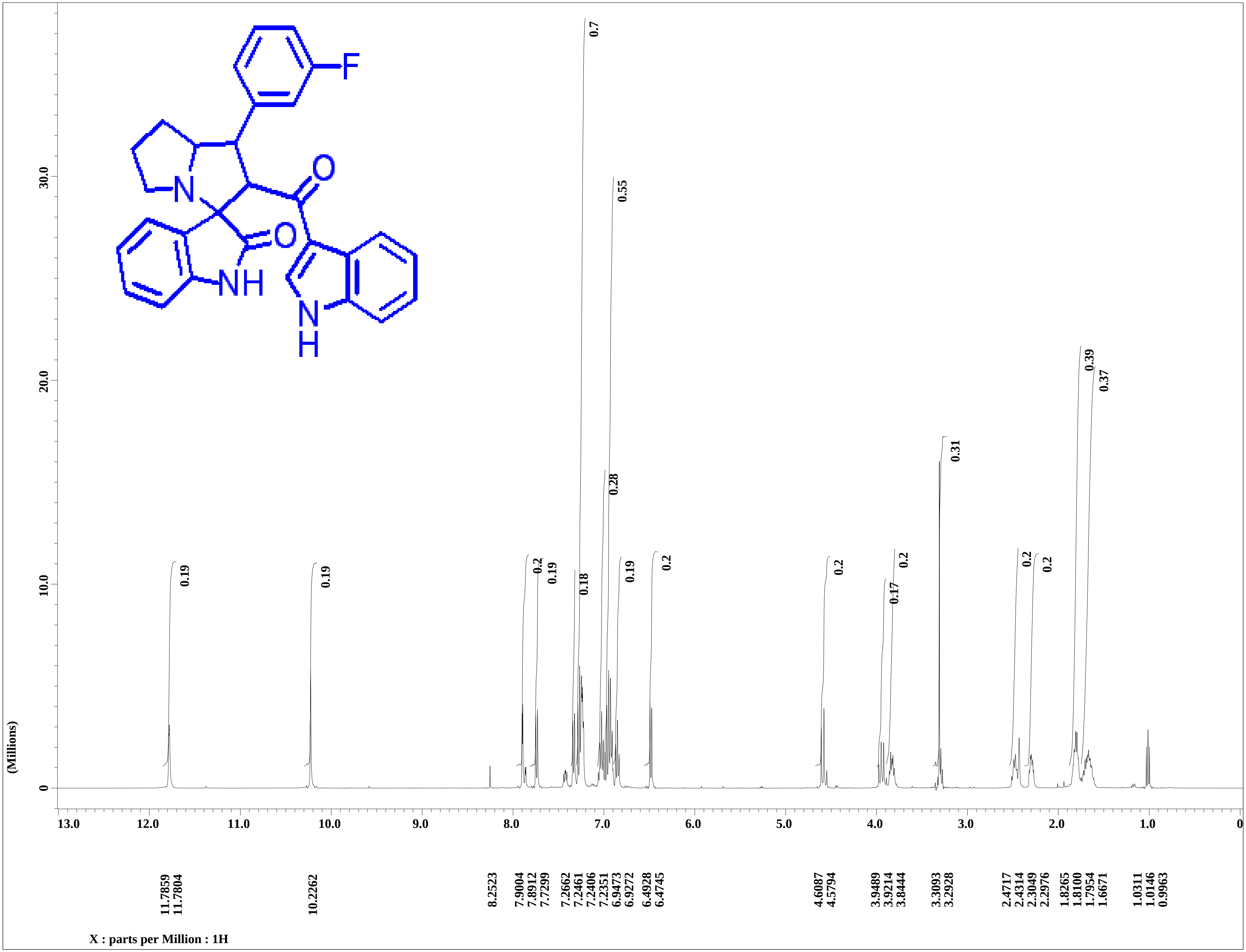

(Millions)
X : parts per Million : 1H
11.7859
11.7804
10.2262
8.2523
7.9004
7.8912
7.7299
7.2662
7.2461
7.2406
7.2351
6.9473
6.9272
6.4928
6.4745
4.6087
4.5794
3.9489
3.9214
3.8444
3.3093
3.2928
2.4717
2.4314
2.3049
2.2976
1.8265
1.8100
1.7954
1.6671
1.0311
1.0146
0.9963

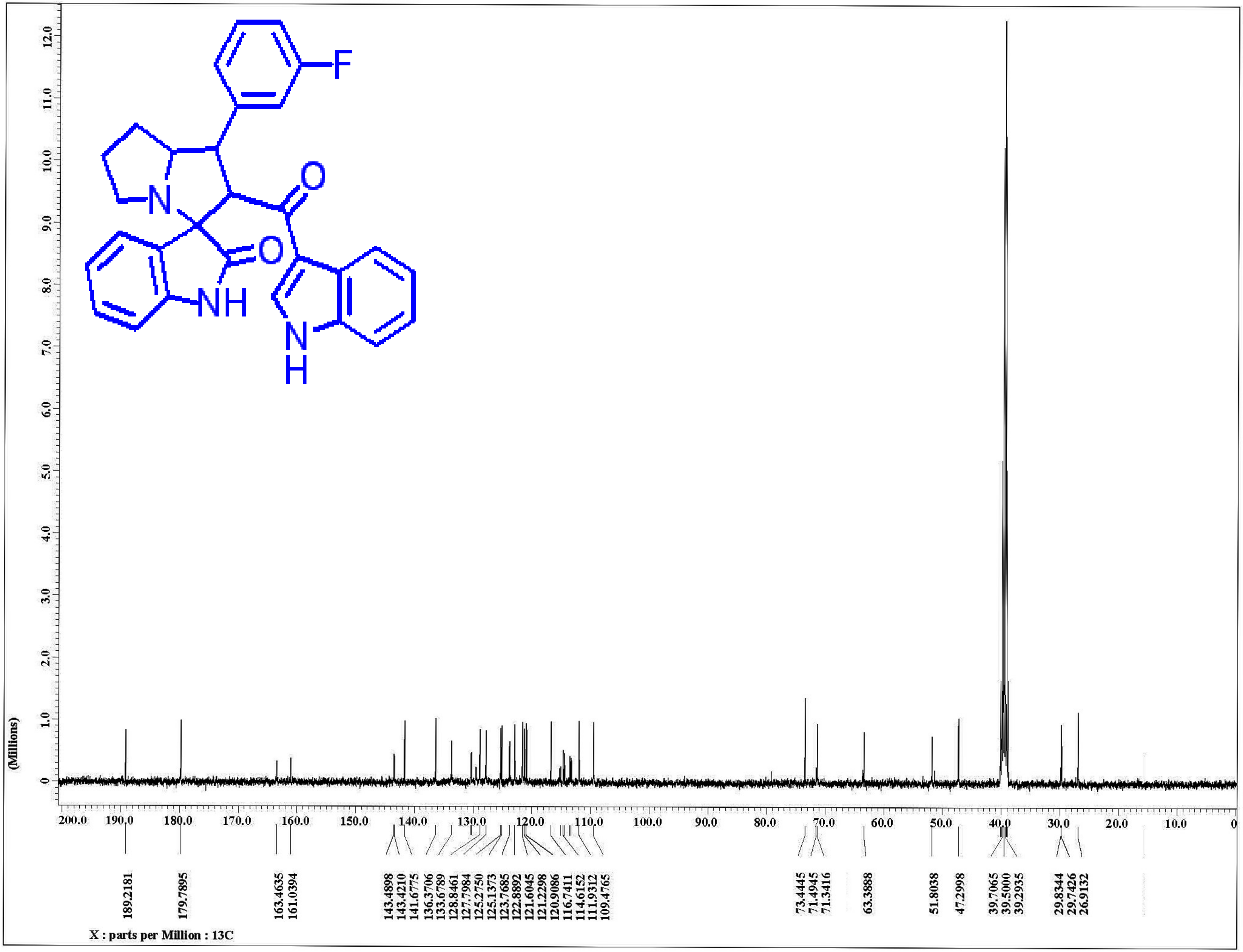

(Millions)
X : parts per Million : 13C
189.2181
179.7895
163.4635
161.0394
143.4898
143.4210
141.6775
136.3706
133.6789
128.8461
127.7984
125.2750
125.1373
123.7685
122.8892
121.6045
121.2298
120.9086
116.7411
114.6152
111.9312
109.4765
73.4445
71.4945
71.3416
63.3888
51.8038
47.2998
39.7065
39.5000
39.2935
29.8344
29.7426
26.9132

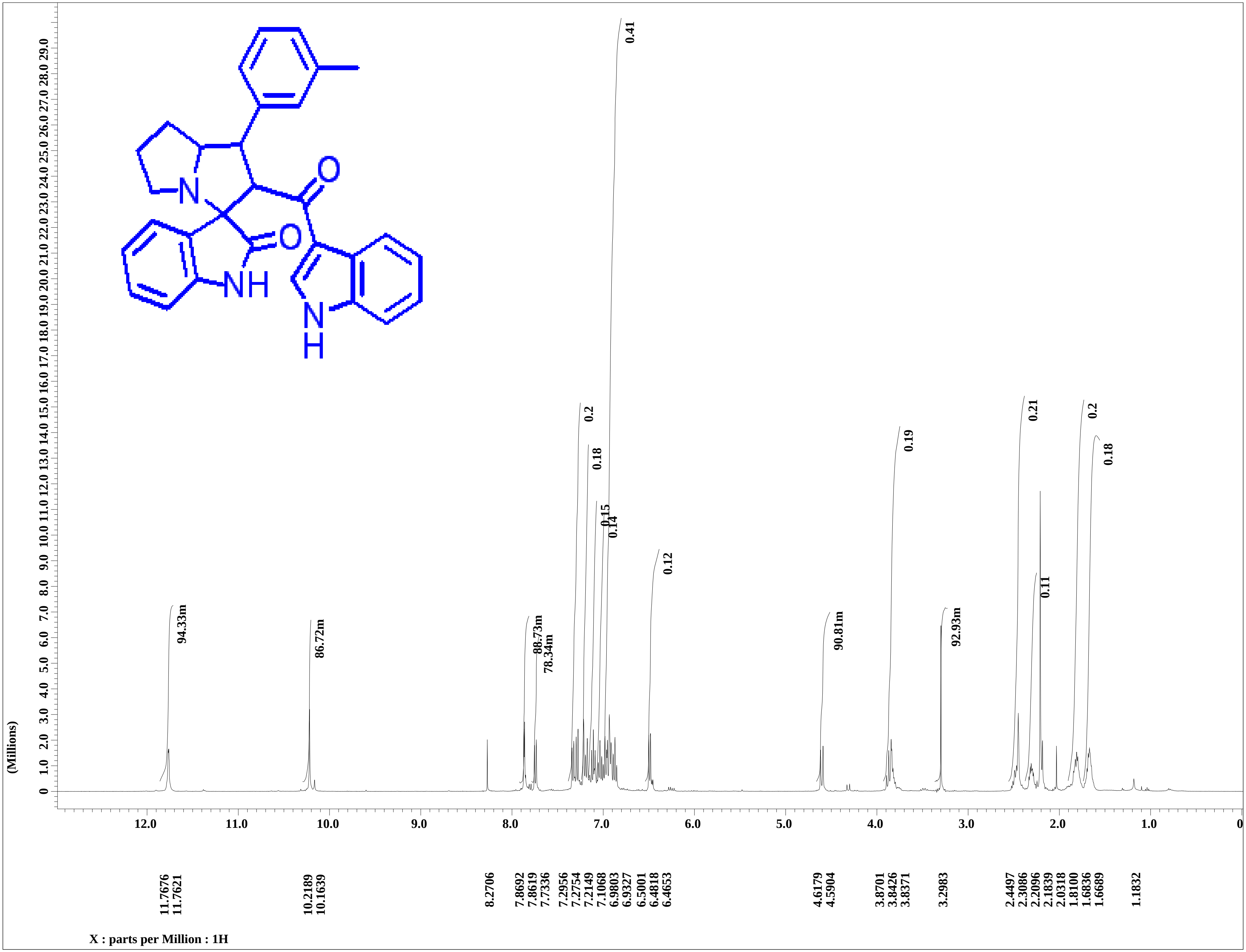

(Millions)
0
1.0
2.0
3.0
4.0
5.0
6.0
7.0
8.0
9.0
10.0
11.0
12.0
13.0
14.0
15.0
16.0
17.0
18.0
19.0
20.0
21.0
22.0
23.0
24.0
25.0
26.0
27.0
28.0
29.0
O
O
N
NH
N
H
94.33m
86.72m
88.73m
78.34m
0.2
0.18
0.15
0.14
0.41
0.12
90.81m
0.19
92.93m
0.21
0.11
0.2
0.18
12.0
11.0
10.0
9.0
8.0
7.0
6.0
5.0
4.0
3.0
2.0
1.0
0
11.7676
11.7621
10.2189
10.1639
8.2706
7.8692
7.8619
7.7336
7.2956
7.2754
7.2149
7.1068
6.9803
6.9327
6.5001
6.4818
6.4653
4.6179
4.5904
3.8701
3.8426
3.8371
3.2983
2.4497
2.3086
2.2096
2.1839
2.0318
1.8100
1.6836
1.6689
1.1832
X : parts per Million : 1H

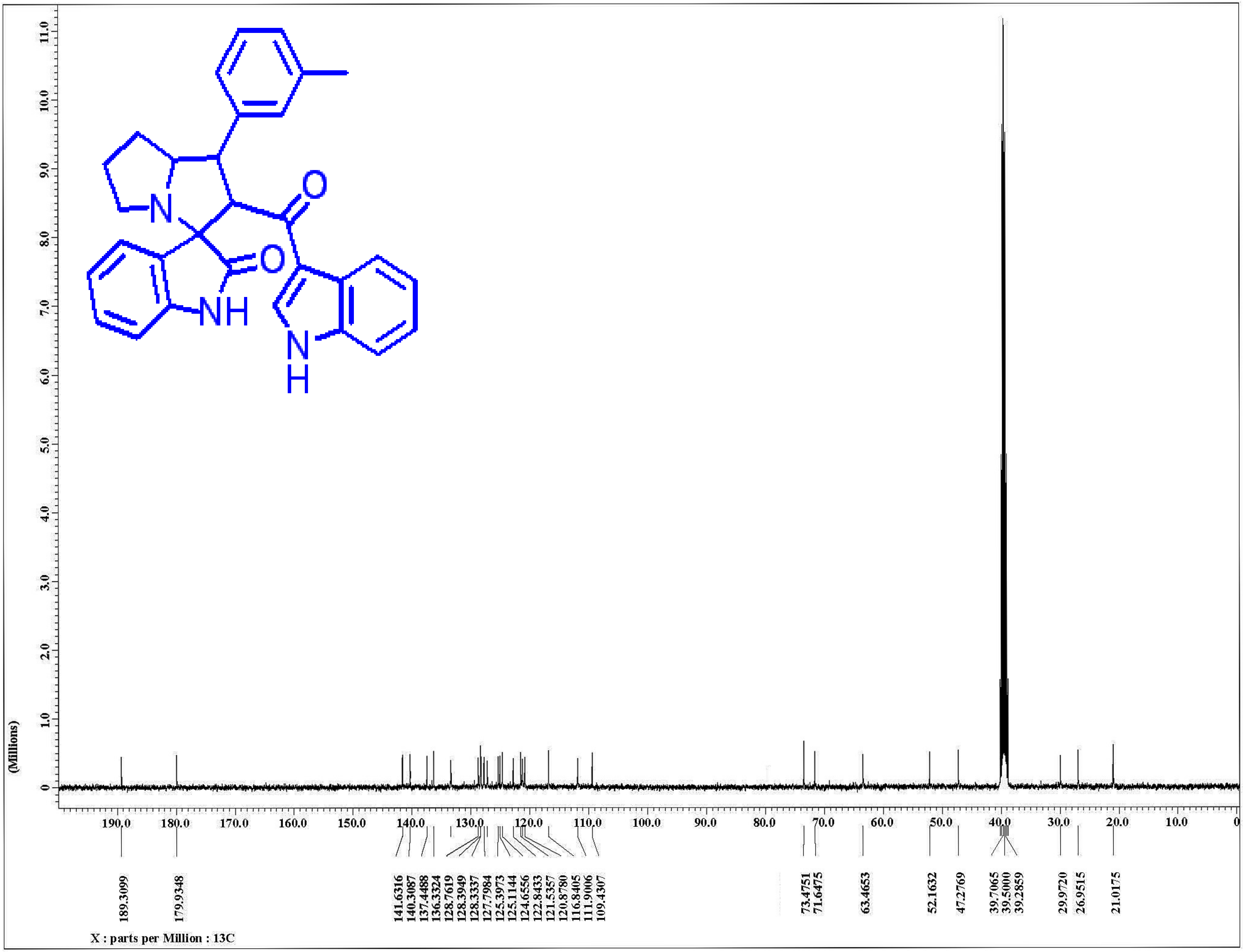

(Millions)
X : parts per Million : 13C
189.3099
179.9348
141.6316
140.3087
137.4488
136.3324
128.7619
128.3949
128.3337
127.7984
125.3973
125.1144
124.6556
122.8433
121.5357
120.8780
116.8405
111.9006
109.4307
73.4751
71.6475
63.4653
52.1632
47.2769
39.7065
39.5000
39.2859
29.9720
26.9515
21.0175

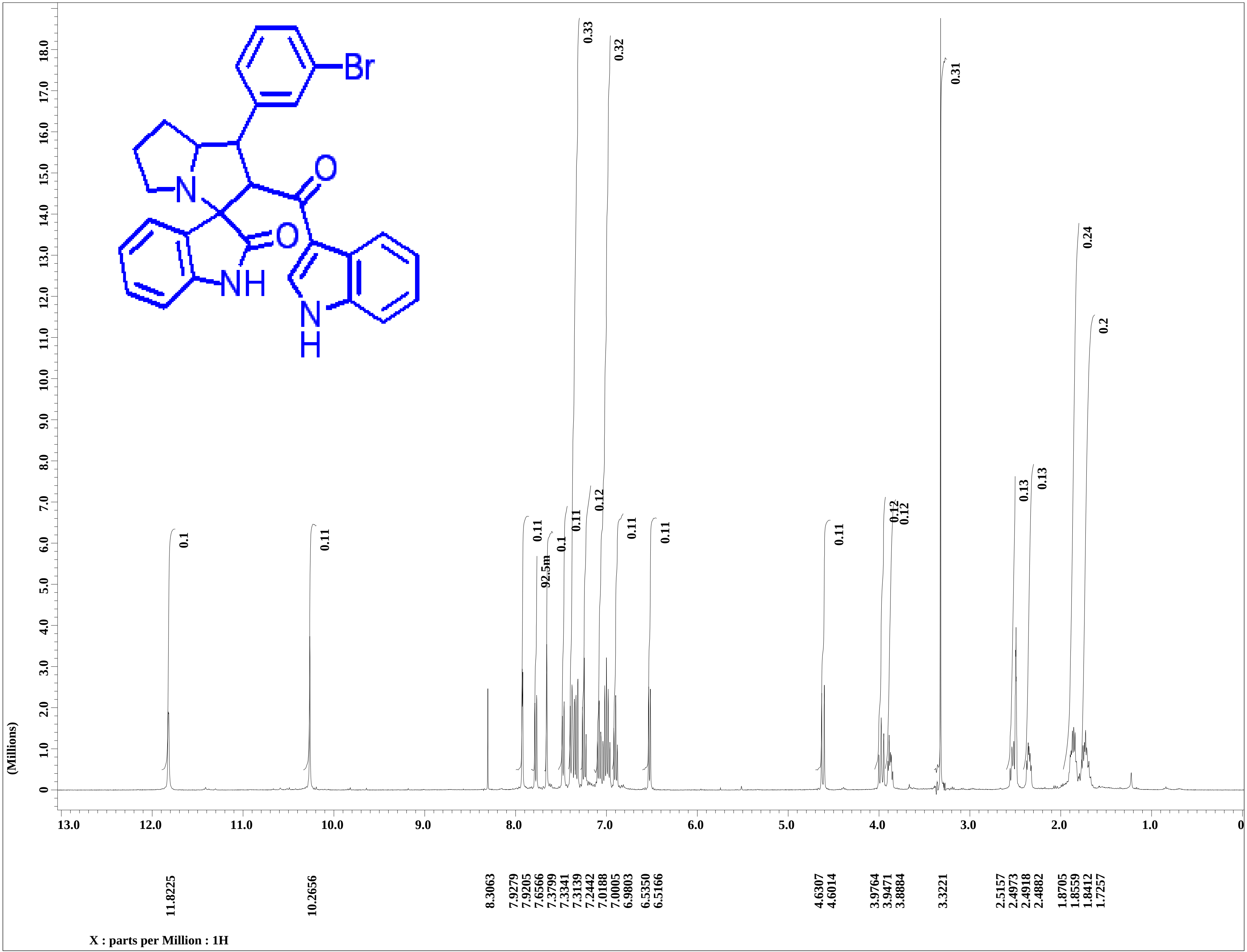

Br
O
O
N
NH
N
H
(Millions)
0.33
0.32
0.31
0.24
0.2
0.13
0.13
0.12
0.12
0.1
0.11
0.11
0.11
0.1
0.11
0.12
0.11
0.11
0.11
92.5m
13.0 12.0 11.0 10.0 9.0 8.0 7.0 6.0 5.0 4.0 3.0 2.0 1.0 0
11.8225
10.2656
8.3063
7.9279
7.9205
7.6566
7.3799
7.3341
7.3139
7.2442
7.0188
7.0005
6.9803
6.5350
6.5166
4.6307
4.6014
3.9764
3.9471
3.8884
3.3221
2.5157
2.4973
2.4918
2.4882
1.8705
1.8559
1.8412
1.7257
X : parts per Million : 1H

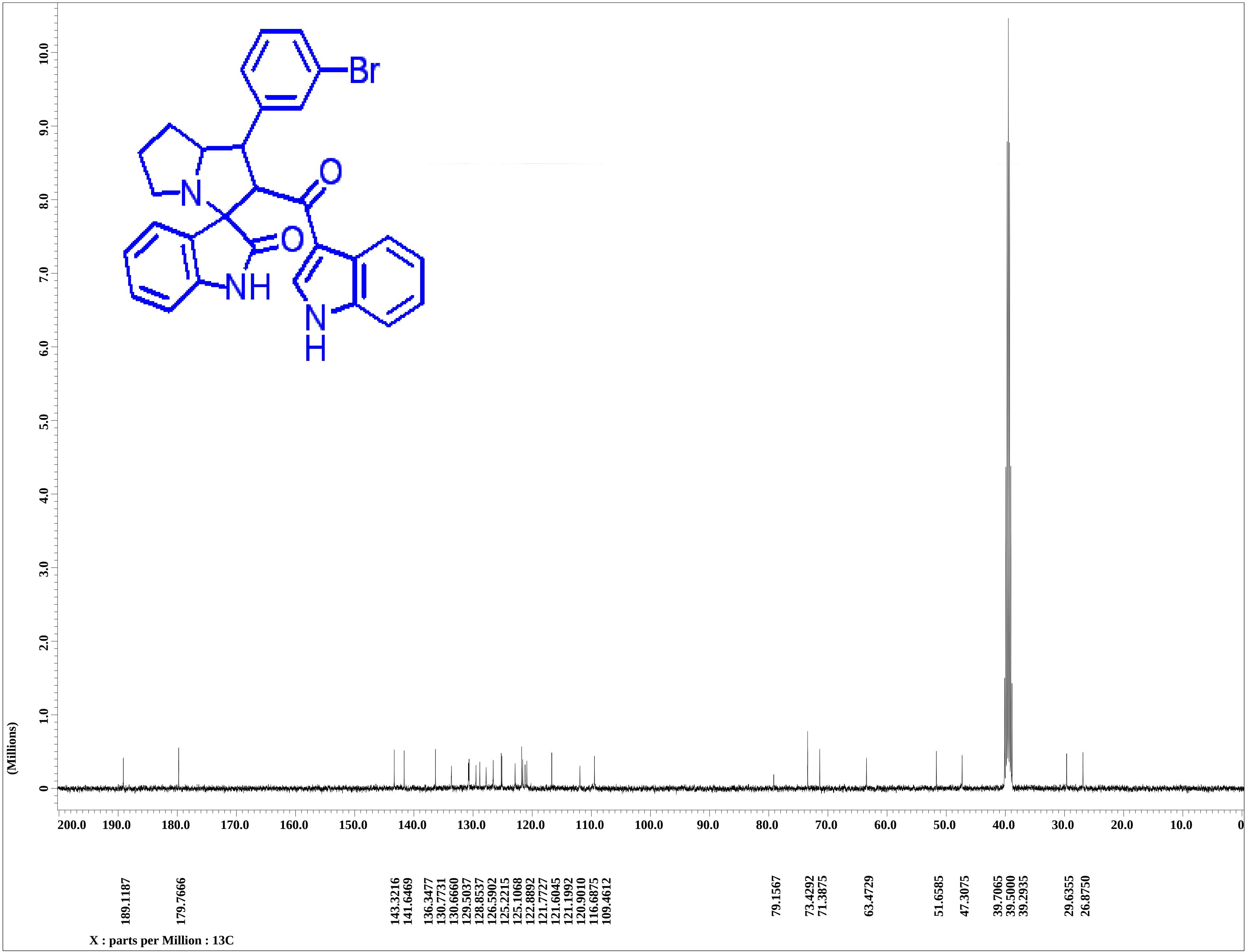

(Millions)
X : parts per Million : 13C
189.1187
179.7666
143.3216
141.6469
136.3477
130.7731
130.6660
129.5037
128.8537
126.5902
125.2215
125.1068
122.8892
121.7727
121.6045
121.1992
120.9010
116.6875
109.4612
79.1567
73.4292
71.3875
63.4729
51.6585
47.3075
39.7065
39.5000
39.2935
29.6355
26.8750

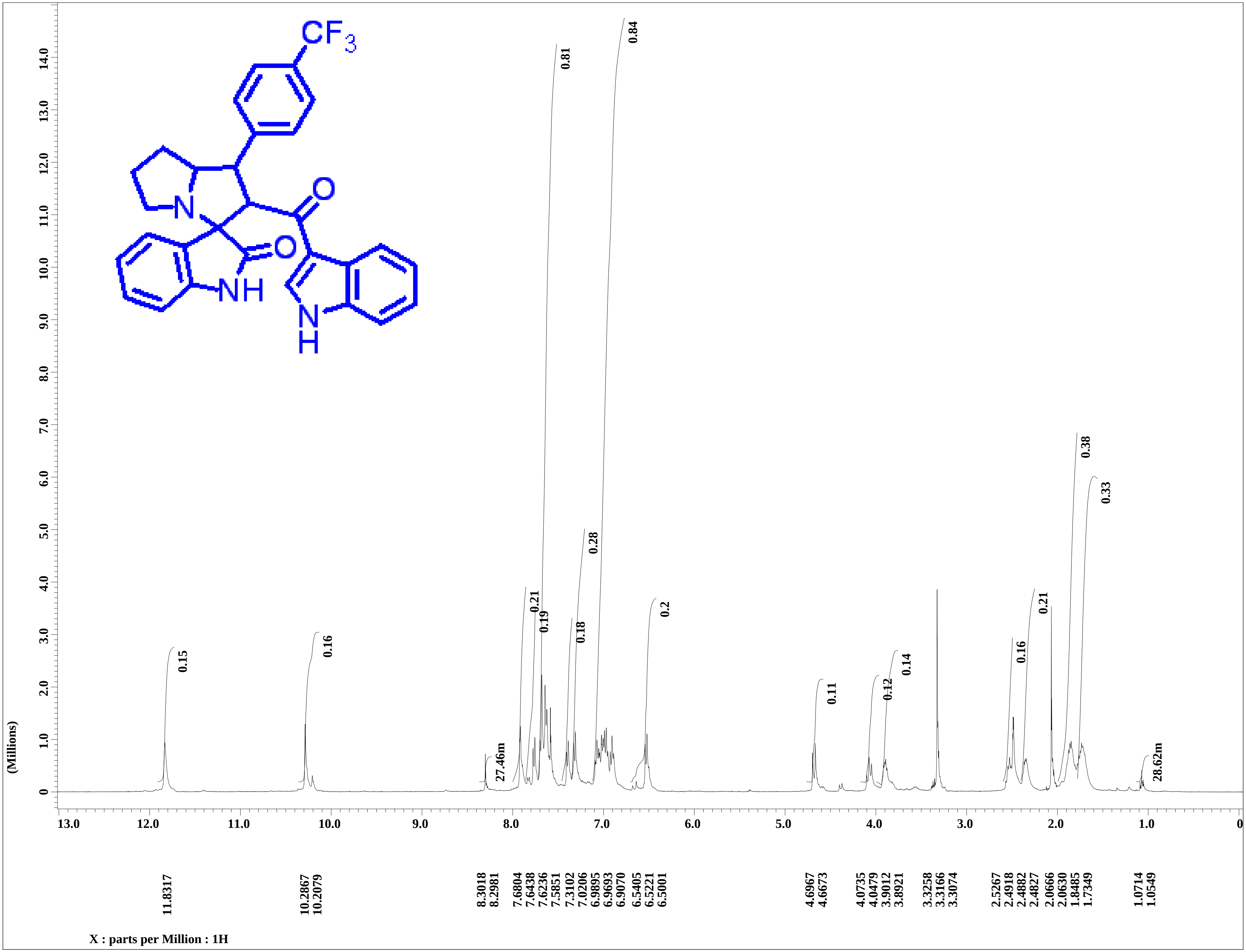
CF3
N
NH
O
O
N
H
(Millions)
X : parts per Million : 1H
11.8317
10.2867
10.2079
8.3018
8.2981
7.6804
7.6438
7.6236
7.5851
7.3102
7.0206
6.9895
6.9693
6.9070
6.5405
6.5221
6.5001
4.6967
4.6673
4.0735
4.0479
3.9012
3.8921
3.3258
3.3166
3.3074
2.5267
2.4918
2.4882
2.4827
2.0666
2.0630
1.8485
1.7349
1.0714
1.0549
0.15
0.16
27.46m
0.21
0.19
0.81
0.18
0.28
0.84
0.2
0.11
0.12
0.14
0.16
0.21
0.38
0.33
28.62m

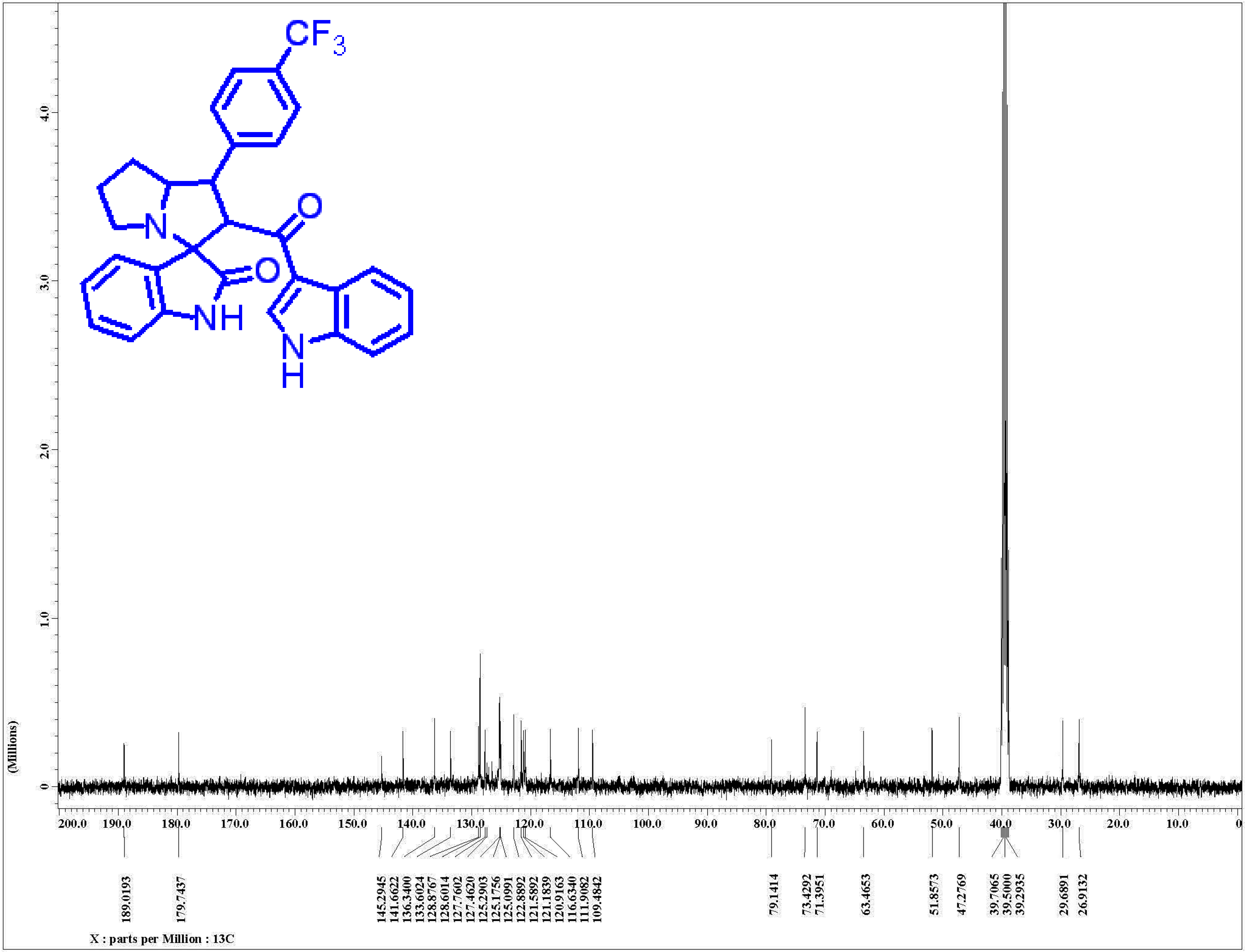

CF3
O
N
O
NH
N
H
(Millions)
4.0
3.0
2.0
1.0
0
200.0 190.0 180.0 170.0 160.0 150.0 140.0 130.0 120.0 110.0 100.0 90.0 80.0 70.0 60.0 50.0 40.0 30.0 20.0 10.0 0
189.0193
179.7437
145.2945
141.6622
136.3400
133.6024
128.8767
128.6014
127.7602
127.4620
125.2903
125.1756
125.0991
122.8892
121.5892
121.1839
120.9163
116.6340
111.9082
109.4842
79.1414
73.4292
71.3951
63.4653
51.8573
47.2769
39.7065
39.5000
39.2935
29.6891
26.9132
X : parts per Million : 13C

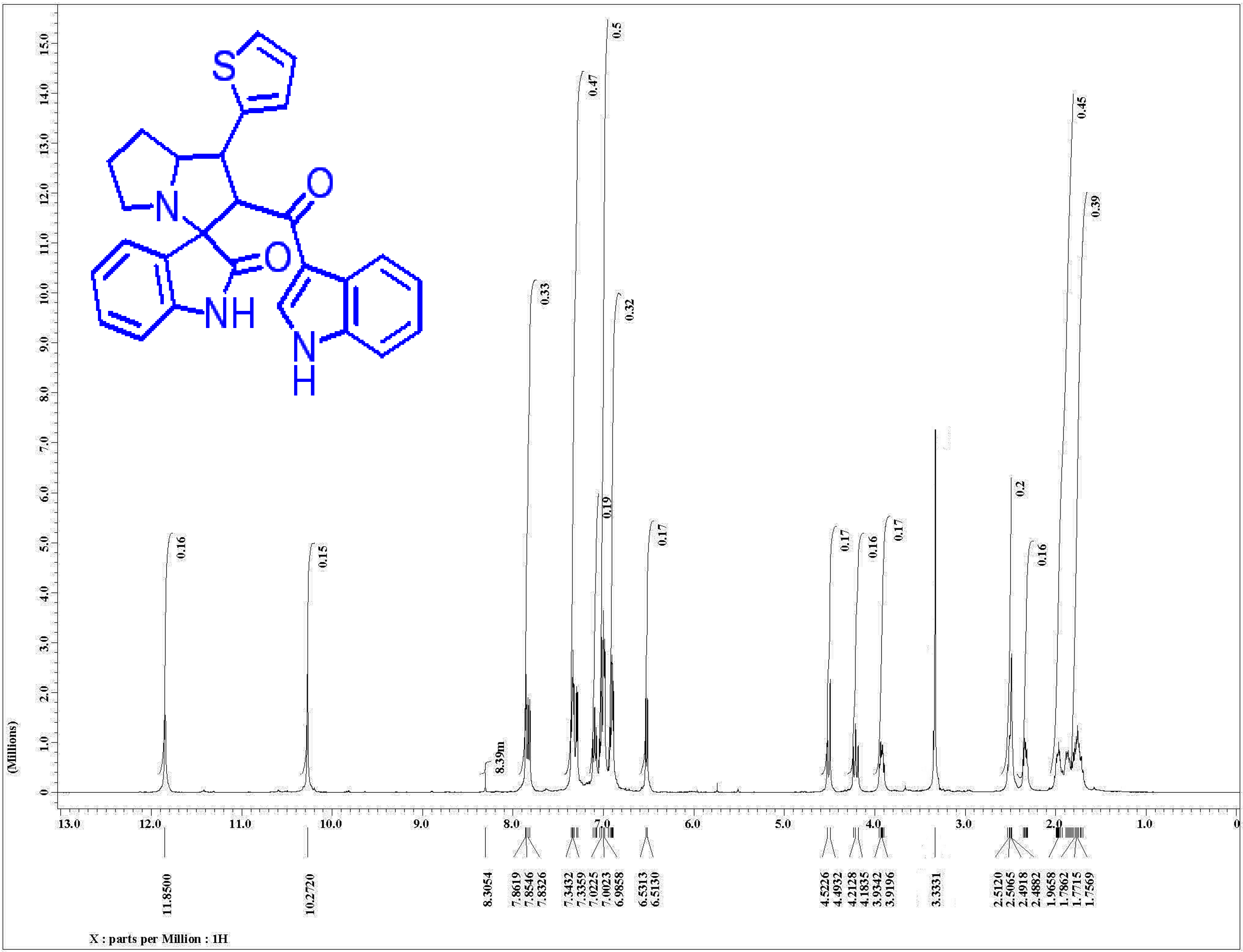
S
N
NH
O
O
N
H
(Millions)
X : parts per Million : 1H
11.8500
10.2720
8.3054
8.39m
7.8619
7.8546
7.8326
7.3432
7.3359
7.0225
7.0023
6.9858
6.5313
6.5130
4.5226
4.4932
4.2128
4.1835
3.9342
3.9196
3.3331
2.5120
2.5065
2.4918
2.4882
1.9658
1.7862
1.7715
1.7569
0.16
0.15
0.33
0.47
0.5
0.19
0.32
0.17
0.17
0.16
0.17
0.2
0.16
0.45
0.39

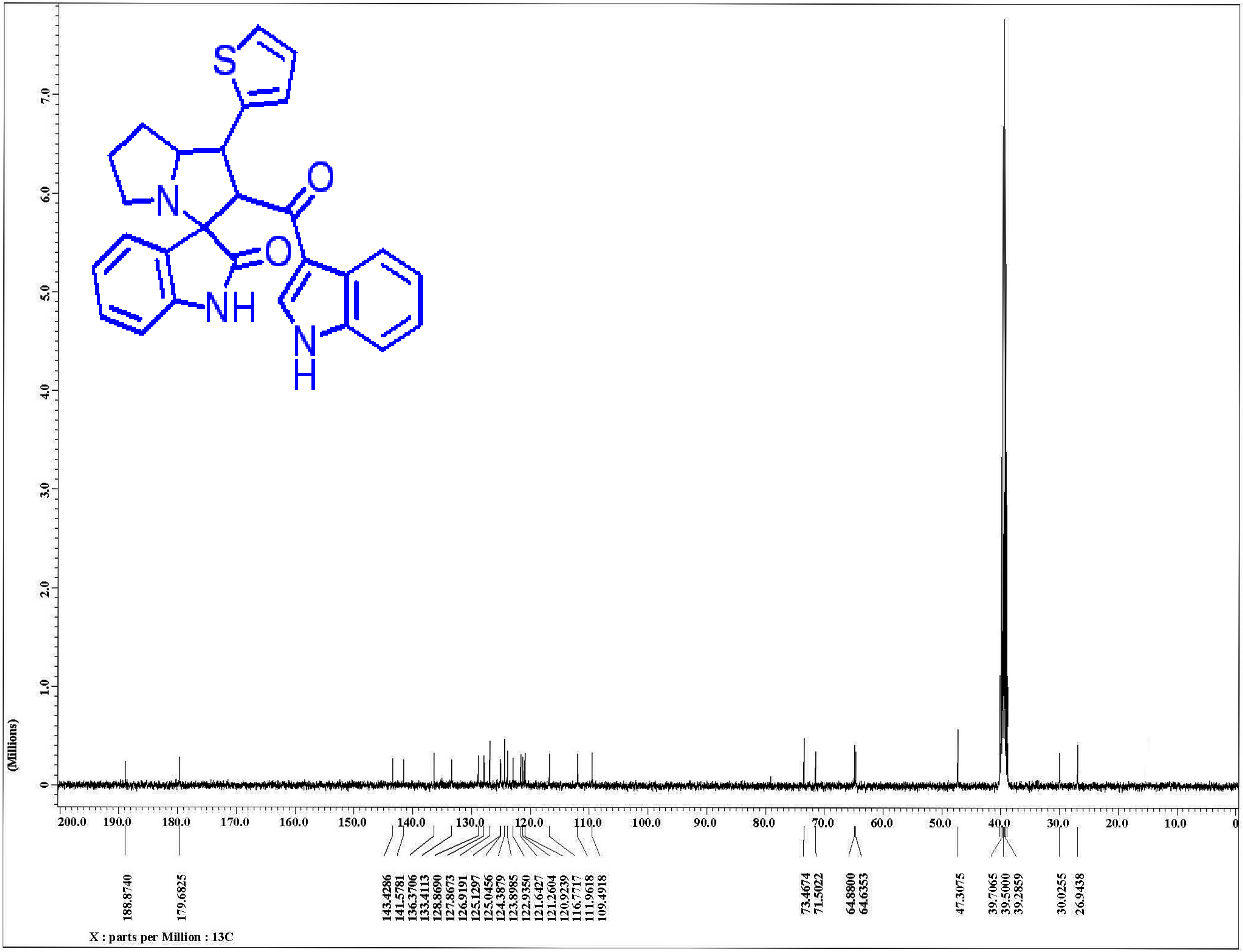

(Millions)
7.0
6.0
5.0
4.0
3.0
2.0
1.0
0
S
N
O
O
NH
N
H
200.0 190.0 180.0 170.0 160.0 150.0 140.0 130.0 120.0 110.0 100.0 90.0 80.0 70.0 60.0 50.0 40.0 30.0 20.0 10.0 0
188.8740
179.6825
143.4286
141.5781
136.3706
133.4113
128.8690
127.8673
126.9191
125.1297
125.0456
124.3879
123.8985
122.9350
121.6427
121.2604
120.9239
116.7717
111.9618
109.4918
73.4674
71.5022
64.8800
64.6353
47.3075
39.7065
39.5000
39.2859
30.0255
26.9438
X : parts per Million : 13C

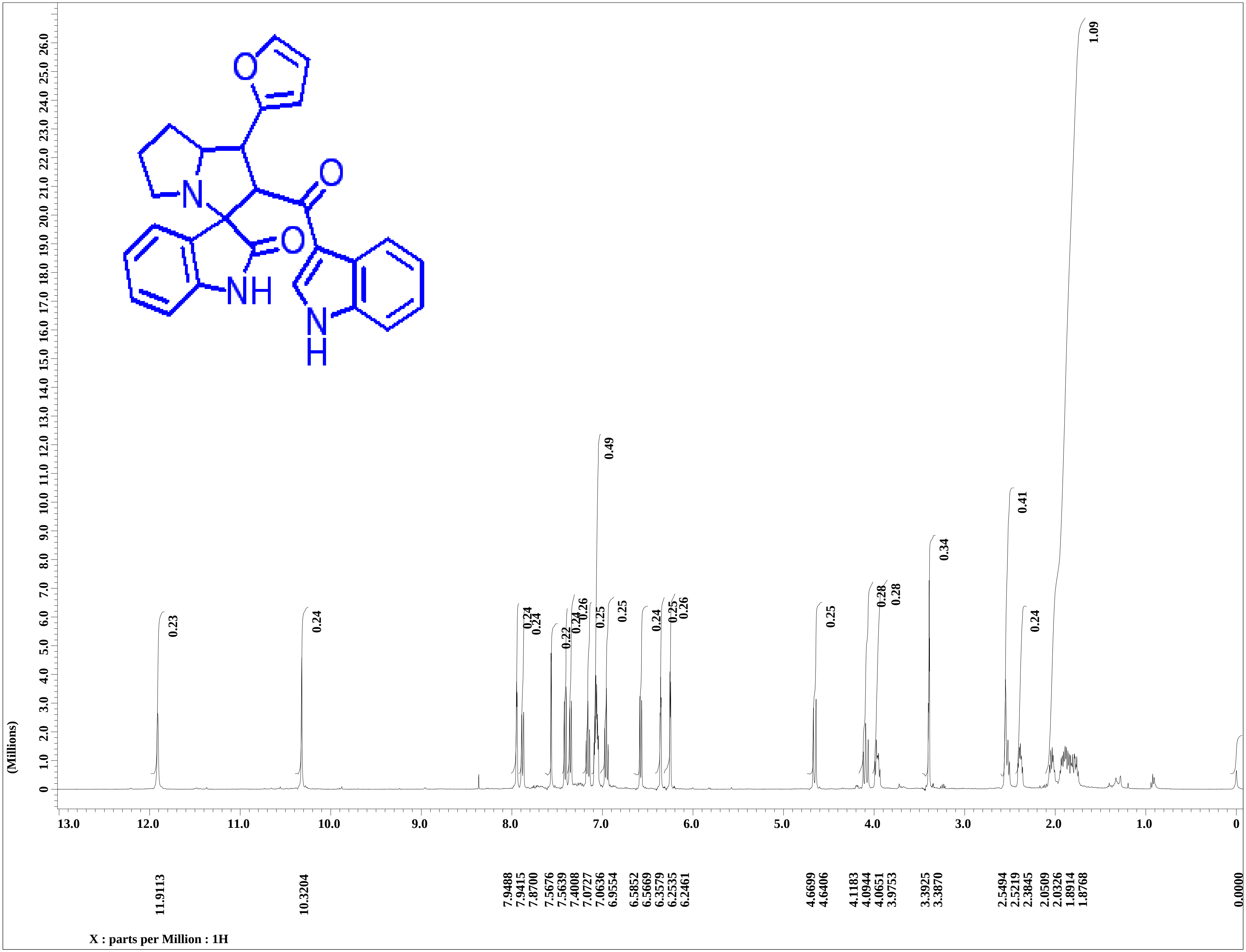
(Millions)
X : parts per Million : 1H
11.9113
10.3204
7.9488
7.9415
7.8700
7.5676
7.5639
7.4008
7.0727
7.0636
6.9554
6.5852
6.5669
6.3579
6.2535
6.2461
4.6699
4.6406
4.1183
4.0944
4.0651
3.9753
3.3925
3.3870
2.5494
2.5219
2.3845
2.0509
2.0326
1.8914
1.8768
0.0000
NH
N
O
H

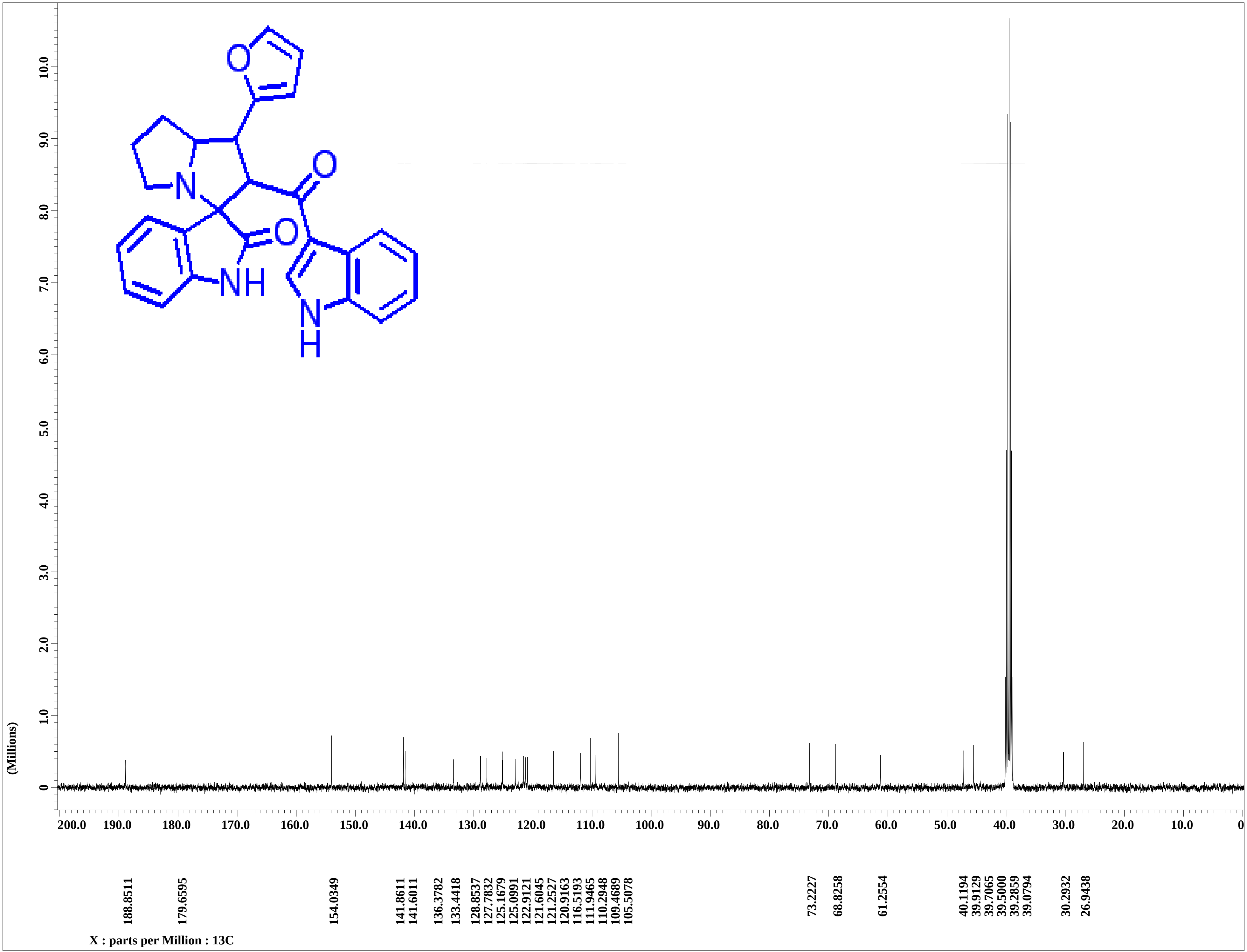

(Millions)
10.0
9.0
8.0
7.0
6.0
5.0
4.0
3.0
2.0
1.0
0
200.0 190.0 180.0 170.0 160.0 150.0 140.0 130.0 120.0 110.0 100.0 90.0 80.0 70.0 60.0 50.0 40.0 30.0 20.0 10.0 0
188.8511
179.6595
154.0349
141.8611
141.6011
136.3782
133.4418
128.8537
127.7832
125.1679
125.0991
122.9121
121.6045
121.2527
120.9163
116.5193
111.9465
110.2948
109.4689
105.5078
73.2227
68.8258
61.2554
40.1194
39.9129
39.7065
39.5000
39.2859
39.0794
30.2932
26.9438
X : parts per Million : 13C
O
N
O
NH
O
N
H

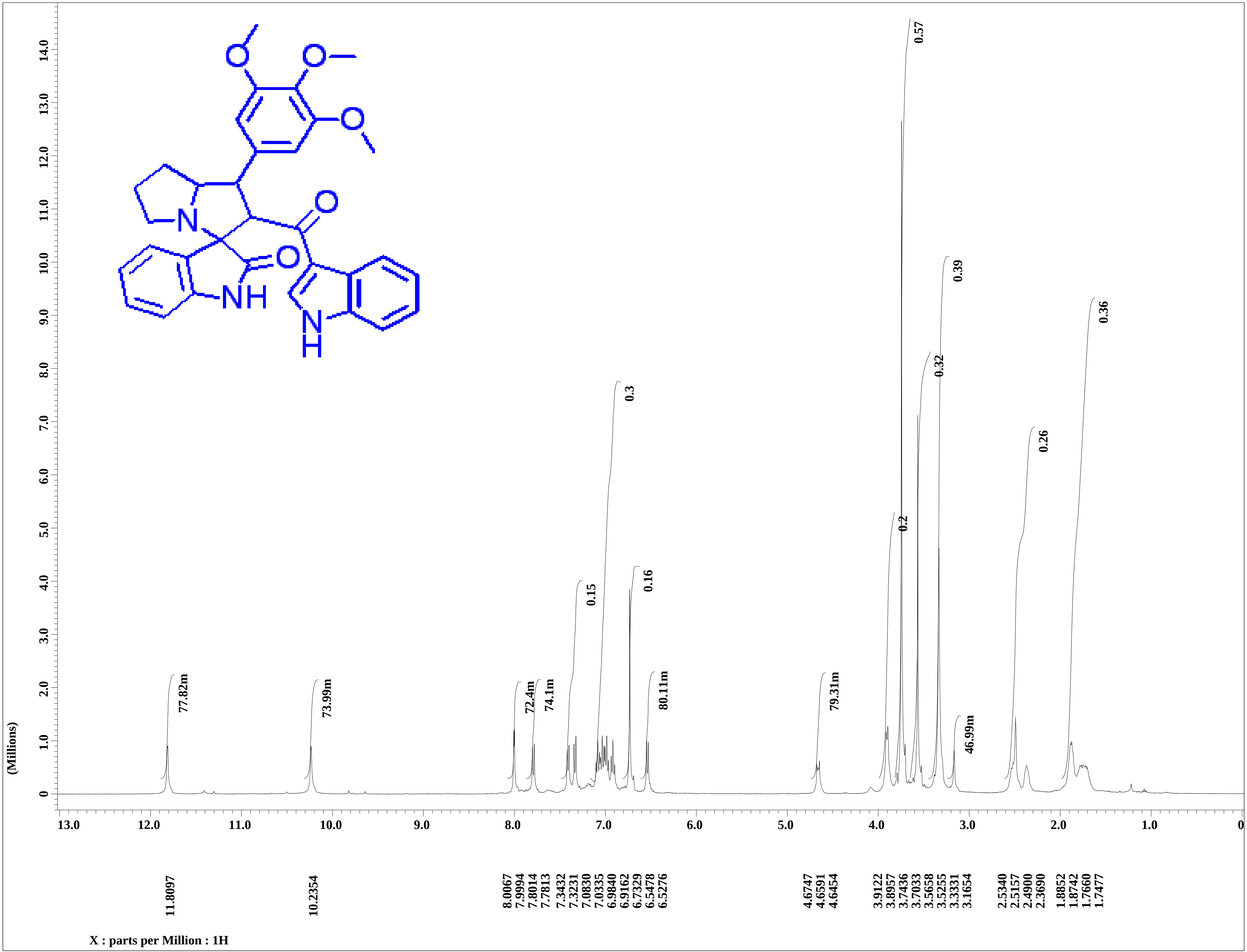

(Millions)
0
1.0
2.0
3.0
4.0
5.0
6.0
7.0
8.0
9.0
10.0
11.0
12.0
13.0
14.0
77.82m
73.99m
72.4m
74.1m
0.15
0.3
0.16
80.11m
79.31m
0.2
0.57
0.32
0.39
46.99m
0.26
0.36
13.0
12.0
11.0
10.0
9.0
8.0
7.0
6.0
5.0
4.0
3.0
2.0
1.0
0
11.8097
10.2354
8.0067
7.9994
7.8014
7.7813
7.3432
7.3231
7.0830
7.0335
6.9840
6.9162
6.7329
6.5478
6.5276
4.6747
4.6591
4.6454
3.9122
3.8957
3.7436
3.7033
3.5658
3.5255
3.3331
3.1654
2.5340
2.5157
2.4900
2.3690
1.8852
1.8742
1.7660
1.7477
X : parts per Million : 1H

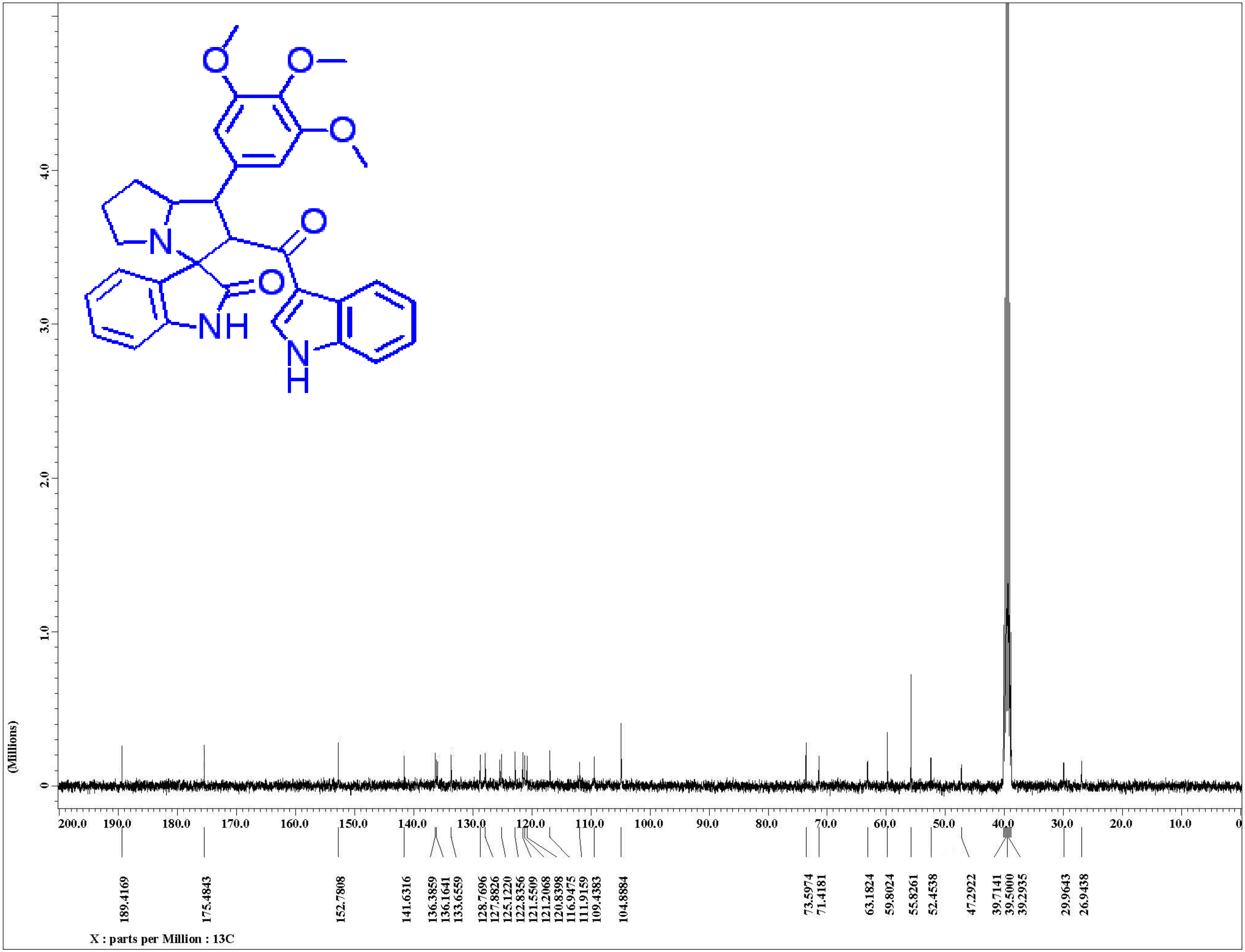

4.0
3.0
2.0
1.0
0
(Millions)
200.0 190.0 180.0 170.0 160.0 150.0 140.0 130.0 120.0 110.0 100.0 90.0 80.0 70.0 60.0 50.0 40.0 30.0 20.0 10.0 0
189.4169
175.4843
152.7808
141.6316
136.3859
136.1641
133.6559
128.7696
127.8826
125.1220
122.8356
121.5509
121.2068
120.8398
116.9475
111.9159
109.4383
104.8884
73.5974
71.4181
63.1824
59.8024
55.8261
52.4538
47.2922
39.7141
39.5000
39.2935
29.9643
26.9438
X : parts per Million : 13C

	Molecule				VIDA ID	PLP	Chemgauss3	OEChemscore	Screenscore	Consensus Score
1	4d_14 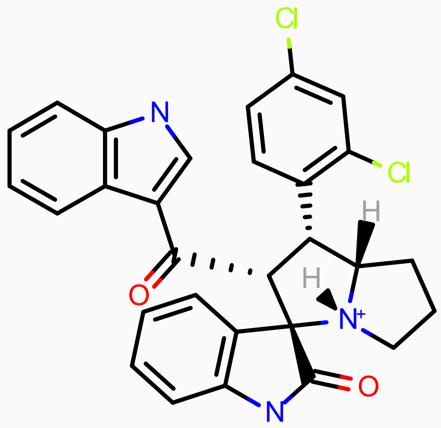	·	·	·	2	-43.8969	-47.0635	-37.5086	-111.8020	19.0000
2	4m_31 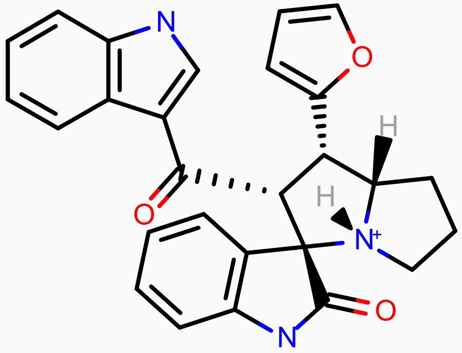	·	·	·	3	-39.6301	-48.1326	-36.6915	-116.3491	25.0000

	Molecule				VIDA ID	PLP	Chemgauss3	OEChemscore	Screenscore	Consensus Score
3	4l_18 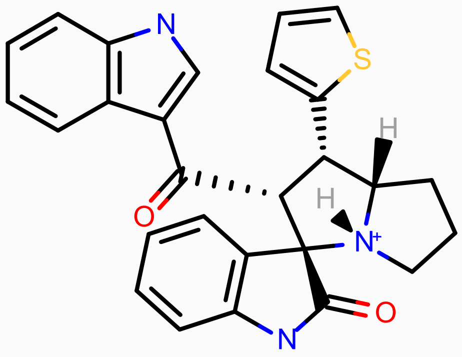	·	·	·	4	-43.2531	-46.2579	-35.8414	-108.6577	28.0000
4	4j_6 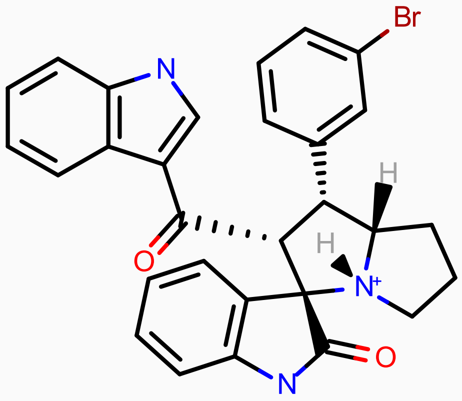	·	·	·	5	-49.8619	-40.1485	-35.7006	-118.3784	36.0000

	Molecule				VIDA ID	PLP	Chemgauss3	OEChemscore	Screenscore	Consensus Score
5	5l_32 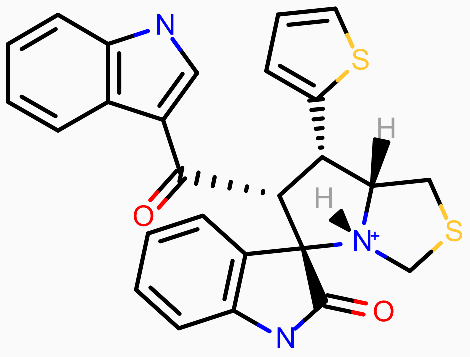	·	·	·	6	-40.9698	-51.1818	-34.2032	-98.4348	38.0000
6	4b_3 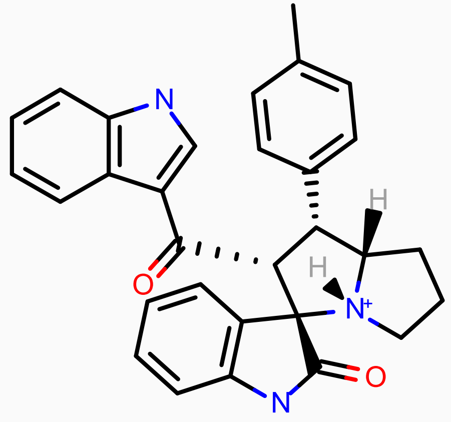	·	·	·	7	-42.8749	-43.2095	-36.8631	-106.8663	39.0000

	Molecule				VIDA ID	PLP	Chemgauss3	OEChemscore	Screenscore	Consensus Score
7	5m_51 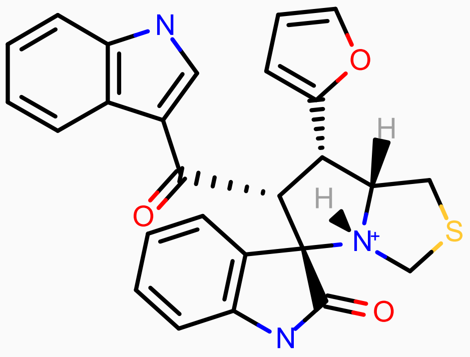	·	·	·	8	-38.9010	-47.8791	-33.5604	-112.7466	39.0000
8	4e_11 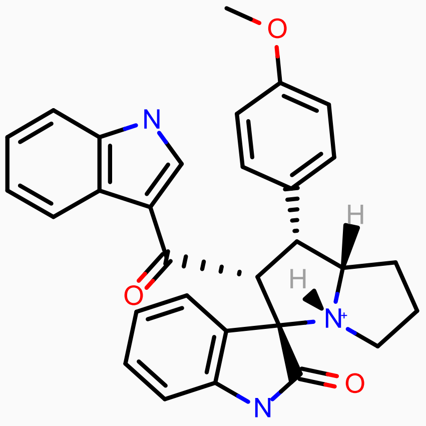	·	·	·	9	-46.8264	-42.1867	-36.7480	-107.3709	40.0000

	Molecule				VIDA ID	PLP	Chemgauss3	OEChemscore	Screenscore	Consensus Score
9	4n_6 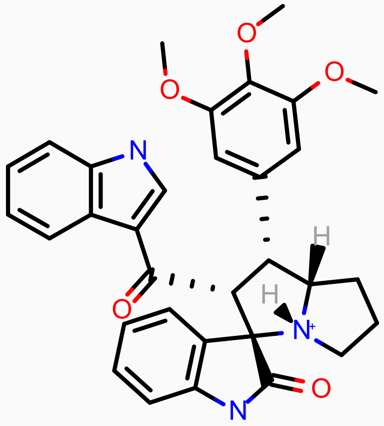	·	·	·	10	-49.8126	-36.6365	-35.8107	-124.9373	41.0000
10	4k_3 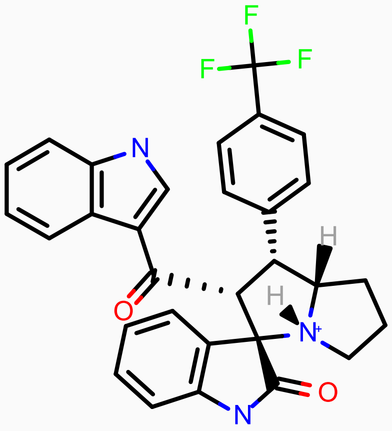	·	·	·	11	-44.0345	-44.3111	-32.1663	-112.3442	42.0000

	Molecule				VIDA ID	PLP	Chemgauss3	OEChemscore	Screenscore	Consensus Score
11	4h_17 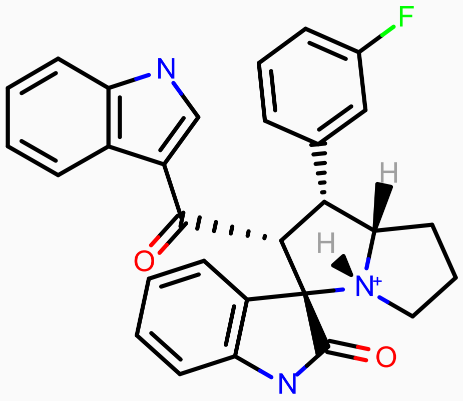	·	·	·	12	-47.2948	-39.5843	-33.4473	-115.7956	50.0000
12	5d_12 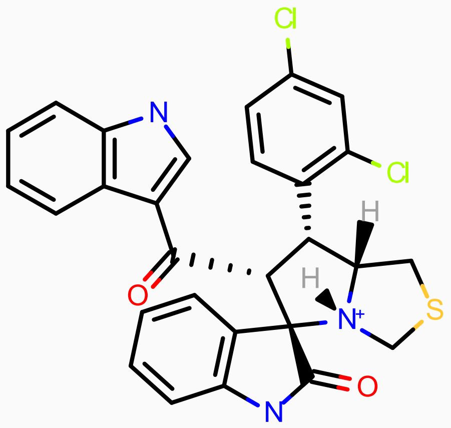	·	·	·	13	-32.2426	-51.3031	-36.9214	-93.1185	50.0000

	Molecule				VIDA ID	PLP	Chemgauss3	OEChemscore	Screenscore	Consensus Score
13	5n_3 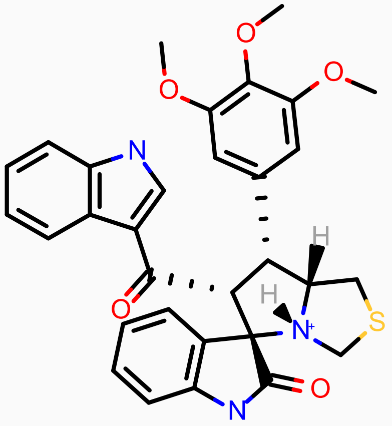	·	·	·	14	-36.5671	-44.1508	-35.2216	-93.2587	59.0000
14	4f_1 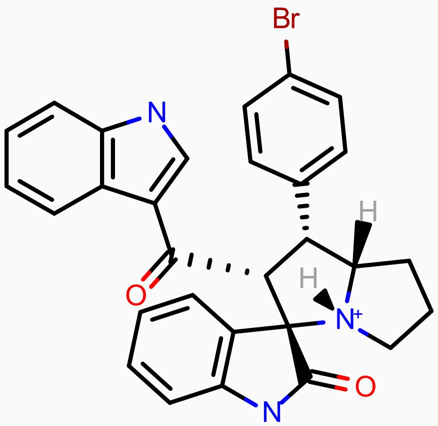	·	·	·	15	-39.4647	-39.8280	-33.1119	-107.6050	71.0000

	Molecule				VIDA ID	PLP	Chemgauss3	OEChemscore	Screenscore	Consensus Score
15	5LAW-Lig_7 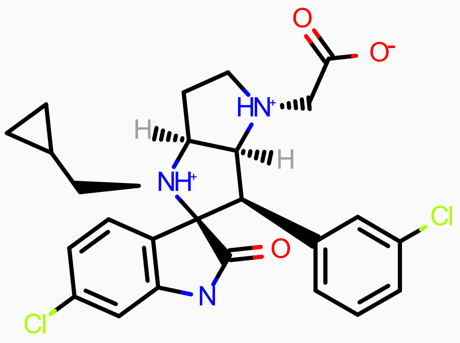	·	·	·	16	-37.2815	-50.3208	-31.8426	-84.3965	73.0000
16	5a_6 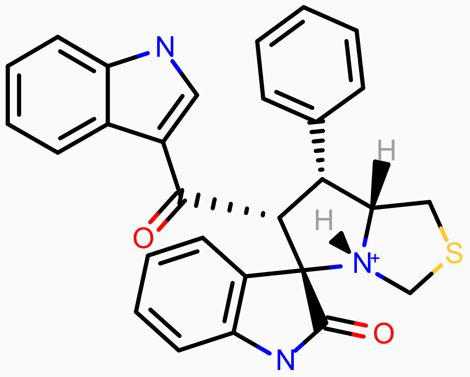	·	·	·	17	-33.1745	-43.8700	-32.7218	-95.3957	74.0000

	Molecule				VIDA ID	PLP	Chemgauss3	OEChemscore	Screenscore	Consensus Score
17	st1_66 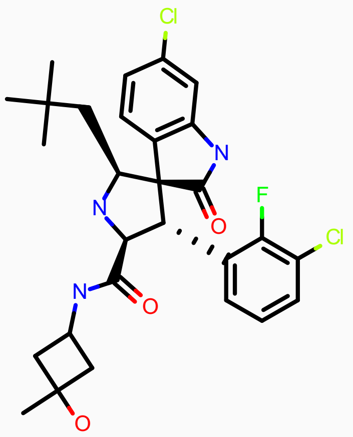	·	·	·	18	-36.7414	-44.1902	-31.4586	-94.0793	74.0000
18	5h_9 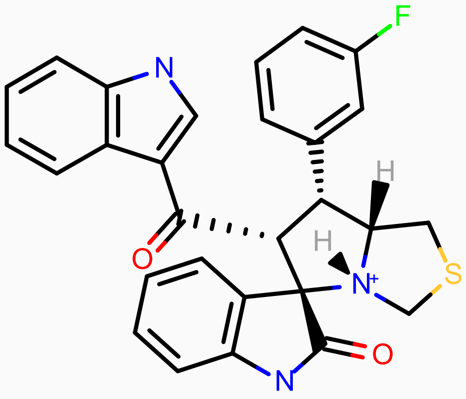	·	·	·	19	-41.0206	-35.6290	-35.9141	-84.9015	76.0000

	Molecule				VIDA ID	PLP	Chemgauss3	OEChemscore	Screenscore	Consensus Score
19	st7_3 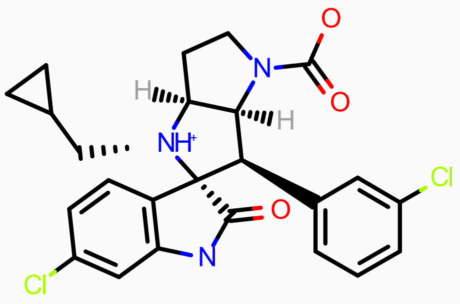	·	·	·	20	-33.3921	-51.2094	-27.8704	-94.1628	77.0000
20	4a_6 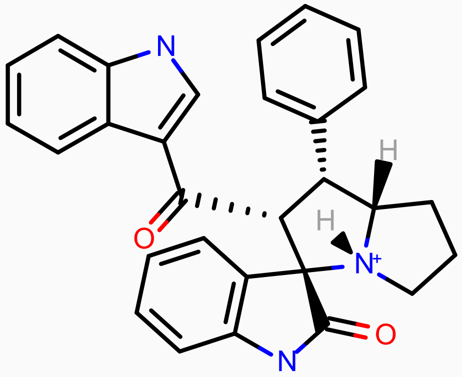	·	·	·	21	-34.3374	-43.0117	-33.9697	-85.6030	78.0000

	Molecule				VIDA ID	PLP	Chemgauss3	OEChemscore	Screenscore	Consensus Score
21	st4_44 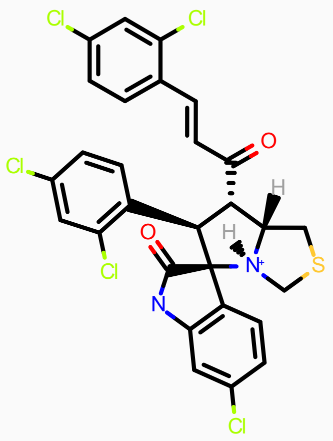	·	·	·	22	-42.7818	-39.8367	-30.2014	-104.7276	78.0000
22	5f_1 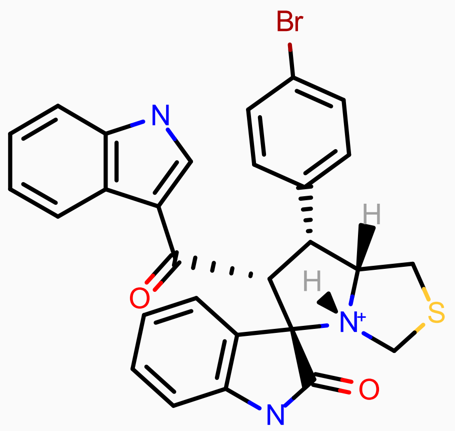	·	·	·	23	-33.1035	-43.3786	-33.4818	-86.6951	80.0000

	Molecule				VIDA ID	PLP	Chemgauss3	OEChemscore	Screenscore	Consensus Score
23	st5_23 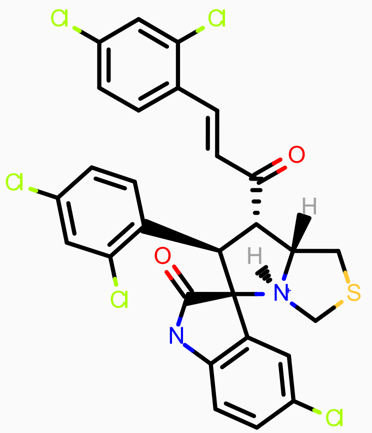	·	·	·	24	-42.2645	-38.7011	-29.3169	-108.2522	81.0000
24	5j_2 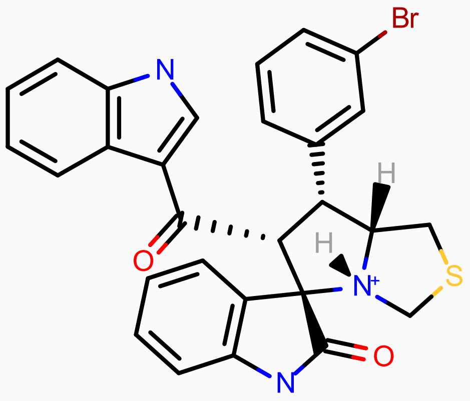	·	·	·	25	-34.2389	-43.5267	-33.6259	-81.3622	82.0000

	Molecule				VIDA ID	PLP	Chemgauss3	OEChemscore	Screenscore	Consensus Score
25	4i_12 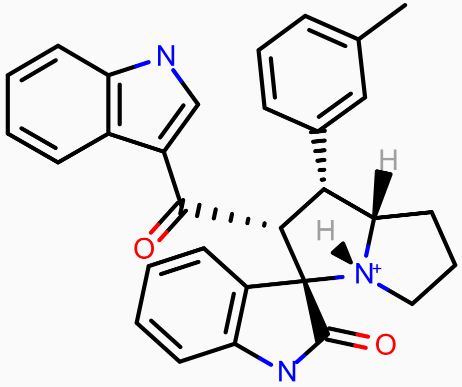	·	·	·	26	-35.0521	-43.6876	-31.8044	-90.2029	82.0000
26	5b_6 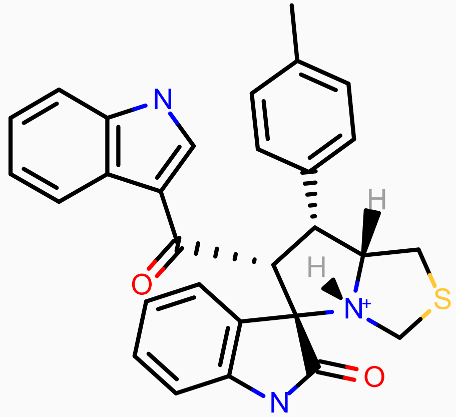	·	·	·	27	-31.1570	-43.8512	-33.1818	-85.8385	84.0000

	Molecule				VIDA ID	PLP	Chemgauss3	OEChemscore	Screenscore	Consensus Score
27	4g_6 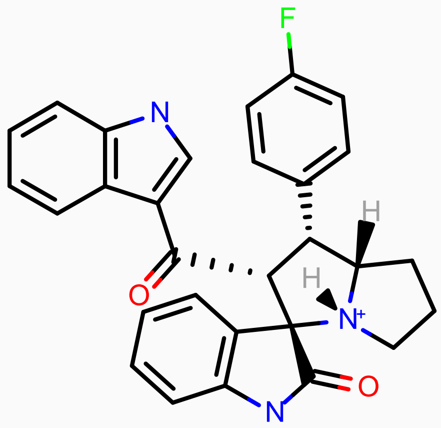	·	·	·	28	-42.5365	-36.5579	-30.6523	-97.2936	86.0000
28	4c_3 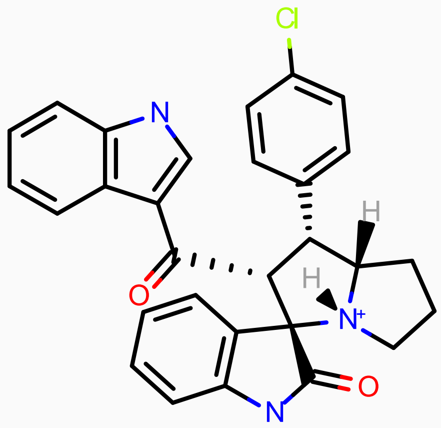	·	·	·	29	-33.7504	-43.4569	-30.7180	-85.7023	92.0000

	Molecule				VIDA ID	PLP	Chemgauss3	OEChemscore	Screenscore	Consensus Score
29	st2_73 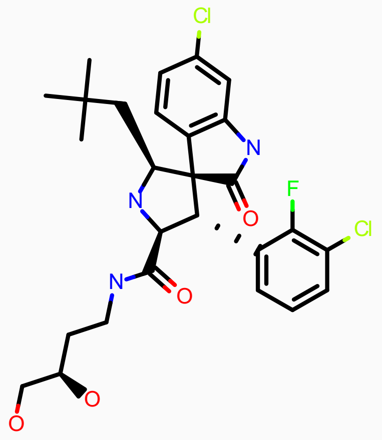	·	·	·	30	-38.8238	-43.0550	-32.0881	-69.8469	96.0000
30	5i_1 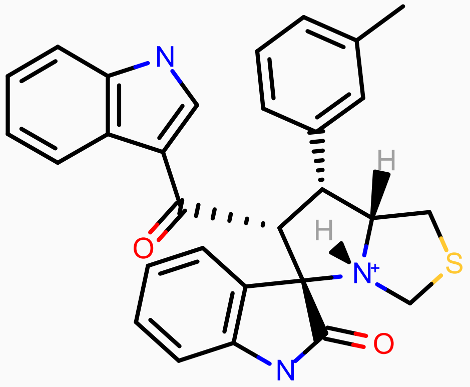	·	·	·	31	-32.6082	-42.3921	-33.2965	-82.0229	97.0000

	Molecule				VIDA ID	PLP	Chemgauss3	OEChemscore	Screenscore	Consensus Score
31	5k_1 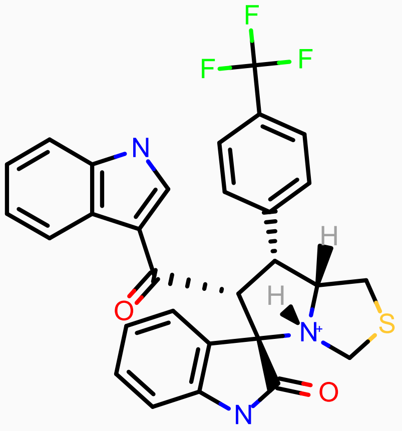	·	·	·	32	-24.8977	-51.7531	-27.6202	-83.3487	98.0000
32	st3_176 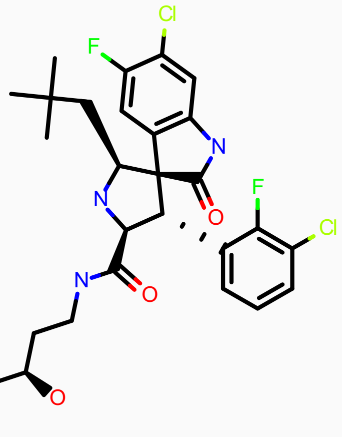	·	·	·	33	-37.1112	-30.5460	-33.1862	-82.3946	100.0000

	Molecule				VIDA ID	PLP	Chemgauss3	OEChemscore	Screenscore	Consensus Score
33	5g_6 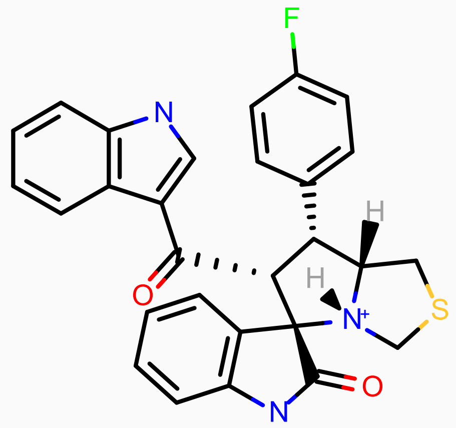	·	·	·	34	-28.9542	-42.4108	-32.2288	-83.4253	101.0000
34	5e_10 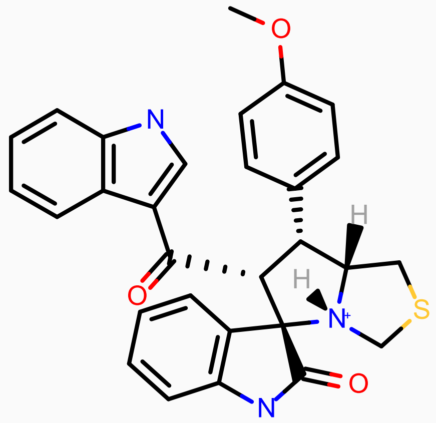	·	·	·	35	-22.5473	-50.6644	-28.1813	-73.9543	107.0000

	Molecule				VIDA ID	PLP	Chemgauss3	OEChemscore	Screenscore	Consensus Score
35	5c_1 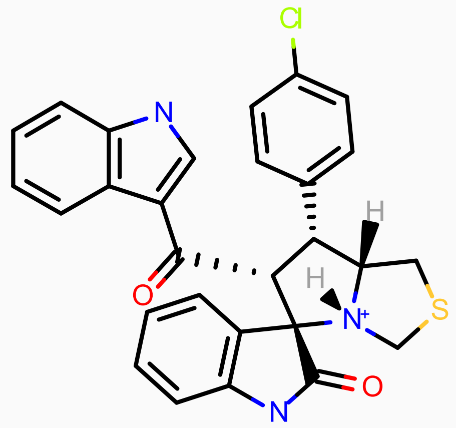	·	·	·	36	-28.2499	-41.9340	-32.1666	-80.8915	111.0000
36	st6_64 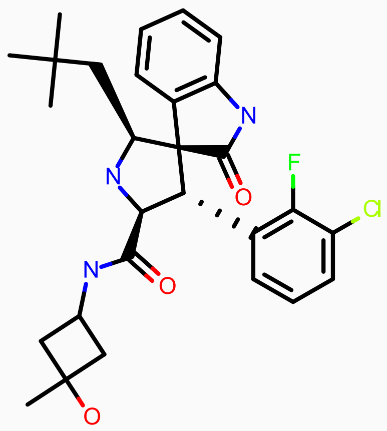	·	·	·	37	-28.0238	-41.4120	-28.8875	-75.0506	124.0000
37	st8_15 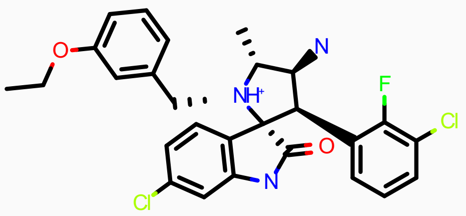	·	·	·	38	-27.9871	-38.7313	-27.3032	-70.0984	136.0000
